# Selective hydrolysis of α-oxo ketene *N*,*S*-acetals in water: switchable aqueous synthesis of β-keto thioesters and β-keto amides

**DOI:** 10.3762/bjoc.20.190

**Published:** 2024-09-03

**Authors:** Haifeng Yu, Wanting Zhang, Xuejing Cui, Zida Liu, Xifu Zhang, Xiaobo Zhao

**Affiliations:** 1 College of Chemistry, Baicheng Normal University, Baicheng, Jilin 137000, China

**Keywords:** α-oxo ketene *N*,*S*-acetals, β-keto amide, β-keto thioester, dodecylbenzenesulfonic acid, hydrolysis

## Abstract

An eco-friendly selective hydrolysis of chain α-oxo ketene *N*,*S*-acetals in water for the switchable synthesis of β-keto thioesters and β-keto amides is reported. In refluxing water, the hydrolysis reactions of α-oxo ketene *N*,*S*-acetals in the presence of 1.0 equiv of dodecylbenzenesulfonic acid effectively afforded β-keto thioesters in excellent yield, while β-keto amides were successfully obtained in excellent yield when the hydrolysis reactions were carried out in the presence of 3.0 equiv of NaOH. The green approach to β-keto thioesters and β-keto amides avoids the use of harmful organic solvents, thiols and thiolacetates as well as amines, which could result in serious environmental and safety issues.

## Introduction

**Scheme 1:**
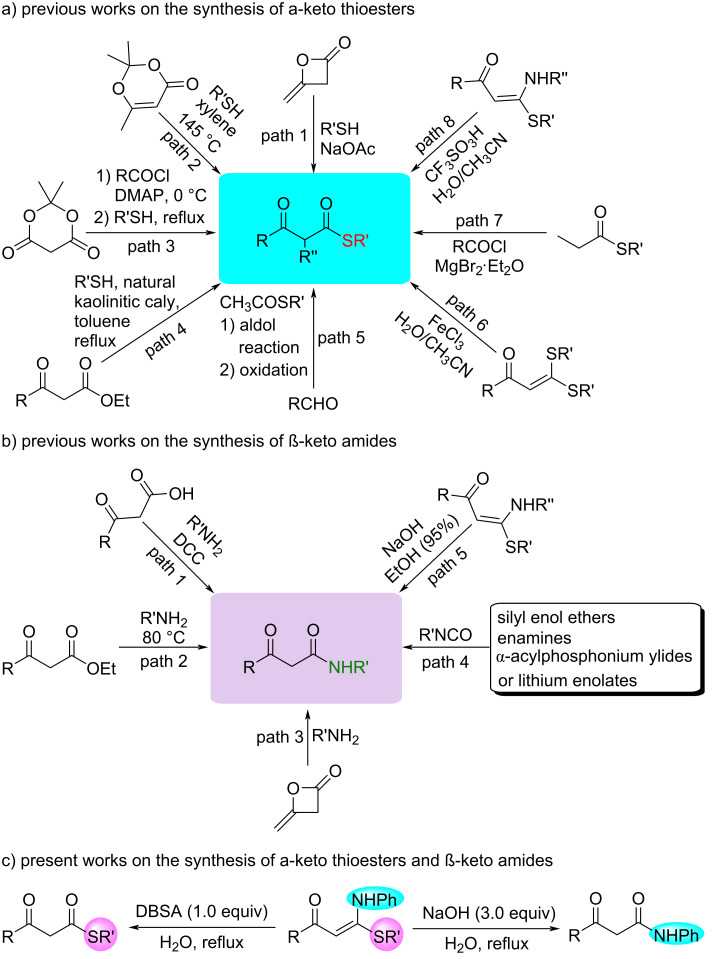
Synthesis of α-keto thioesters and β-keto amides.

In the past decades, the application of easily available and stable α-oxo ketene *N*,*S*-acetals as significant synthons has received more and more attention in organic synthesis due to their unique structural characters and multiple good reactivities [1–4]. Both β-keto thioesters [5–12] and β-keto amides [13–22] have served as useful synthetic intermediates for the synthesis of a range of potent natural products. Therefore, much effort has focused on their synthesis in the past decades [23–30]. These methods for the synthesis of β-keto thioesters include the mercaptolysis of diketene (Scheme 1a, path 1) [23], 2,2,6-trimethyl-4*H*-1,3-dioxin-4-one (Scheme 1a, path 2) [24], acylated Meldrum’s acids (Scheme 1a, path 3) [25] or β-keto esters (Scheme 1a, path 4) [26–27], the aldol reaction between aldehydes and *S*-ethyl acetothioate followed by oxidation with Dess–Martin periodinane (Scheme 1a, path 5) [28], the hydrolysis of α-oxo ketene dithioacetals (Scheme 1a, path 6) [29] and MgBr_2_·OEt_2_-catalyzed acylation of thioesters and acyl chlorides (Scheme 1a, path 7) [30]. For β-keto amides, they could be efficiently synthesized from the nucleophilic substitution reactions of amines with β-keto acids (Scheme 1b, path 1) [31–33], β-keto esters (Scheme 1b, path 2) [34] and the nucleophilic addition reactions of amines with diketenes (Scheme 1b, path 3) [35] as well as isocyanates with various nucleophilic reagents (Scheme 1b, path 4), such as silyl enol ethers [36], enamines [37], α-acylphosphonium ylides [38] and lithium enolates [39]. Recently, the hydrolysis of α-oxo ketene *N*,*S*-acetals was developed to prepare both β-keto thioesters and β-keto amides [40–41]. Li and co-authors achieved the synthesis of β-keto thioesters by CF_3_SO_3_H-promoted hydrolysis of α-oxo ketene *N*,*S*-acetals with an amino leaving group (Scheme 1a, path 8) [40]. Subsequently, using NaOH-promoted hydrolysis of α-oxo ketene *N*,*S*-acetals, we efficiently prepared β-keto amides (Scheme 1b, path 5) [41]. Despite great progress, all the reported reactions were performed in organic medium, such as CH_3_CN, CH_2_Cl_2_ and CF_3_CH_2_OH, which can result in serious environmental and safety problems. Therefore, the development of an environmentally compliant synthetic method for the preparation of β-keto thioesters and β-keto amides remains imperative.

Organic reactions in water are an important and exciting research topic of green chemistry because water as a solvent exhibits fascinating features, such as low cost, good environmental compatibility, nontoxicity and nonflammability. In addition, the use of water solvent can reduce the discharge of harmful organic solvents [42–50]. In the past 10 years, while expanding the synthesis and application of α-oxo ketene dithioacetal derivatives [51–64], we conducted research on their organic reactions in water and reported some good results, such as thioacetalization using ketene dithioacetals as odorless thiol equivalent [65], Friedel–Crafts alkylation of cyclic ketene dithioacetals with alcohols [66], the hydrolysis of chain α-oxo ketene dithioacetals [67] or β-ethylthio-β-indolyl-substituted α,β-unsaturated ketones [68], the cyclocondensation reaction of β-ethylthio-β-indolyl-substituted α,β-unsaturated ketones with hydrazines/hydroxylamine [69–71] and the tandem [5C + 1C/1N]-cycloaromatization of α-alkenoyl ketene dithioacetals and nitroethane/amines [72–73]. As part of our continuous research in this context, more recently we investigated the selective hydrolysis of α-oxo ketene *N*,*S*-acetals in water to gain an environmentally compliant synthetic method for β-keto thioesters and β-keto amides (Scheme 1c). Herein, we report our findings.

## Results and Discussion

At the outset of our studies, to optimize the reaction conditions for the selective synthesis of β-keto thioesters and β-keto amides, we explored the hydrolysis reaction of (*E*)-3-(ethylthio)-1-phenyl-3-(phenylamino)prop-2-en-1-one (**1a**, 0.25 mmol, 70.8 mg) in water under different conditions (Table 1). We initially tested the reaction in the presence of dodecylbenzenesulfonic acid (DBSA) in boiling water and found that the amount of DBSA has a dramatic influence on this reaction. Using 1.0 equiv of DBSA, the reaction efficiently gave the desired *S*-ethyl 3-oxo-3-phenylpropanethioate (**2a**) in 91% yield (Table 1, entry 1). However, increasing the amount of DBSA to 2.0 equiv, the yield of **2a** did not improve remarkably (Table 1, entry 2), and reducing the amount of DBSA resulted in a lower yield of **2a** (Table 1, entry 3). Additionally, when DBSA was replaced by other acids such as H_2_SO_4_ and CF_3_SO_3_H, the reaction showed poor effectiveness due to the poor solubility of **1a** in water (Table 1, entries 4 and 5). Most notably, **2a** was an inseparable mixture of keto and enol isomers, reaching a keto/enol ratio of 5:4 as determined by ^1^H NMR spectroscopy. Thus, the optimized reaction conditions for the synthesis of **2a** were determined to be 1.0 equiv of DBSA as catalyst and reflux temperature (conditions A). Subsequently, we turned our attention to the hydrolysis reaction in the presence of hydroxide for the preparation of 3-oxo-*N*,3-diphenylpropanamide (**3a**). Firstly, we chose NaOH to optimize the reaction conditions. Apparently, in the absence of a solubilizer, no reaction occurred due to poor solubility of **1a** in boiling water (Table 1, entry 6). Macrogol 400 (PEG-400) is emerging as an environmentally friendly nonionic solubilizer due to its unique merits, such as nontoxicity, inexpensiveness, nonflammability, low volatility and good water solubility, which are consistent with the concept of green chemistry [74–75]. Therefore, we tested the reaction in the presence of PEG-400 (Table 1, entries 7–9). It was found that the reaction uniquely produced **3a** in 48% yield when using 3.0 equiv of PEG-400 as solubilizer (Table 1, entry 8), and further increasing the PEG-400 loading could not remarkably improve the yield of **3a** (Table 1, entry 9). With this in mind, we selected 3.0 equiv of PEG-400 as solubilizer in our study. Next, we examined the influence of the amount of NaOH on the reaction (Table 1, entries 10–12). The reaction obviously showed dependence on the amount of NaOH, and **3a** was obtained in 90% yield when the reaction ran for 24 h in the presence of 3.0 equiv of NaOH (Table 1, entry 11). However, when lowering the reaction temperature to 90 °C, the reaction efficiency significantly decreased (Table 1, entry 13). Alike **2a**, the keto isomer of **3a** was the dominant isomer, with a keto/enol ratio of 3:1. Then, we tested the effects of different bases on the reaction and found that the strong bases NaOH and KOH gave **3a** in high yield (Table 1, entries 11 and 14), while the reaction afforded **3a** in low yield in the presence of weak bases such as Na_2_CO_3_ and Et_3_N (Table 1, entries 15 and 16). Accordingly, the optimal reaction conditions for the synthesis of **3a** were 3.0 equiv of NaOH as catalyst and reflux temperature (conditions B).

**Table 1:** Optimization of the reaction conditions.

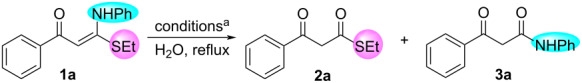					

entry	catalyst (equiv)	PEG-400 (equiv)	time (h)	yield (%)^b^	

				**2a**	**3a**

1	DBSA (1.0)	—	5	91	5
2	DBSA (2.0)	—	2	89	3
3	DBSA (0.5)	—	8	70	24
4	H_2_SO_4_ (1.0)	—	12	61	18 (12)^c^
5	CF_3_SO_3_H (1.0)	—	12	56	15 (22)^c^
6	NaOH (1.0)	—	24	0	0 (94)^c^
7	NaOH (1.0)	2.0	24	0	30 (60)^c^
8	NaOH (1.0)	3.0	24	0	48 (47)^c^
9	NaOH (1.0)	4.0	24	0	49 (42)^c^
10	NaOH (2.0)	3.0	24	0	72 (21)^c^
11	NaOH (3.0)	3.0	24	0	90
12	NaOH (4.0)	3.0	22	0	89
13^d^	NaOH (3.0)	3.0	24	0	74 (18)^c^
14	KOH (3.0)	3.0	24	0	88
15	Na_2_CO_3_ (3.0)	3.0	24	0	31 (60)^c^
16	Et_3_N (3.0)	3.0	24	0	20 (72)^c^

^a^Conditions: **1a** (0.25 mmol, 70.8 mg), H_2_O (1 mL), in air. ^b^Isolated yield. ^c^Recovery rate of **1a**. ^d^Reaction at 90 °C.

**Scheme 2:**
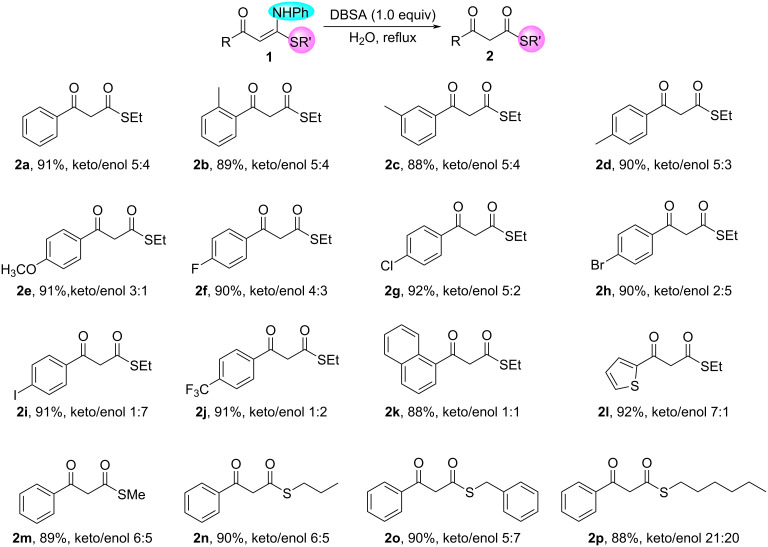
Synthesis of β-keto thioesters **2**. Reaction conditions A: **1** (0.25 mmol), DBSA (87.9 mg, 0.25 mmol), H_2_O (1 mL), reflux, 5 h. Isolated yield is stated. Keto/enol ratio of **2** was determined by ^1^H NMR spectroscopy.

With the optimal reaction conditions in hand, we next examined the scope of the two hydrolysis reactions (Scheme 2). Initially, the hydrolysis reaction for the synthesis of β-keto thioesters **2** was investigated under conditions A, and the results are shown in Scheme 2. (*E*)-3-(Ethylthio)-1-aryl-3-(phenylamino)prop-2-en-1-ones **1a**–**l** were smoothly hydrolyzed to produce a series of *S*-ethyl 3-oxo-3-arylpropanethioates **2a**–**l** in excellent yield, wherein the electronic effects of electron-donating and electron-withdrawing groups on the aromatic ring adjacent to the carbonyl group did not impact the formation of the products **2a**–**l**. A variety of valuable functional groups on the benzene ring of **1b**–**j**, such as methyl, methoxy, trifluoromethyl, and halogen atoms (F, Cl, Br, I), were well compatible with reaction conditions A. Most notably, unlike work by Li et al. [40], *S*-ethyl 3-oxo-3-(4-(trifluoromethyl)phenyl)propanethioate (**2j**) could be obtained in 91% yield. In a similar fashion, *S*-alkyl 3-oxo-3-phenylpropanethioates **2m**–**p** were also efficiently prepared in excellent yield from the hydrolysis reaction of (*E*)-3-(alkylthio)-1-aryl-3-(phenylamino)prop-2-en-1-ones **1m**–**p** under conditions A.

**Scheme 3:**
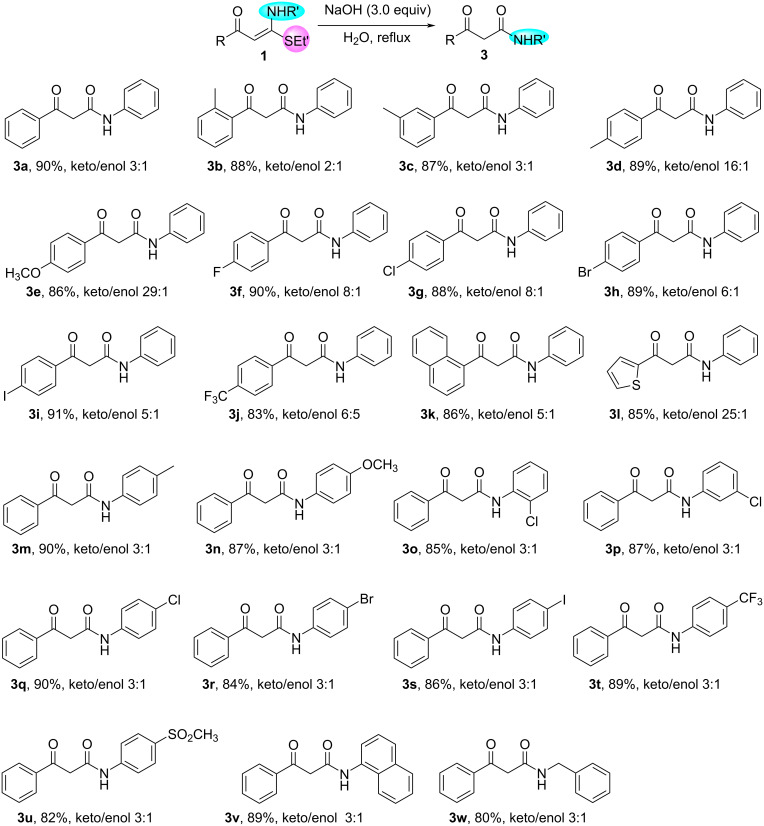
Synthesis of β-keto amides **3**. Reaction conditions B: **1** (0.25 mmol), NaOH (0.75 mmol, 30 mg), H_2_O (1 mL), reflux, 24 h. Isolated yield is stated. Keto/enol ratio of **3** was determined by ^1^H NMR spectroscopy.

Next, the generality of the synthesis of β-keto amides **3** under conditions B was investigated (Scheme 3). 3-Oxo-*N-*phenyl-3-arylpropanamides **3a**–**l** and 3-oxo-*N-*aryl-3-phenylpropanamides **3m**–**v** could be produced in excellent yield from the hydrolysis of α-oxo ketene *N*,*S*-acetals under conditions B. The results showed that electron-donating as well as electron-withdrawing substituents on the two phenyl rings in compounds **1**, such as methyl, methoxy, halogen atoms (F, Cl, Br, I), CF_3_ and SO_2_CH_3_, were well tolerated, and their electronic effects insignificantly impacted the formation of **3**. Similarly, *N*-benzyl-3-oxo-3-phenylpropanamide (**3w**) could also be obtained in 80% yield when the hydrolysis reaction of (*E*)-3-(benzylamino)-3-(ethylthio)-1-phenylprop-2-en-1-one (**1aa**) was carried out under conditions B.

**Scheme 4:**
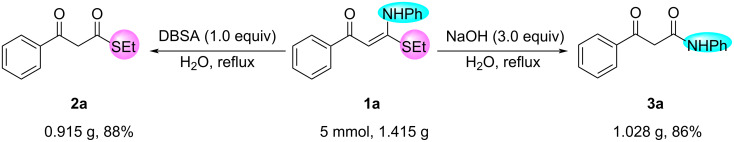
Gram-scale hydrolysis reactions of **1a**.

Furthermore, to explore the synthetic practicality of the two chemical processes, the synthesis of **2a** and **3a** on a gram scale was tested using the hydrolysis reaction of **1a** under conditions A and B, respectively. When the hydrolysis reactions of **1a** were performed on a 5 mmol scale, 0.915 g of **2a** and 1.028 g of **3a** were obtained in 88% and 86% yield, respectively (Scheme 4).

Next, the EcoScale value of the hydrolysis reactions of **1a** under conditions A and B was calculated [76] to evaluate the green metrics of the two aqueous reactions (Table 2). It was found that the two procedures showed EcoScale values of 82.5 and 77.0, respectively, which indicated that the two procedures exhibited good environmental friendliness.

**Table 2:** EcoScale calculations and results for the synthesis of **2a** and **3a** [75].

synthesis of **2a**		

parameter	parameter details	penalty points

yield	91%	4.5
price of reaction components to obtain 10 mmol of product	**1a**	0
	DBSA	0
safety	DBSA	0
technical setup	common setup	0
temperature, time	heating, >1 h	3
workup and purification	classical chromatography	10
EcoScale score		82.5

synthesis of **3a**		

parameter	parameter details	penalty points

yield	90%	5
price of reaction components to obtain 10 mmol of product	**1a**	0
	PEG-400	0
	NaOH	0
safety	PEG-400	0
	NaOH	5 (dangerous for environment)
technical setup	common setup	0
temperature, time	heating, >1 h	3
workup and purification	classical chromatography	10
EcoScale score		77.0

**Scheme 5:**
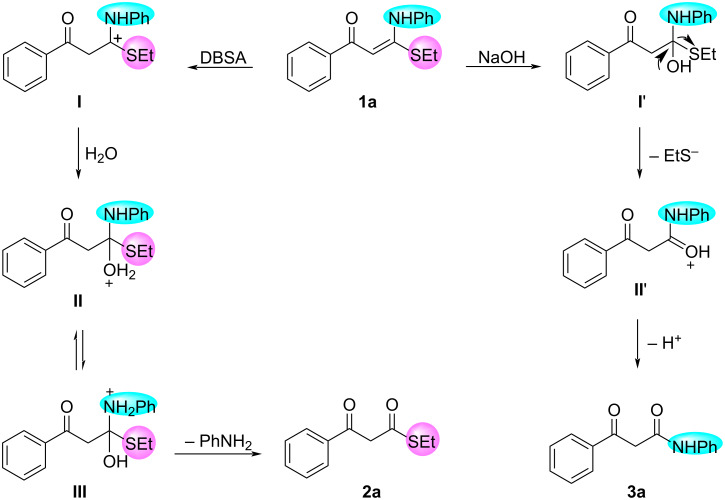
Proposed mechanism for formation of β-keto thioesters **2** and β-keto amides **3**.

Based on the results above and on literature precedents [40–41], a plausible mechanistic pathway for the formation of **2** and **3** is shown in Scheme 5 (with the reaction of **1a** as an example). In the presence of DBSA, the protonation of **1a** results in the carbocation intermediate **I**. Then, the nucleophilic attack of H_2_O at the carbocation of **I** produces intermediate **II**, which converts into intermediate **III** through a deprotonation–protonation process. Finally, the elimination of PhNH_2_ from intermediate **III** occurs to afford the desired product **2a**. In the presence of NaOH, the Michael addition between **1a** and base initially occurs to form adduct **I'**, which is then transformed into intermediate **II'** by elimination of ethanethiolate. Subsequently, β-keto amide **3a** is obtained when **II'** releases H^+^.

## Conclusion

In summary, we have successfully developed an environmentally friendly method for the selective aqueous synthesis of β-keto thioesters and β-keto amides by simply changing reaction conditions. These features, including a good substrate scope, excellent yield and selectivity and ease of scale-up, rendered the green hydrolysis reaction very environment-friendly, practical, and attractive.

## Experimental

^1^H and ^13^C{^1^H} NMR spectra were recorded on a Bruker DRX-600 spectrometer and all chemical shift values are referenced to TMS (δ = 0.00 ppm for ^1^H) and CDCl_3_ (δ = 77.16 ppm for ^13^C). HRMS analysis was achieved with a Bruck microTof using the ESI method. All melting points are uncorrected. Analytical TLC plates (Sigma-Aldrich silica gel 60 F200) were analyzed under UV light (254 nm). Chromatographic purifications were performed on SDZF silica gel 160.

The starting α-oxo ketene *N*,*S*-acetals **1** are known compounds [40–41] and easily prepared according to References [40,58].

### Typical procedure for the preparation of β-keto thioesters **2** (**2a** as an example)

A mixture of (*E*)-3-(ethylthio)-1-phenyl-3-(phenylamino)prop-2-en-1-one (**1a**, 70.8 mg, 0.25 mmol) and DBSA (87.9 mg, 0.25 mmol) in water (2 mL) was stirred at reflux in an oil bath under air for 5 h until **1a** was completely consumed, as confirmed by TLC monitoring. Then, the pH value of the reaction mixture was adjusted to neutral using a saturated NaHCO_3_ solution, and the reaction mixture was extracted with CH_2_Cl_2_ (3 × 20 mL). The organic solution was dried with anhydrous Na_2_SO_4_, and the crude product was purified by column chromatography on 300–400 mesh silica gel (petroleum ether (60–90 °C)/ethyl acetate 60:1, v/v) to give **2a** (47.3 mg, 91%).

### Typical procedure for the preparation of β-keto amides **3** (**3a** as an example)

A mixture of (*E*)-3-(ethylthio)-1-phenyl-3-(phenylamino)prop-2-en-1-one (**1a**, 70.8 mg, 0.25 mmol), NaOH (30 mg, 0.75 mmol) and PEG-400 (0.236 mL, 0.75 mmol) in water (2 mL) was stirred at reflux in an oil bath under air for 24 h until **1a** was completely consumed, as confirmed by TLC monitoring. Then, the pH value of the reaction mixture was adjusted to neutral using a 10% CH_3_COOH solution, and the reaction mixture was extracted with CH_2_Cl_2_ (3 × 20 mL). The organic solution was dried with anhydrous Na_2_SO_4_, and the crude product was purified by column chromatography on 30–400 mesh silica gel (petroleum ether (60–90 °C)/ethyl acetate 20:1, v/v) to give **3a** (53.8 mg, 90%).

## Supporting Information

File 1 Analytic data and copies of ^1^H and ^13^C NMR spectra of compounds **2** and **3**.

## Data Availability

All data that supports the findings of this study is available in the published article and/or the supporting information to this article.
