# Supplementary material for: Selective hydrolysis of α-oxo ketene N,S-acetals in water: switchable aqueous synthesis of β-keto thioesters and β-keto amides
Source: Beilstein J Org Chem. 2024 Sep 3;20:2225–33. doi: 10.3762/bjoc.20.190 (PMC11403796; doi:10.3762/bjoc.20.190)
Supplement: File 1 — Analytic data and copies of 1H and 13C NMR spectra of compounds 2 and 3. [file Beilstein_J_Org_Chem-20-2225-s001.pdf]

BEILSTEIN JOURNAL OF ORGANIC CHEMISTRY

Supporting Information

for

Selective hydrolysis of α-oxo ketene *N*,*S*-acetals in water: switchable aqueous synthesis of β-keto thioesters and β-keto amides

Haifeng Yu, Wanting Zhang, Xuejing Cui, Zida Liu, Xifu Zhang and Xiaobo Zhao

Beilstein J. Org. Chem. **2024,** *20,* 2225–2233. doi:10.3762/bjoc.20.190

Analytic data and copies of ^{1}H and ^{13}C NMR spectra of compounds 2 and 3

License and Terms: This is a supporting information file under the terms of the Creative Commons Attribution License (https://creativecommons.org/licenses/by/4.0). Please note that the reuse, redistribution and reproduction in particular requires that the author(s) and source are credited and that individual graphics may be subject to special legal provisions.
The license is subject to the *Beilstein Journal of Organic Chemistry* terms and conditions: (https://www.beilstein-journals.org/bjoc/terms)

1. Spectroscopic Data of compound **2** S1

2. Spectroscopic Data of compound **3** S7

3. References S16

4. Copies of ^{1}H NMR and ^{13}C NMR spectra of compound **2** S17

5. Copies of ^{1}H NMR and ^{13}C NMR spectra of compound **3** S33

1. Spectroscopic Data of compound 2

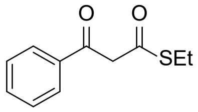

***S*-Ethyl 3-oxo-3-phenylpropanethioate (2a)** [1,2]: colorless liquid. Keto/enol 5:4. Yield: 91% (47.3 mg), ^{1}H NMR ($CDCl_3$, 400 MHz) δ for keto-**2a**: 7.97 (dd, J_1 = 8.6 Hz, J_2 = 1.4 Hz, 2 H), 7.60 (t, *J* = 7.4 Hz, 1 H), 7.49 (t, *J* = 8.0 Hz, 2 H), 4.21 (s, 2 H), 2.97-2.91 (m, 2 H), 1.27 (t, *J* = 7.4 Hz, 3 H); for enol-**2a**: 13.2 (s, 1 H), 7.79 (dd, J_1 = 8.6 Hz, J_2 = 1.5 Hz, 2 H), 7.49 (t, *J* = 8.0 Hz, 1 H), 7.42 (t, *J* = 8.2 Hz, 2 H), 6.09 (s, 1H), 3.03-2.98 (m, 2 H), 1.32 (t, *J* = 7.4 Hz, 3 H). ^{13}C NMR ($CDCl_3$, 100 MHz) δ for keto-**2a**: 192.4, 192.2, 134.0, 131.8, 129.0 (2 C), 126.5 (2 C), 54.1, 24.2, 14.6; for enol-**2a**: 195.2, 168.9, 136.2, 133.3, 128.9 (2 C), 128.8 (2 C), 97.4, 23.0, 15.1.

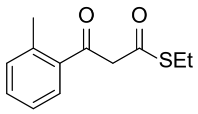

***S*-Ethyl 3-oxo-3-(*o*-tolyl)propanethioate (2b)** [2]: colorless liquid. Keto/enol 5:4. Yield: 89% (49.4 mg). ^{1}H NMR ($CDCl_3$, 400 MHz) δ for keto-**2b**: 7.69 (d, *J* = 7.8 Hz, 1 H), 7.40 (t, *J* = 7.8 Hz, 2 H), 7.34-7.28 (m, 1 H), 4.16 (s, 2 H), 2.95-2.90 (m, 2 H), 2.54 (s, 3 H), 1.25 (t, *J* = 7.4 Hz, 3 H); for enol-**2b**: 13.1 (s, 1 H), 7.34-7.19 (m, 4 H), 5.71 (s, 1H), 3.03-2.97 (m, 2 H), 2.47 (s, 3 H), 1.34 (t, *J* = 7.4 Hz, 3 H). ^{13}C NMR ($CDCl_3$, 100 MHz) δ for keto-**2b**: 192.5, 192.2, 139.7, 136.4, 132.4, 131.3, 129.6, 125.9, 56.4, 24.1, 21.7, 14.6; for enol-**2b**: 195.1, 172.4, 137.0, 133.9, 132.3, 130.5, 128.6, 125.9, 101.6, 23.0, 20.7, 15.0.

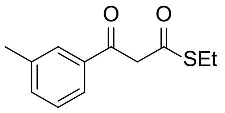

***S*-Ethyl 3-oxo-3-(*m*-tolyl)propanethioate (2c)** [2]: colorless liquid. Keto/enol 5:4. Yield: 88% (48.8 mg). ^{1}H NMR ($CDCl_3$, 400 MHz) δ for keto-**2c**: 7.77 (s, 1 H), 7.44 (d, *J* = 7.8 Hz, 1 H), 7.40 (d, *J* = 7.5 Hz, 1 H), 7.34-7.29 (m, 1 H), 4.19 (s, 2 H), 2.96-2.91 (m, 2 H), 2.41 (s, 3 H), 1.26 (t, *J* = 7.4 Hz, 3 H); for enol-**2c**: 13.2 (s, 1 H), 7.60 (s, 1 H), 7.58 (d, *J* = 7.8 Hz, 1 H), 7.37 (d, *J* = 7.5 Hz, 1 H), 7.34-7.29 (m, 1 H), 6.07 (s, 1H), 3.03-2.97 (m, 2 H), 2.39 (s, 3 H), 1.32 (t, *J* = 7.4 Hz, 3 H). ^{13}C NMR ($CDCl_3$, 100 MHz) δ for keto-**2c**: 192.5, 192.3, 138.8, 134.7, 132.6, 129.4, 128.8, 126.2, 54.1, 24.1, 21.5, 14.6; for enol-**2c**: 195.1, 169.1, 138.5, 136.2, 133.0, 129.4, 128.6, 127.1, 97.3, 23.0, 21.5, 15.1.

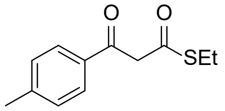

***S*-Ethyl 3-oxo-3-(*p*-tolyl)propanethioate (2d)** [1,2]: colorless liquid. Keto/enol 5:3. Yield: 90% (50.0 mg). ^{1}H NMR ($CDCl_3$, 400 MHz) δ for keto-**2d**: 7.87 (d, *J* = 8.0 Hz, 2 H), 7.28 (d, *J* = 8.3 Hz, 2 H), 4.18 (s, 2 H), 2.96-2.90 (m, 2 H), 2.42 (s, 3 H), 1.26 (t, *J* = 7.4 Hz, 3 H); for enol-**2d**: 13.2 (s, 1 H), 7.68 (d, *J* = 8.3 Hz, 2 H), 7.22 (d, *J* = 8.1 Hz, 2 H), 6.06 (s, 1 H), 3.02-2.97 (m, 2 H), 2.39 (s, 3 H), 1.33 (t, *J* = 7.4 Hz, 3 H). ^{13}C NMR ($CDCl_3$, 100 MHz) δ for keto-**2d**: 195.0, 191.7, 145.0, 133.8, 129.6 (2 C), 129.1 (2 C), 54.2, 24.2, 21.9, 14.6; for enol-**2d**: 192.5, 169.2, 142.4, 130.3, 129.5 (2 C), 126.5 (2 C), 96.8, 23.0, 21.7, 15.1.

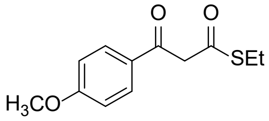

***S*-Ethyl 3-(4-methoxyphenyl)-3-oxopropanethioate (2e)** [1,2]: colorless liquid. Keto/enol 3:1. Yield: 91% (54.1 mg). ^{1}H NMR ($CDCl_3$, 400 MHz) δ for keto-**2e**: 7.95 (d, *J* = 8.9 Hz, 2 H), 6.94 (d, *J* = 8.5 Hz, 2 H), 4.16 (s, 2 H), 3.87 (s, 3 H), 2.95-2.90 (m, 2 H), 1.26 (t, *J* = 7.4 Hz, 3 H); for enol-**2e**: 13.3 (s, 1 H), 7.75 (d, *J* = 8.9 Hz, 2 H), 6.92 (d, *J* = 8.5 Hz, 2 H), 6.01 (s, 1 H), 3.85 (s, 3 H),

3.02-2.97 (m, 2 H), 1.33 (t, *J* = 7.4 Hz, 3 H). ^{13}C NMR ($CDCl_3$, 100 MHz) δ for keto-**2e**: 192.6, 190.5, 164.2, 131.4 (2 C), 128.3, 114.1 (2 C), 55.6, 54.0, 24.1, 14.5; for enol-**2e**: 194.7, 169.0, 162.6, 129.2 (2 C), 125.2, 114.1(2 C), 96.0, 55.5, 23.0, 15.1.

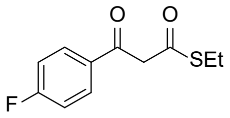

***S*-Ethyl 3-(4-fluorophenyl)-3-oxopropanethioate (2f)**: colorless liquid. Keto/enol 4:3. Yield: 90% (50.9 mg). ^{1}H NMR ($CDCl_3$, 400 MHz) δ for keto-**2f**: 8.02-7.98 (m, 2 H), 7.15 (t, *J* = 8.6 Hz, 2 H), 4.18 (s, 2 H), 2.96-2.90 (m, 2 H), 1.26 (t, *J* = 7.4 Hz, 3 H); for enol-**2f**: 13.3 (s, 1 H), 7.81-7.77 (m, 2 H), 7.10 (t, *J* = 8.6 Hz, 2 H), 6.02 (s, 1 H), 3.02-2.97 (m, 2 H), 1.33 (t, *J* = 7.4 Hz, 3 H). ^{13}C NMR ($CDCl_3$, 100 MHz) δ for keto-**2f**: 192.2, 190.5, 166.3 (d, *J* = 254.7 Hz), 132.6 (d, *J* = 2.9 Hz), 131.7 (d, *J* = 9.6 Hz, 2 C), 116.1 (d, *J* = 21.9 Hz, 2 C), 54.2, 24.2, 14.5; for enol-**2f**: 195.2, 167.8, 165.0 (d, *J* = 21.1 Hz), 129.2 (d, *J* = 3.0 Hz), 128.8 (d, *J* = 8.8 Hz, 2 C), 115.9 (d, *J* = 21.9 Hz, 2 C), 97.1, 23.1, 15.0. HRMS Cacld for $C_{11}H_{12}FO_2S^+$: ($[M+H]^+$) 227.0537; Found: 227.0539.

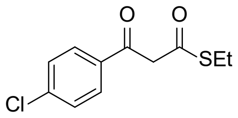

***S*-Ethyl 3-(4-chlorophenyl)-3-oxopropanethioate (2g)** [1,2]: colorless liquid. Keto/ enol = 5:2. Yield: 92% (55.7 mg). ^{1}H NMR (DMSO-d_6, 400 MHz) δ for keto-**2g**: 7.96 (d, *J* = 8.6 Hz, 2 H), 7.61 (d, *J* = 8.6 Hz, 2 H), 4.46 (s, 2 H), 2.88-2.83 (m, 2 H), 1.16 (t, *J* = 7.4 Hz, 3 H); for enol-**2g**: 13.2 (s, 1 H), 7.90 (d, *J* = 8.7 Hz, 2 H), 7.53 (d, *J* = 8.6 Hz, 2 H), 6.47 (s, 1H), 2.99-2.93 (m, 2 H), 1.24 (t, *J* = 7.4 Hz, 3 H). ^{13}C NMR (DMSO-d_6, 100 MHz) for keto-**2g**: 192.6, 192.0, 138.8, 134.5, 130.4 (2 C), 128.9 (2 C), 53.5, 23.2, 14.5; for enol-**2g**: 194.9, 166.2, 135.7, 131.1, 128.9 (2 C), 128.2 (2 C), 97.5, 22.5, 14.8.

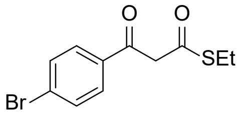

***S*-Ethyl 3-(4-bromophenyl)-3-oxopropanethioate (2h)** [1,2]: slight yellow oil. Keto/ enol = 2:5. Yield: 90% (64.4 mg). ^{1}H NMR ($CDCl_3$, 400 MHz) δ for keto-**2h**: 7.75 (d, *J* = 8.6 Hz, 2 H), 7.55 (d, *J* = 8.6 Hz, 2 H), 4.10 (s, 2 H), 2.89-2.83 (m, 2 H), 1.19 (t, *J* = 7.4 Hz, 3 H); for enol-**2h**: 13.1 (s, 1 H), 7.57 (d, *J* = 7.9 Hz, 2 H), 7.48 (d, *J* = 8.6 Hz, 2 H), 5.98 (s, 1 H), 2.96-2.90 (m, 2 H), 1.26 (t, *J* = 7.4 Hz, 3 H). ^{13}C NMR ($CDCl_3$, 100 MHz) δ for keto-**2h**: 192.1, 191.0, 134.9, 132.3 (2 C), 130.5 (2 C), 129.4, 54.1, 23.2, 14.5; for enol-**2h**: 195.3, 167.6, 132.0 (2 C), 128.0 (3 C), 126.3, 97.5, 23.2, 15.0.

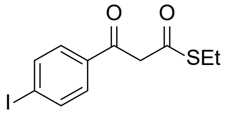

***S*-Ethyl 3-(4-iodophenyl)-3-oxopropanethioate (2i)**: colorless crystal, m.p. 46-47 °C. Keto/enol 1:7. Yield: 91% (76.0 mg). ^{1}H NMR ($CDCl_3$, 400 MHz) δ for keto-**2i**: 7.85 (d, *J* = 8.6 Hz, 2 H), 7.67 (d, *J* = 8.6 Hz, 2 H), 4.16 (s, 2 H), 2.96-2.91 (m, 2 H), 1.25 (t, *J* = 7.4 Hz, 3 H); for enol-**2i**: 13.2 (s, 1 H), 7.77 (d, *J* = 8.5 Hz, 2 H), 7.50 (d, *J* = 8.6 Hz, 2 H), 6.06 (s, 1H), 3.03-2.97 (m, 2 H), 1.33 (t, *J* = 7.4 Hz, 3 H). ^{13}C NMR ($CDCl_3$, 100 MHz) δ for keto-**2i**: 192.1, 191.5, 138.3 (2 C), 135.4, 130.3 (2 C), 102.3, 54.1, 24.3, 14.6; for enol-**2i**: 195.3, 167.8, 138.0 (2 C), 132.6, 128.0 (2 C), 98.6, 97.5, 23.2, 15.0. HRMS Cacld for $C_{11}H_{12}IO_2S^+$: ($[M+H]^+$) 334.9597; Found: 334.9598.

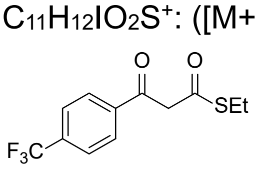

***S*-Ethyl 3-oxo-3-(4-(trifluoromethyl)phenyl)propanethioate (2j)** [1]: colorless crystal, m.p. 29-30 °C. Keto/enol 1:2. Yield: 91% (62.8 mg). ^{1}H NMR ($CDCl_3$, 400 MHz) δ for keto-**2j**: 8.08 (d, *J* = 8.2 Hz, 2 H), 7.75 (d, *J* = 8.3 Hz, 2 H), 4.23 (s, 2 H), 2.97-2.92 (m, 2 H), 1.27 (t, *J* = 7.4 Hz, 3 H); for enol-**2j**: 13.2 (s, 1 H), 7.89 (d, *J* = 8.2 Hz, 2 H), 7.68 (d, *J* = 8.3 Hz, 2 H), 6.11 (s, 1 H), 3.05-2.99 (m, 2 H), 1.34 (t, *J* = 7.4 Hz, 3 H). ^{13}C NMR ($CDCl_3$, 100 MHz) δ for

keto-**2j**: 191.9, 191.3, 138.7, 135.25 (q, *J* = 21.6 Hz), 129.3 (2 C), 126.00 (q, *J* = 3.6 Hz), 123.60 (q, *J* = 271.0 Hz), 54.3, 24.3, 14.5; for enol-**2j**: 195.6, 167.8, 136.6, 133.17(q, *J* = 32.5 Hz), 126.8 (2 C), 125.74(q, *J* = 3.8 Hz), 123.85 (q, *J* = 270.7 Hz), 98.6, 23.2, 15.0.

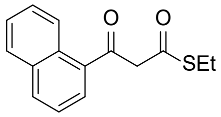

***S*-Ethyl 3-(naphthalen-1-yl)-3-oxopropanethioate (2k)**: colorless liquid. Keto/enol 1:1. Yield: 88% (56.8 mg). ^{1}H NMR ($CDCl_3$, 400 MHz) δ for keto-**2k**: 8.78 (d, *J* = 8.3 Hz, 1 H), 7.96 (d, *J* = 8.4 Hz, 1 H), 7.88 (d, *J* = 8.4 Hz, 2 H), 7.63 (t, *J* = 8.4 Hz, 1 H), 7.58-7.46 (m, 2 H), 4.33 (s, 2 H), 2.96-2.91 (m, 2 H), 1.25 (t, *J* = 7.4 Hz, 3 H); for enol-**2k**: 13.4 (s, 1 H), 8.38 (d, *J* = 8.2 Hz, 1 H), 8.03 (d, *J* = 8.2 Hz, 1 H), 7.93 (d, *J* = 8.4 Hz, 1 H), 7.67 (t, *J* = 7.2 Hz, 1 H), 7.58-7.46 (m, 3 H), 6.06 (s, 1H), 3.03-2.97 (m, 2 H), 1.33 (t, *J* = 7.4 Hz, 3 H). ^{13}C NMR ($CDCl_3$, 100 MHz) δ for keto-**2k**: 195.1, 192.5, 134.0, 133.8, 132.0, 130.5, 129.5, 128.6, 127.1, 126.8, 126.0, 125.0, 56.7, 24.2, 14.6; for enol-**2k**: 195.4, 171.6, 134.0, 131.1, 130.4, 128.6 (2 C), 127.1 (2 C), 126.4, 125.6, 124.3, 102.6, 23.1, 15.1. HRMS Cacld for $C_{15}H_{15}O_2S^+$: ($[M+H]^+$) 259.0787; Found: 259.0789.

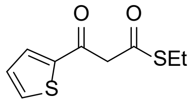

***S*-Ethyl 3-oxo-3-(thiophen-2-yl)propanethioate (2l)** [1,2]: colorless liquid. Keto/enol 7:1. Yield: 92% (49.2 mg). ^{1}H NMR ($CDCl_3$, 400 MHz) δ for keto-**2l**: 7.78 (dd, J_1 = 7.8 Hz, J_2 = 1.1 Hz, 1 H), 7.71 (dd, J_1 = 7.7 Hz, J_2 = 1.1 Hz, 1 H), 7.16 (dd, J_1 = 4.9 Hz, J_2 = 3.8 Hz, 1 H), 4.13 (s, 2 H), 2.97-2.91 (m, 2 H), 1.27 (t, *J* = 7.4 Hz, 3 H); for enol-**2l**: 13.2 (s, 1 H), 7.60 (dd, J_1 = 7.8 Hz, J_2 = 1.1 Hz, 1 H), 7.50 (dd, J_1 = 7.7 Hz, J_2 = 1.1 Hz, 1 H), 7.11 (dd, J_1 = 4.9 Hz, J_2 = 3.8 Hz, 1 H), 5.97 (s, 1H), 3.02-2.98 (m, 2 H), 1.33 (t, *J* = 7.4 Hz, 3 H). ^{13}C NMR ($CDCl_3$, 100 MHz) δ for keto-**2l**: 191.8, 184.4, 143.5, 135.3, 133.9, 128.5, 54.9, 24.3, 14.5; for enol-**2l**: 194.8, 163.9, 137.0, 130.3, 128.8, 128.3, 96.5, 23.1, 15.1.

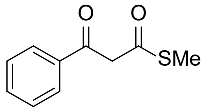

***S*-Methyl 3-oxo-3-phenylpropanethioate (2m)** [2]: slight yellow oil. Keto:enol = 6:5. Yield: 89% (43.2 mg). ^{1}H NMR (600 MHz, $CDCl_3$) δ for keto-**2m**: 7.95 (d, *J*=8.3Hz, 2 H), 7.57 (t, 1 H), 7.45 (t, 2 H), 4.22 (s, 2 H), 2.33 (s, 3 H); for enol-**2m**: 13.2 (br, 1 H), 7.77 (d, *J*=8.3Hz, 2 H), 7.45 (t, 1 H), 7.40 (t, 2 H), 6.10 (s, 1 H), 2.38 (s, 3 H). ^{13}C NMR (150 MHz, $CDCl_3$) δ for keto-**2m**: 192.5, 191.9, 135.9, 133.8, 128.8 (2 C), 126.4 (2 C), 53.7, 12.2; for keto-**2m**: 195.2, 168.6, 132.8, 131.7, 128.8 (2 C), 128.6 (2 C), 97.1, 11.1.

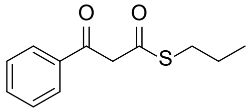

***S*-Propyl 3-oxo-3-phenylpropanethioate (2n)** [2]: slight yellow oil. Keto:enol = 6:5. Yield: 90% (50.0 mg). ^{1}H NMR (600 MHz, $CDCl_3$) δ for keto-**2n**: 7.96 (d, *J*=7.6 Hz, 2 H), 7.59 (t, 1 H), 7.48 (t, 2 H), 4.22 (s, 2 H), 2.91 (t, 2 H), 1.62-1.60 (m, 2 H), 0.96 (t, 3 H); for enol-**2n**: 13.2 (br, 1 H), 7.78 7.96 (d, *J*=7.5 Hz, 2 H), 7.48 (t, 1 H), 7.42 (t, 2 H), 6.09 (s, 1 H), 2.97 (t, 2 H), 1.69-1.67 (m, 2 H), 1.02 (t, 3 H).^{13}C NMR (150 MHz, $CDCl_3$) δ for keto-**2n**: 192.5, 192.20, 136.1, 133.9, 128.9 (2 C), 126.5 (2 C), 54.1, 31.6, 22.8, 13.4; for enol-**2n**: 195.2, 168.8, 133.0, 131.7, 128.9 (2 C), 128.7(2 C), 97.4, 30.5, 23.2, 13.5.

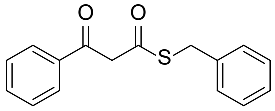

***S*-Benzyl 3-oxo-3-phenylpropanethioate (2o)** [2]: slight yellow oil, Keto:enol=5:7. Yield: 90% (60.8 mg). ^{1}H NMR (600 MHz, $CDCl_3$) δ for keto-**2o**: 7.94 (d, *J*=8.2 Hz, 2 H), 7.58 (t, 1 H), 7.47 (t, 2 H), 7.35 (d, *J*=8.2 Hz, 2 H), 7.32-7.30 (m, 3 H), 4.22 (s, 2 H), 4.17 (s, 2 H); for enol-**2o**: 13.2 (s, 1 H), 7.77 (d, *J*=8.2 Hz, 2 H), 7.47 (t, 1 H), 7.41 (t, 2 H), 7.28 (d, *J*=8.2 Hz, 2 H), 7.27 (t, 1 H), 7.24 (t, 2 H), 6.08 (s, 1 H), 4.24 (s, 2 H). ^{13}C NMR (150 MHz, $CDCl_3$) δ for keto-**2o**:194.1, 191.7, 136.9, 136.0, 132.9, 129.0 (4 C), 128.9 (2 C), 127.6,

126,5 (2 C), 53.7, 34.0; for enol-**2o**: 191.9, 169.3, 137.6, 134.0, 131.9, 128.8 (6 C), 127.4, 126,5 (2 C), 96.9, 32.7. HRMS Cacld for $C_{16}H_{15}O_2S^+$: ([M+H]$^+$) 271.0787; Found: 271.0772.

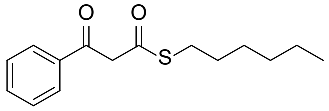

***S*-Hexyl 3-oxo-3-phenylpropanethioate (2p)** [2]: slight yellow oil. Keto:enol=21:20. Yield: 88% (58.1 mg). ^{1}H NMR (600 MHz, $CDCl_3$) δ for keto-**2p**: 7.96 (d, *J*=8.3 Hz, 2 H), 7.58 (t, 1 H), 7.47 (t, 2 H), 4.21 (s, 2 H), 2.92 (t, 2 H), 1.58-1.55 (m, 2 H), 1.41-1.39 (m, 2 H), 1.35-1.31 (m, 2 H), 1.28-1.25 (m, 2 H), 0.87 (t, 3 H); for enol-**2p**: 13.2 (br, 1 H), 7.78 (d, *J*=8.3 Hz, 2 H), 7.47 (t, 1 H), 7.41(t, 2 H), 6.09 (s, 1 H), 2.99 (t, 2 H), 1.67-1.62 (m, 2 H), 1.35-1.32 (m, 2 H), 1.35-1.31 (m, 2 H), 1.28-1.25 (m, 2 H), 0.90 (t, 3 H). ^{13}C NMR (150 MHz, $CDCl_3$) δ for keto-**2p**: 192.4, 192.1, 133.9, 133.0, 128.9 (2 C), 126.4 (2 C), 54.1, 31.4, 29.8, 29.3, 28.6, 22.6, 14.1; for enol-**2p**: 195.2, 168.7, 136.1, 131.7, 128.8 (2 C), 128.7 (2 C), 97.4, 31.3, 29.7, 28.6, 28.5, 22.6, 14.1.

2. Spectroscopic Data of compound 3

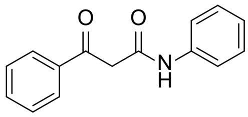

3-Oxo-*N*,3-diphenylpropanamide (3a) [3]: white solid, m.p. 106-107 °C. Keto/enol 3:1. Yield: 90% (53.8 mg). ^{1}H NMR (DMSO-d$_6$, 400 MHz) δ for keto-**3a**: 10.19 (s, 1 H), 8.39 (dd, J_1 = 8.5 Hz, J_2 = 1.3 Hz, 2 H), 7.67 (t, *J* = 7.5 Hz, 1 H), 7.56 (d, *J* = 8.0 Hz, 3 H), 7.52 (d, *J* = 7.4 Hz, 1 H), 7.30 (t, *J* = 8.2 Hz, 2 H), 7.05 (t, *J* = 7.4 Hz, 1 H), 4.13 (s, 2 H); for enol-**3a**: 14.4 (s, 1 H), 10.21 (s, 1 H), 7.74 (dd, J_1 = 7.8 Hz, J_2 = 2.8 Hz, 2 H), 7.61 (d, *J* = 8.0 Hz, 3 H), 7.56 (d, *J* = 8.0 Hz, 1 H), 7.33 (t, *J* = 7.8 Hz, 3H), 7.09 (d, *J* = 7.2 Hz, 1 H), 5.98 (s, 1 H). ^{13}C NMR (DMSO-d$_6$, 100 MHz) δ for keto-**3a**: 194.6, 165.3, 139.0, 136.3, 133.6, 128.8 (4 C), 128.4 (2 C), 123.4, 119.0 (2 C), 48.1; for enol-**3a**: 194.6, 165.3, 139.0, 136.3, 131.1, 128.5 (4 C), 125.3 (2 C), 123.4, 119.6 (2 C), 89.8.

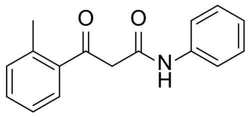

3-Oxo-*N*-phenyl-3-*o*-tolylpropanamide (3b) [3]: white solid, m.p. 81-82 °C. Keto/enol 2:1. Yield: 88% (55.7 mg). ^{1}H NMR (DMSO-d$_6$, 400 MHz) δ for keto-**3b**: 10.15 (s, 1 H), 7.86 (d, *J* = 7.7 Hz, 1 H), 7.56 (d, *J* = 8.3 Hz, 2 H), 7.44 (d, *J* = 7.5 Hz, 1 H), 7.34 (t, *J* = 8.6 Hz, 2 H), 7.29 (t, *J* = 8.4 Hz, 2 H), 7.04 (t, *J* = 7.5 Hz, 1 H), 4.07 (s, 2 H), 2.44 (s, 3 H); for enol-**3b**: 14.21 (s, 1 H), 10.14 (s, 1 H), 7.61 (d, *J* = 8.0 Hz, 2 H), 7.45 (d, *J* = 7.5 Hz, 1 H), 7.34 (t, *J* = 8.6 Hz, 2 H), 7.29 (t, *J* = 8.4 Hz, 2 H), 7.08 (t, *J* = 7.3 Hz, 1 H), 5.53 (s, 1 H), 2.43 (s, 3 H). ^{13}C NMR (DMSO-d$_6$, 100 MHz) δ for keto-**3b**: 197.9, 165.3, 139.0, 137.8, 136.9, 131.8 (2 C), 129.3, 128.8 (2 C), 125.9, 123.4, 119.2 (2 C), 50.6, 20.9; for enol-**3b**: 172.1, 170.4, 138.4, 135.7, 134.8, 131.0 (2 C), 129.9, 128.0 (2 C), 126.0, 123.6, 119.6 (2 C), 94.0, 20.3.

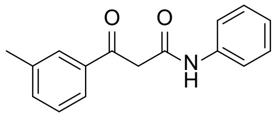

3-Oxo-*N*-phenyl-3-*m*-tolylpropanamide (3c) [3]: colorless liquid. Keto/enol 3:1. Yield: 87% (55.0 mg). ^{1}H NMR (DMSO-d$_6$, 400 MHz) δ for keto-**3c**: 10.17 (s, 1 H), 7.81 (s, 1 H), 7.80 (d, *J* = 8.4 Hz, 1 H), 7.57 (d, *J* = 7.6 Hz, 2 H), 7.47 (d, *J* = 7.5 Hz, 1 H), 7.34 -7.29(m, 3 H), 7.04 (t, *J* = 7.6 Hz, 1 H), 4.11 (s, 2 H), 2.38 (s, 3 H); for enol-**3c**: 14.31 (s, 1 H), 10.14 (s, 1 H), 7.61 (d, *J* = 7.9 Hz, 3 H), 7.57 (d, *J* = 7.6 Hz, 1 H), 7.44 (d, *J* = 7.4 Hz, 3 H), 7.36 (d, *J* = 7.6 Hz, 1 H), 7.09 (t, *J* = 7.4 Hz, 1 H), 5.97 (s, 1 H), 2.37 (s, 3 H). ^{13}C NMR (DMSO-d$_6$, 100 MHz) δ for keto-**3c**: 194.7, 165.4, 139.0, 138.2, 136.3, 134.2, 128.8 (2 C), 128.7 (2 C), 125.7, 123.4, 119.0 (2 C), 48.1, 20.9; for enol-**3c**: 170.6, 168.8, 138.5, 138.1, 133.7, 131.7, 128.9 (2 C), 128.8, 125.8, 123.6, 122.5, 119.6 (2 C), 89.8, 21.0.

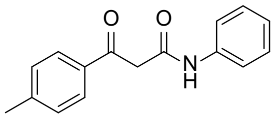

3-Oxo-*N*-phenyl-3-*p*-tolylpropanamide (3d) [3]: colorless liquid. Keto/enol 16:1. Yield: 89% (56.3 mg). ^{1}H NMR ($CDCl_3$, 400 MHz) δ 9.35 (s, 1 H), 7.93 (d, *J* = 8.2 Hz, 2 H), 7.58 (d, *J* = 7.6 Hz, 2 H), 7.35 -7.30 (m, 4 H), 7.12 (t, *J* = 7.4 Hz, 1 H), 4.09 (s, 2 H), 2.44 (s, 3 H); ^{13}C NMR ($CDCl_3$, 100 MHz) δ 196.3, 164.0, 145.7, 137.8, 133.8, 129.8 (2 C), 129.1 (2 C), 128.9 (2 C), 124.6, 120.3 (2 C), 45.4, 21.9.

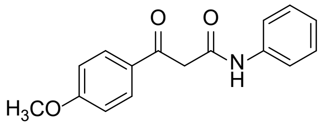

3-(4-Methoxyphenyl)-3-oxo-*N*-phenylpropanamide (3e) [3]: colorless liquid. Keto/ enol **=** 29:1. Yield: 86% (57.9 mg). ^{1}H NMR ($CDCl_3$, 400 MHz) δ 9.40 (s, 1 H), 8.02 (d, *J* = 8.8 Hz, 2 H), 7.58 (d, *J* = 7.7 Hz, 2 H), 7.33 (t, *J* = 8.2 Hz, 2 H), 7.11 (t, *J* = 7.4 Hz, 1 H), 6.97 (d, *J* = 8.9 Hz, 2 H), 4.05 (s, 2 H), 3.89 (s, 3 H); ^{13}C NMR ($CDCl_3$, 100 MHz) δ 195.0, 164.7, 161.1, 137.8, 131.3(2 C), 129.2, 129.1 (2 C), 124.6, 120.3 (2 C), 114.3 (2 C), 55.8, 45.2.

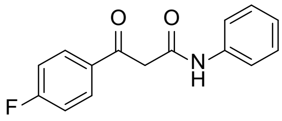

3-(4-Fluorophenyl)-3-oxo-*N*-phenylpropanamide (3f) [3]: white solid, m.p. 112-113 °C. Keto/enol 8:1. Yield: 90% (57.8 mg). ^{1}H NMR ($CDCl_3$, 400 MHz) δ 9.17 (s, 1 H), 8.04 (dd, J_1 = 8.3 Hz, J_2 = 5.4 Hz, 2 H), 7.57 (d, *J* = 7.9 Hz, 2 H), 7.33 (t, *J* = 7.7 Hz, 2 H), 7.18 (t, *J* = 8.0 Hz, 2 H), 7.13 (d, *J* = 7.5 Hz, 1 H), 4.08 (s, 2 H); ^{13}C NMR ($CDCl_3$, 100 MHz) δ 194.9, 166.6 (d, *J* = 255.7 Hz), 163.7, 137.6, 132.6 (d, *J* = 2.9 Hz), 131.6 (d, *J* = 9.6 Hz, 2 C), 129.2 (2 C), 124.8, 120.3 (2 C), 116.4 (d, *J* = 22.0 Hz, 2 C), 45.9.

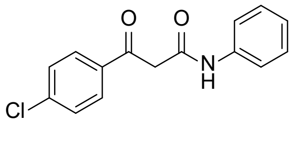

3-(4-Chlorophenyl)-3-oxo-*N*-phenylpropanamide (3g) [3]: white solid, m.p. 129-130 °C. Keto/enol 8:1. Yield: 88% (60.1 mg). ^{1}H NMR (CDCl$_3$, 400 MHz) δ 9.10 (s, 1 H), 7.99 (d, *J* = 8.6 Hz, 2 H), 7.57 (d, *J* = 7.8 Hz, 2 H), 7.50 (d, *J* = 8.6 Hz, 2 H), 7.34 (t, *J* = 8.1 Hz, 2 H), 7.13 (d, *J* = 7.4 Hz, 1 H), 4.08 (s, 2 H); ^{13}C NMR (CDCl$_3$, 100 MHz) δ 195.3, 163.4, 141.2, 137.6, 134.5, 130.2 (2 C), 129.5 (2 C), 129.2 (2 C), 124.8, 120.3 (2 C), 45.9.

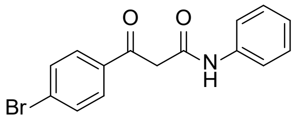

3-(4-Bromophenyl)-3-oxo-*N*-phenylpropanamide (3h) [3]: white solid, m.p. 146-147 °C. Keto/enol 6:1. Yield: 89% (57.2 mg). ^{1}H NMR (CDCl$_3$, 400 MHz) δ 9.09 (s, 1 H), 7.90 (d, *J* = 8.4 Hz, 2 H), 7.67 (d, *J* = 8.4 Hz, 2 H), 7.57 (d, *J* = 7.8 Hz, 2 H), 7.34 (t, *J* = 8.0 Hz, 2 H), 7.13 (d, *J* = 7.4 Hz, 1 H), 4.08 (s, 2 H); ^{13}C NMR (CDCl$_3$, 100 MHz) δ 195.5, 163.4, 141.2, 137.6, 134.9, 132.5 (2 C), 130.3 (2 C), 129.2 (2 C), 124.8, 120.3 (2 C), 45.9.

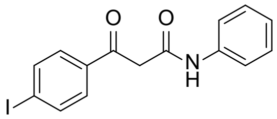

3-(4-Iodophenyl)-3-oxo-*N*-phenylpropanamide (3i) [3]: white solid, m.p. 163-164 °C. Keto/enol 5:1. Yield: 91% (83.0 mg). ^{1}H NMR (CDCl$_3$, 400 MHz) δ 9.10 (s, 1 H), 7.89 (d, *J* = 8.4 Hz, 2 H), 7.73 (d, *J* = 8.5 Hz, 2 H), 7.56 (d, *J* = 7.9 Hz, 2 H), 7.33 (t, *J* = 8.0 Hz, 2 H), 7.13 (d, *J* = 7.4 Hz, 1 H), 4.06 (s, 2 H); ^{13}C NMR (CDCl$_3$, 100 MHz) δ 195.9, 163.4, 138.5 (2 C), 137.9, 135.4, 130.2 (2 C), 129.2 (2 C), 124.8, 120.3 (2 C), 103.0, 45.8.

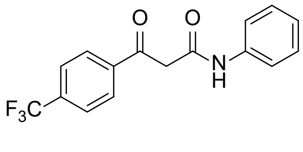

3-Oxo-*N*-phenyl-3-(4-(trifluoromethyl)phenyl)propanamide (3j) [3]: white solid, m.p. 110-111 °C. Keto/enol 6:5. Yield: 83% (63.8 mg). ^{1}H NMR

(DMSO-d6, 600 MHz) δ for keto-**3j**: 10.24 (s, 1H), 8.20 (d, *J* = 8.1 Hz, 2 H), 7.93 (t, *J* = 8.1 Hz, 2 H), 7.57 (d, *J* = 7.7 Hz, 2 H), 7.31 (t, *J* = 7.8 Hz, 2 H), 7.05 (t, *J* = 7.4 Hz, 1 H), 4.22 (s, 2 H); for enol-**3j**: 14.39 (s, 1 H), 10.33 (s, 1H), 7.93 (d, *J* = 8.1 Hz, 2H), 7.87 (d, *J* = 8.3 Hz, 2 H), 7.63 (d, *J* = 7.9 Hz, 2 H), 7.35 (d, *J* = 7.8 Hz, 2 H), 7.10 (t, *J* = 7.4 Hz, 1 H), 6.10 (s, 1H); ^{13}C NMR (DMSO-d6, 151 MHz) δ for keto-**3j**: 194.2, 165.0, 138.9, 138.2, 132.8 (q, *J* = 32.0 Hz), 129.2 (2 C), 128.8 (2 C), 125.8 (q, *J* = 3.6 Hz, 2 C), 123.8 (q, *J* = 272.5 Hz), 123.5, 119.1 (2 C), 48.7; for enol-**3j**: 170.2, 166.7, 139.5, 137.6, 130.7 (q, *J* = 32.0 Hz), 128.9 (2 C), 126.1 (2 C), 125.8 (q, *J* = 3.6 Hz, 2 C), 123.9 (q, *J* = 272.5 Hz), 123.8, 119.7 (2 C), 91.7.

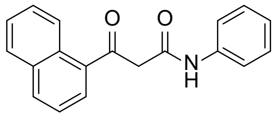

3-(Naphthalen-1-yl)-3-oxo-*N*-phenylpropanamide (3k) [3]: white solid, m.p. 164-165 °C. Keto/enol 5:1. Yield: 86% (62.2 mg). ^{1}H NMR ($CDCl_3$, 400 MHz) δ 9.08 (s, 1 H), 8.63 (d, *J* = 8.5 Hz, 2 H), 7.98 (d, *J* = 7.6 Hz, 2 H), 7.83 (d, *J* = 8.1 Hz, 1 H), 7.59-7.44 (m, 4 H), 7.27 (t, *J* = 7.2 Hz, 2 H), 7.05 (d, *J* = 7.3 Hz, 1 H), 4.13 (s, 2 H); ^{13}C NMR ($CDCl_3$, 100 MHz) δ 199.9, 163.9, 137.7, 134.6, 134.1, 129.8, 129.2 (2 C), 128.9, 128.8, 127.0, 125.7, 125.6, 125.1, 124.7, 124.6, 120.3 (2 C), 49.5.

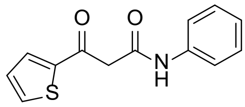

3-Oxo-*N*-phenyl-3-(thiophen-2-yl)propanamide (3l) [3]: colorless liquid. Keto/enol 25:1. Yield: 85% (52.1 mg). ^{1}H NMR (DMSO-d6, 400 MHz) δ 10.26 (s, 1 H), 8.06-8.03 (m, 2 H), 7.57 (d, *J* = 7.9 Hz, 2 H), 7.32 (m, 3 H), 7.05 (t, *J* = 7.4 Hz, 1 H), 4.08 (s, 2 H); ^{13}C NMR (DMSO-d6, 100 MHz) δ 187.3, 164.8, 143.5, 138.9, 135.7, 134.6, 128.9, 128.8 (2 C), 123.5, 119.1 (2 C), 48.3.

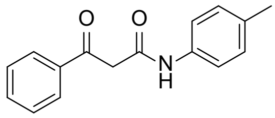

3-Oxo-3-phenyl-*N*-*p*-tolylpropanamide (3m) [3]: white solid, m.p. 131-132 °C. Keto/ enol **=** 3:1. Yield: 90% (56.9 mg). ^{1}H NMR (DMSO-d$_6$, 400 MHz) δ for keto-**3m**: 10.11 (s, 1 H), 8.00 (dd, J_1 = 8.5 Hz, J_2 = 1.3 Hz, 2 H), 7.66 (t, J = 7.5 Hz, 1 H), 7.55 (t, J = 7.8 Hz, 2 H), 7.46 (d, J = 8.4 Hz, 2 H), 7.09 (d, J = 8.3 Hz, 2 H), 4.12 (s, 2 H), 2.24 (s, 3 H); for enol-**3m**: 14.42 (s, 1 H), 10.13 (s, 1 H), 7.75-7.73 (m, 2 H), 7.51-7.49 (m, 5 H), 7.14 (d, J = 7.5 Hz, 2 H), 5.97 (s, 1 H), 2.43 (s, 3 H). ^{13}C NMR (DMSO-d$_6$, 100 MHz) δ for keto-**3m**: 194.6, 165.0, 136.5, 136.3, 133.5, 132.3, 129.1 (2 C), 128.8 (2 C), 128.3 (2 C), 119.0 (2 C), 48.0, 20.4; for enol-**3m**: 170.4, 168.5, 135.8, 133.7, 132.6, 130.9, 129.3 (2 C), 128.8 (2 C),125.3 (2 C), 119.6 (2 C), 89.8, 20.4.

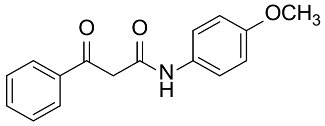

***N*-(4-Methoxyphenyl)-3-oxo-3-phenylpropanamide (3n)** [3]: white solid, m.p. 129-130 °C. Keto/enol 3:1. Yield: 87% (58.5 mg). ^{1}H NMR (DMSO-d$_6$, 400 MHz) δ for keto-**3n**: 10.06 (s, 1 H), 8.00 (dd, J_1 = 8.5 Hz, J_2 = 1.3 Hz, 2 H), 7.66 (t, J = 7.4 Hz, 1 H), 7.56 (d, J = 7.8 Hz, 2 H), 7.48 (dd, J_1 = 7.0 Hz, J_2 = 2.0 Hz, 2 H), 6.87 (d, J_1 = 7.0 Hz, J_2 = 2.0 Hz, 2 H), 4.10 (s, 2 H), 3.71 (s, 3 H); for enol-**3n**: 14.44 (s, 1 H), 10.08 (s, 1 H), 7.75-7.72 (m, 2 H), 7.54-7.50 (m, 5 H), 6.92 (d, J = 8.8 Hz, 2 H), 5.94 (s, 1 H), 3.73 (s, 3 H). ^{13}C NMR (DMSO-d$_6$, 100 MHz) δ for keto-**3n**: 194.6, 164.7, 155.3, 136.3, 133.5, 132.1, 128.8 (2 C), 128.4 (2 C), 120.6 (2 C), 113.9 (2 C), 55.1, 47.9; for enol-**3n**: 170.1, 168.2, 155.6, 133.8, 131.4, 130.9, 128.8 (2 C),125.2 (2 C), 121.2 (2 C), 114.0 (2 C), 89.8, 55.2.

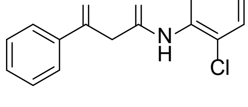

***N*-(2-Chlorophenyl)-3-oxo-3-phenylpropanamide (3o)** [3]: white solid, m.p. 139-140 °C. Keto/enol 3:1. Yield: 85% (58.0 mg). ^{1}H NMR (DMSO-d$_6$, 400

MHz) δ for keto-**3o**: 9.85 (s, 1 H), 8.01 (d, J = 7.4 Hz, 2 H), 7.83 (d, J = 7.4 Hz, 1 H), 7.67 (d, J = 7.4 Hz, 1 H), 7.55 (t, J = 7.8 Hz, 2 H), 7.49 (td, J_1 = 8.0 Hz, J_2 = 1.2 Hz, 2 H), 7.18 (td, J_1 = 8.8 Hz, J_2 = 1.0 Hz, 1 H), 4.26 (s, 2 H); for enol-**3o**: 14.20 (s, 1 H), 9.76 (s, 1 H), 7.78-7.75 (m, 4 H), 7.37 (d, J = 7.3 Hz, 1 H), 7.32 (td, J_1 = 8.5 Hz, J_2 = 1.0 Hz, 3 H), 7.23 (t, J = 7.3 Hz, 1 H), 6.25 (s, 1 H); ^{13}C NMR (DMSO-d_6, 100 MHz) δ for keto-**3o**: 194.6, 165.9, 136.2, 134.8, 133.6, 129.5, 128.8 (2 C), 128.3 (2 C), 127.5, 126.1, 125.4, 125.3, 47.5; for enol-**3o**: 171.1, 169.0, 134.1, 133.5, 131.6, 129.6, 128.9 (2 C), 128.3 (2 C), 127.5, 126.6, 125.6 (2 C), 89.6.

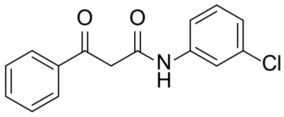

***N*-(3-Chlorophenyl)-3-oxo-3-phenylpropanamide (3p)** [3]: white solid, m.p. 117-118 ^{o}C. Keto/enol 3:1. Yield: 87% (59.4 mg). ^{1}H NMR (DMSO-d_6, 400 MHz) δ for keto-**3p**: 10.38 (s, 1 H), 8.01 (dd, J_1 = 8.5 Hz, J_2 = 1.3 Hz, 2 H), 7.80 (d, J = 2.4 Hz, 1 H), 7.67 (dt, J_1= 7.4 Hz, J_2 = 1.2 Hz, 1 H), 7.55 (t, J = 7.8 Hz, 2 H), 7.52 (t, J = 7.8 Hz, 1 H), 7.33 (t, J = 8.0 Hz, 1 H), 7.13-7.10 (m, 1 H), 4.16 (s, 2 H); for enol-**3p**: 14.16 (s, 1 H), 10.38 (s, 1 H), 7.83 (t, J = 2.4 Hz, 1 H), 7.76-7.74 (m, 2 H), 7.45 (d, J = 8.2 Hz, 1 H), 7.41-7.39 (m, 3 H), 7.35 (d, J = 8.0 Hz, 1 H), 7.15-7.12 (m, 1 H), 5.96 (s, 1 H); ^{13}C NMR (DMSO-d_6, 100 MHz) δ for keto-**3p**: 194.4, 165.8, 140.3, 136.2, 133.6, 133.1, 130.5, 128.8 (2 C), 128.3 (2 C), 123.1, 118.5, 117.4, 48.1; for enol-**3p**: 170.8, 169.2, 139.9, 133.5, 133.2, 130.6, 130.5, 128.9 (2 C), 125.4 (2 C), 123.3, 119.0, 118.0, 89.7.

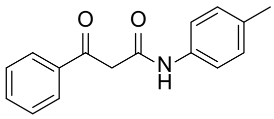

***N*-(4-Chlorophenyl)-3-oxo-3-phenylpropanamide (3q)** [3]: white solid, m.p. 160-161 ^{o}C. Keto/enol 3:1. Yield: 90% (61.4 mg). ^{1}H NMR (DMSO-d_6, 400 MHz) δ for keto-**3q**: 10.32 (s, 1 H), 7.99 (dd, J_1 = 8.5 Hz, J_2 = 1.3 Hz, 2 H), 7.64 (d, J = 7.3 Hz, 1 H), 7.60 (d, J = 8.8 Hz, 2 H), 7.56 (d, J = 7.9 Hz, 2 H), 7.35 (d, J = 8.8 Hz, 2 H), 4.15 (s, 2 H); for enol-**3q**: 14.24 (s, 1 H), 10.32 (s, 1

H), 7.75 (dd, J_1 = 7.9 Hz, J_2 = 2.4 Hz, 2 H), 7. 67 (d, J = 7.4 Hz, 2 H), 7.53-7.50 (m, 3 H), 7.39 (d, J = 8.9 Hz, 2 H), 5.96 (s, 1 H); ^{13}C NMR (DMSO-d$_6$, 100 MHz) δ for keto-**3q**: 194.5, 165.5, 137.9, 136.2, 133.6, 128.8 (3 C), 128.7 (2 C), 128.3 (2 C), 120.5 (2 C), 48.0; for enol-**3q**: 170.6, 169.0, 137.4, 133.5, 131.1, 128.9 (2 C), 126.9 (2 C), 125.3 (2 C), 121.1 (2 C), 104.5, 89.7.

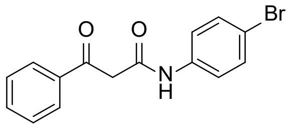

***N*-(4-Bromophenyl)-3-oxo-3-phenylpropanamide (3r)** [3]: white solid, m.p. 176-177 °C. Keto/enol 3:1. Yield: 84% (66.6 mg). ^{1}H NMR (DMSO-d$_6$, 400 MHz) δ for keto-**3r**: 10.32 (s, 1 H), 8.00 (d, J = 7.5 Hz, 2 H), 7.66 (t, J = 7.2 Hz, 1 H), 7.60-7.47 (m, 6 H), 4.15 (s, 2 H); for enol-**3r**: 14.24 (s, 1 H), 10.32 (s, 1 H), 7.75 (d, J = 7.2 Hz, 2 H), 7.60-7.47 (m, 7 H), 5.96 (s, 1 H); ^{13}C NMR (DMSO-d$_6$, 100 MHz) δ for keto-**3r**: 194.4, 165.5, 138.3, 136.2, 133.6, 131.6 (2 C), 128.8 (2 C), 128.3 (2 C), 120.9 (2 C), 114.9, 48.1; for enol-**3r**: 170.6, 169.0, 137.8, 133.5, 131.7 (2 C), 131.1, 128.8 (2 C), 125.4 (2 C), 121.5 (2 C), 115.3, 89.7.

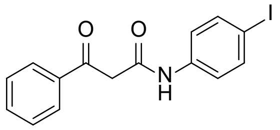

***N*-(4-Iodophenyl)-3-oxo-3-phenylpropanamide (3s)** [3]: white solid, m.p. 179-180 °C. Keto/enol 3:1. Yield: 86% (78.5 mg). ^{1}H NMR (DMSO-d$_6$, 400 MHz) δ for keto-**3s**: 10.29 (s, 1 H), 7.99 (dd, J_1 = 8.5 Hz, J_2 = 1.2 Hz, 2 H), 7.67 (d, J = 8.5 Hz, 1 H), 7.64 (d, J = 8.7 Hz, 2 H), 7.56 (d, J = 7.8 Hz, 1 H), 7.51 (t, J = 7.1 Hz, 1 H), 7.42 (d, J = 8.7 Hz, 2 H), 4.14 (s, 2 H); for enol-**3s**: 14.24 (s, 1 H), 10.29 (s, 1 H), 7.74 (dd, J_1 = 7.9 Hz, J_2 = 2.3 Hz, 1 H), 7.67 (d, J = 8.5 Hz, 1 H), 7.64 (d, J = 8.7 Hz, 2 H), 7.56 (d, J = 7.8 Hz, 2 H), 7.51 (t, J = 7.1 Hz, 2 H), 7.45 (d, J = 8.5 Hz, 1 H), 5.96 (s, 1 H); ^{13}C NMR (DMSO-d$_6$, 100 MHz) δ for keto-**3s**: 194.4, 165.5, 137.4 (2 C), 136.2, 133.6, 128.8 (2 C), 128.3 (2 C), 125.3, 121.2 (2 C), 86.8, 48.1; for enol-**3s**: 170.6, 169.0, 138.3, 137.5 (2 C), 133.5, 131.1, 128.8 (2 C), 125.3 (2 C), 121.7 (2 C), 89.8, 87.2.

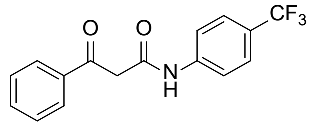

3-Oxo-3-phenyl-*N*-(4-(trifluoromethyl)phenyl)propanamide (3t) [3]: white solid, m.p. 165-166 °C. Keto/enol 3:1. Yield: 89% (68.3 mg). ^{1}H NMR (DMSO-d$_6$, 400 MHz) δ for keto-**3t**: 10.55 (s, 1 H), 8.00 (d, *J* = 7.6 Hz, 2 H), 7.84-7.76 (m, 2 H), 7.71-7.66 (m, 3 H), 7.57 (d, *J* = 7.6 Hz, 2 H), 4.20 (s, 2 H); for enol-**3t**: 14.16 (s, 1 H), 10.55 (s, 1 H), 7.84-7.76 (m, 3 H), 7.71-7.66 (m, 3 H), 7.54-7.51 (m, 3 H), 6.00 (s, 1 H); ^{13}C NMR (DMSO-d$_6$, 100 MHz) δ for keto-**3t**: 194.42, 166.12, 142.49, 136.15, 133.69, 133.55 (q, *J* = 29.0 Hz), 128.84 (2 C), 128.37 (2 C), 126.15 (q, *J* = 3.7 Hz), 125.44, 124.35(q, *J* = 269.5 Hz), 118.90 (2 C), 48.1; for enol-**3t**: 171.03, 169.51, 142.10, 133.55 (q, *J* = 29.0 Hz), 131.32, 128.92 (2 C), 128.37 (2 C), 126.15 (q, *J* = 3.7 Hz), 124.35(q, *J* = 269.5 Hz), 123.56, 123.24, 119.46 (2 C), 89.73.

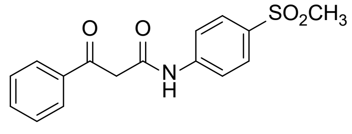

***N*-(4-(Methylsulfonyl)phenyl)-3-oxo-3-phenylpropanamide (3u)** [3b]: white solid, m.p. 118-119 °C. Keto/enol 3:1. Yield: 82% (65.0 mg). ^{1}H NMR (DMSO-d$_6$, 400 MHz) δ for keto-**3u**: 10.63 (s, 1 H), 8.01-7.50 (m, 7 H), 6.63 (d, *J* = 7.0 Hz, 1 H), 6.07 (s, 1 H), 4.22 (s, 2 H), 3.15 (s, 3 H); for enol-**3u**: 14.12 (s, 1 H), 10.63 (s, 1 H), 8.01-7.50 (m, 7 H), 6.63 (d, *J* = 7.0 Hz, 1 H), 6.07 (s, 1 H), 6.02 (s, 1 H), 3.01 (s, 3 H); ^{13}C NMR (DMSO-d$_6$, 100 MHz) δ for keto-**3u**: 194.4, 166.3, 153.5, 143.4, 134.8, 133.7, 128.9 (2 C), 128.4 (2 C), 128.3 (2 C), 118.8, 112.7, 48.2, 43.8; for enol-**3u**: 171.1, 169.7, 143.0, 136.1, 134.9, 133.4, 131.4, 128.9 (2 C), 125.8 (2 C), 125.5 (2 C), 119.3, 89.8, 44.5.

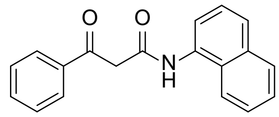

***N*-(Naphthalen-1-yl)-3-oxo-3-phenylpropanamide (3v)** [3]: white solid, m.p. 173-174 °C. Keto/enol 3:1. Yield: 89% (64.3 mg). ^{1}H NMR (DMSO-d$_6$, 400 MHz) δ for keto-**3v**: 10.19 (s, 1 H), 8.17 (d, *J* = 7.7 Hz, 1 H), 8.08 (d, *J* = 7.5 Hz,

2 H), 7.94 (d, J = 7.4 Hz, 1 H), 7.78-7.67 (m, 3 H), 7.60-7.47 (m, 5 H), 4.33 (s, 2 H); for enol-**3v**: 14.44 (s, 1 H), 10.19 (s, 1 H), 8.17 (d, J = 7.7 Hz, 1 H), 8.08 (d, J = 7.5 Hz, 1 H), 7.94 (d, J = 7.4 Hz, 1 H), 7.78-7.67 (m, 3 H), 7.60-7.47 (m, 6 H), 6.31 (s, 1 H); ^{13}C NMR (DMSO-d$_6$, 100 MHz) δ for keto-**3v**: 194.9, 166.1, 136.3, 133.6, 133.3, 128.8 (2 C), 128.3 (2 C), 128.1, 126.4, 125.9, 125.6 (2 C), 125.3 (2 C), 122.7, 121.4, 47.8; for enol-**3v**: 171.4, 168.8, 133.7, 131.0, 128.8, 128.2 (2 C), 127.6 (2 C), 126.0, 125.9, 125.6 (2 C), 125.4, 126.1, 125.3 (2 C), 122.5, 89.6.

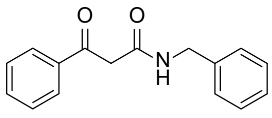

***N*-Benzyl-3-oxo-3-phenylpropanamide (3w)** [3]: colorless liquid. Keto/enol 3:1. Yield: 80% (50.6 mg). ^{1}H NMR (DMSO-d$_6$, 400 MHz) δ for keto-**3w**: 8.63 (s, 1 H), 7.97 (d, J = 7.4 Hz, 2 H), 7.70-7.68 (m, 1 H), 7.65 (t, J = 7.4 Hz, 1 H), 7.53 (t, J = 7.6 Hz, 2 H), 7.48-7.46 (m, 1 H), 7.35-7.22 (m, 5 H), 4.30 (d, J = 5.8 Hz, 2 H); for enol-**3w**: 14.68 (s, 1 H), 8.63 (s, 1 H), 7.70-7.68 (m, 1 H), 7.48-7.46 (m, 1 H), 7.35-7.22 (m, 8 H), 5.83 (s, 1 H), 4.40 (d, J = 5.8 Hz, 2 H); ^{13}C NMR (DMSO-d$_6$, 100 MHz) δ for keto-**3w**: 194.8, 166.3, 139.2, 136.3, 133.5, 128.7 (2 C), 128.4 (2 C), 128.3 (2 C), 127.2 (2 C), 125.2, 46.9, 42.2; for enol-**3w**: 171.7, 167.5, 138.9, 133.9, 133.5, 128.7 (2 C), 128.4 (2 C), 127.4 (2 C), 127.0, 126.8 (2 C), 89.0, 41.9.

3. References

[1] Xu, Q.; Zheng, B. H.; Pan, L.; Liu, Q.; Li, Y. F. *Eur. J. Org. Chem.* **2019**, *2019*, 3704–3710.

[2] Qi, F.; Yu, H. F.; Wang, Y. N.; Lv, Y.; Li, Y. X.; Han, L.; Wang, R.; Feng, X. N. *Synthetic. Commun.* **2017**, *47*(23), 2220−2224.

[3] Li, L. L.; Jiang, C. X.; Peng, T. T.; Zhang, N. N.; Jia, J.; Yu, H. F.; Zhao, X. B. *Synthetic Commun*. **2023**, *54*(4), 312-320.

4. Copies of ^{1}H NMR and ^{13}C NMR spectra of compound 2

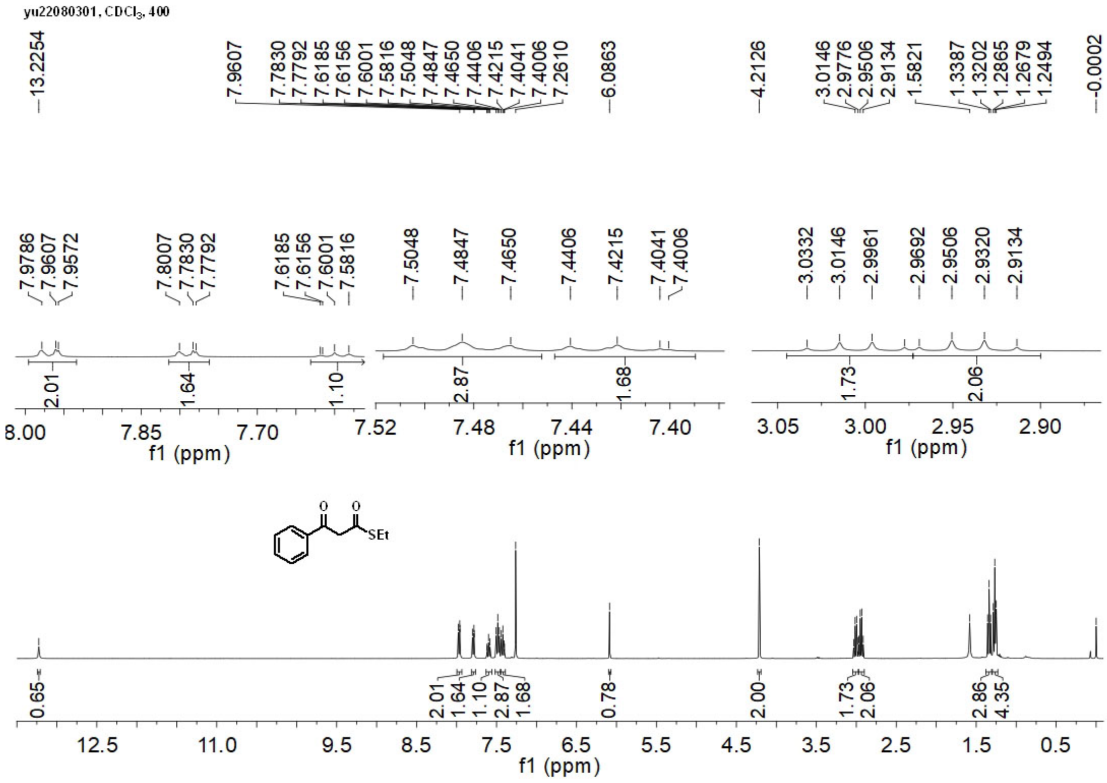

^{1}H NMR of **2a**

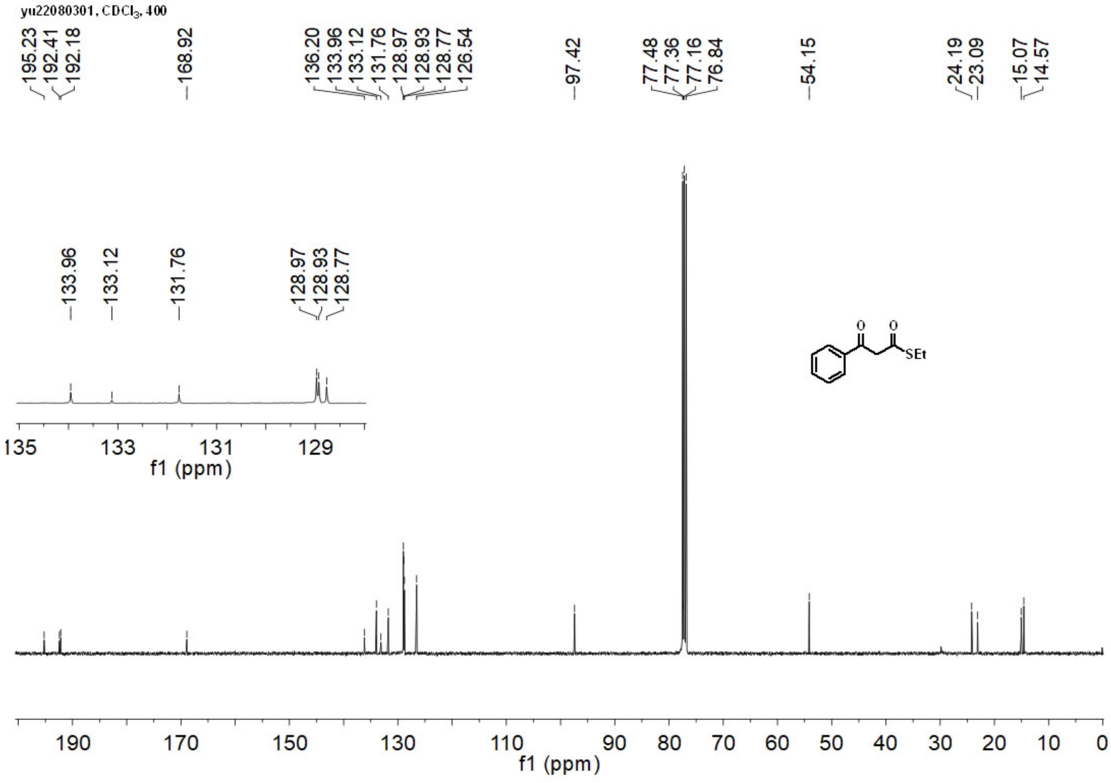

^{13}C NMR of **2a**

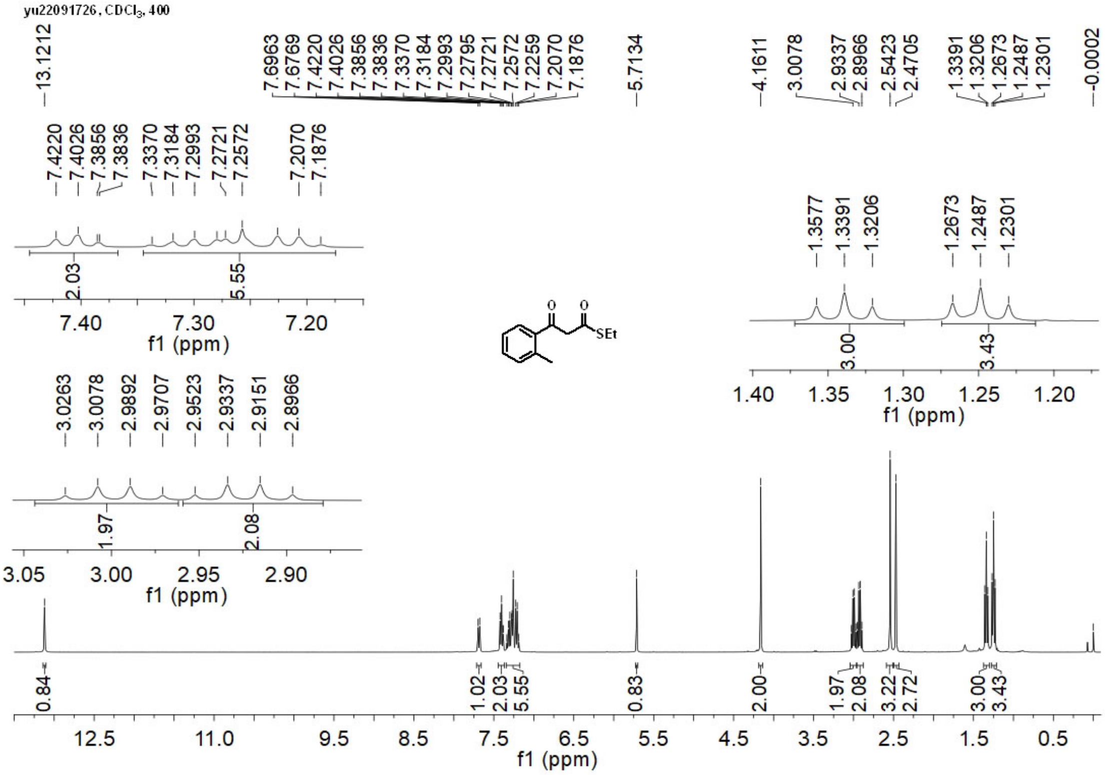

^{1}H NMR of **2b**

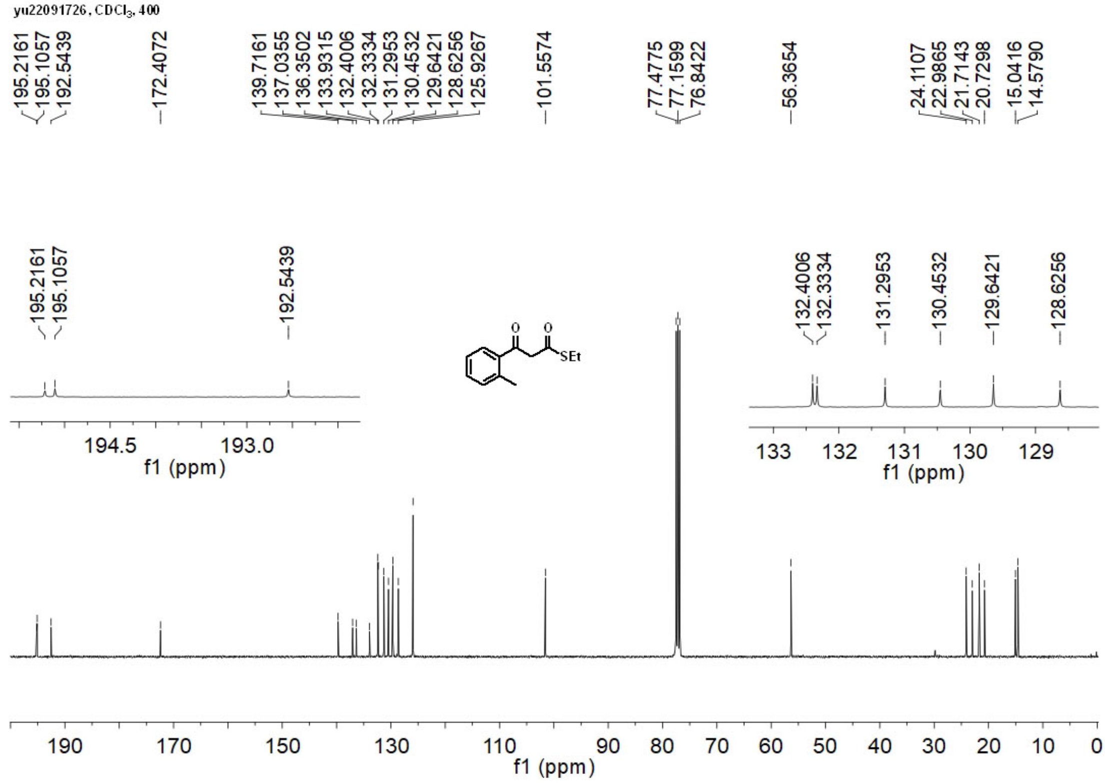

^{13}C NMR of **2b**

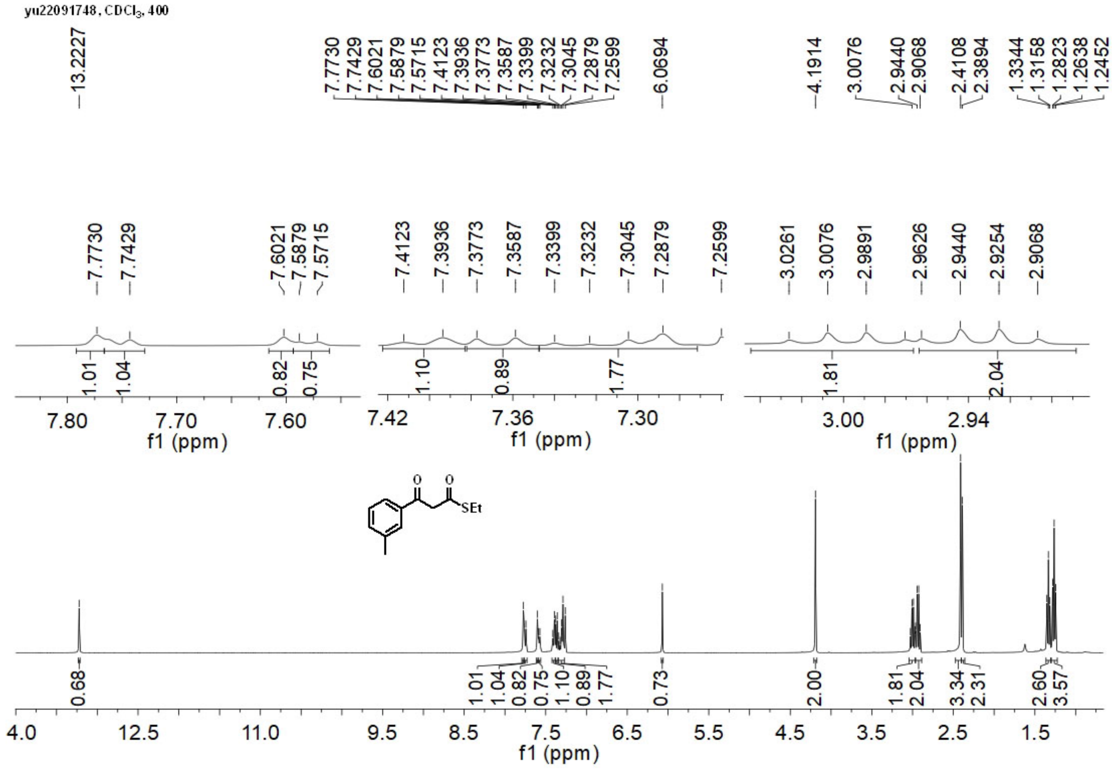

^{1}H NMR of **2c**

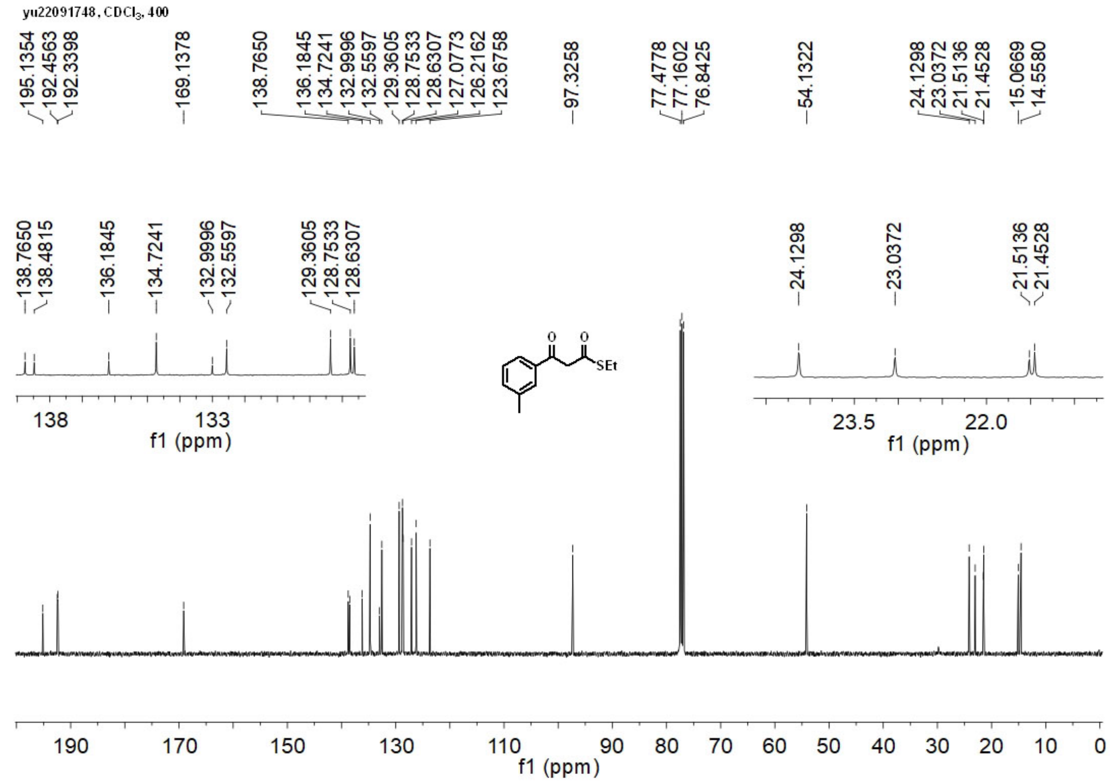

^{13}C NMR of **2c**

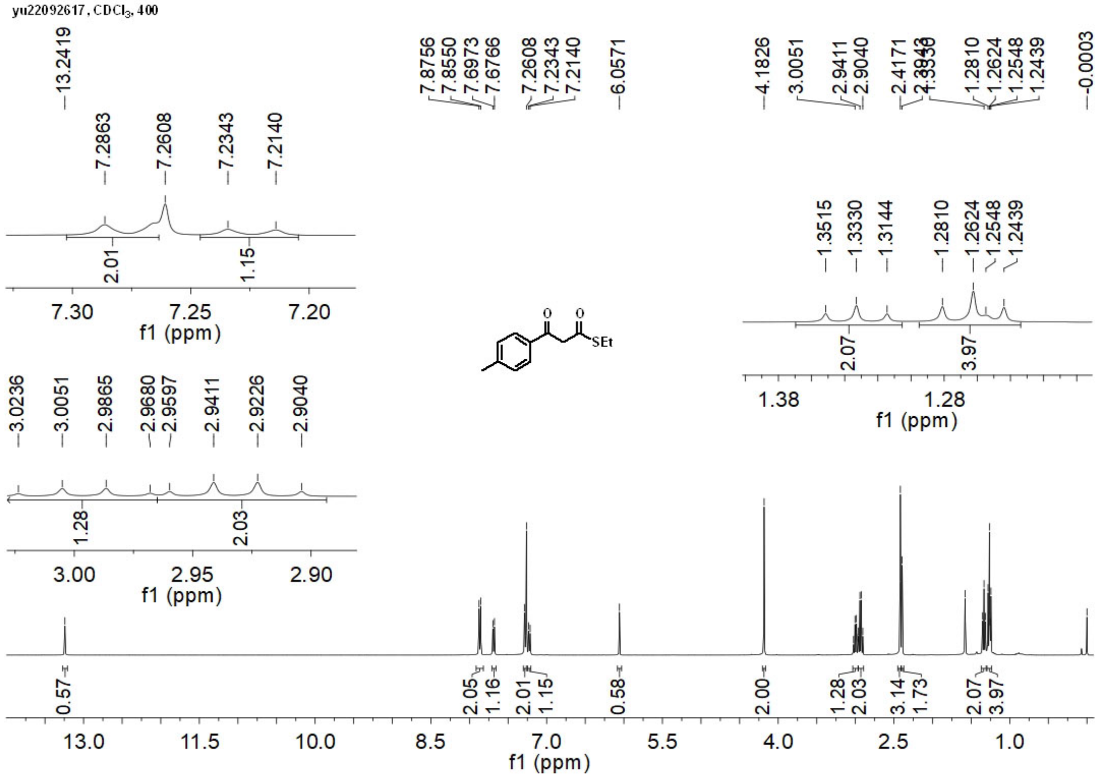

^{1}H NMR of **2d**

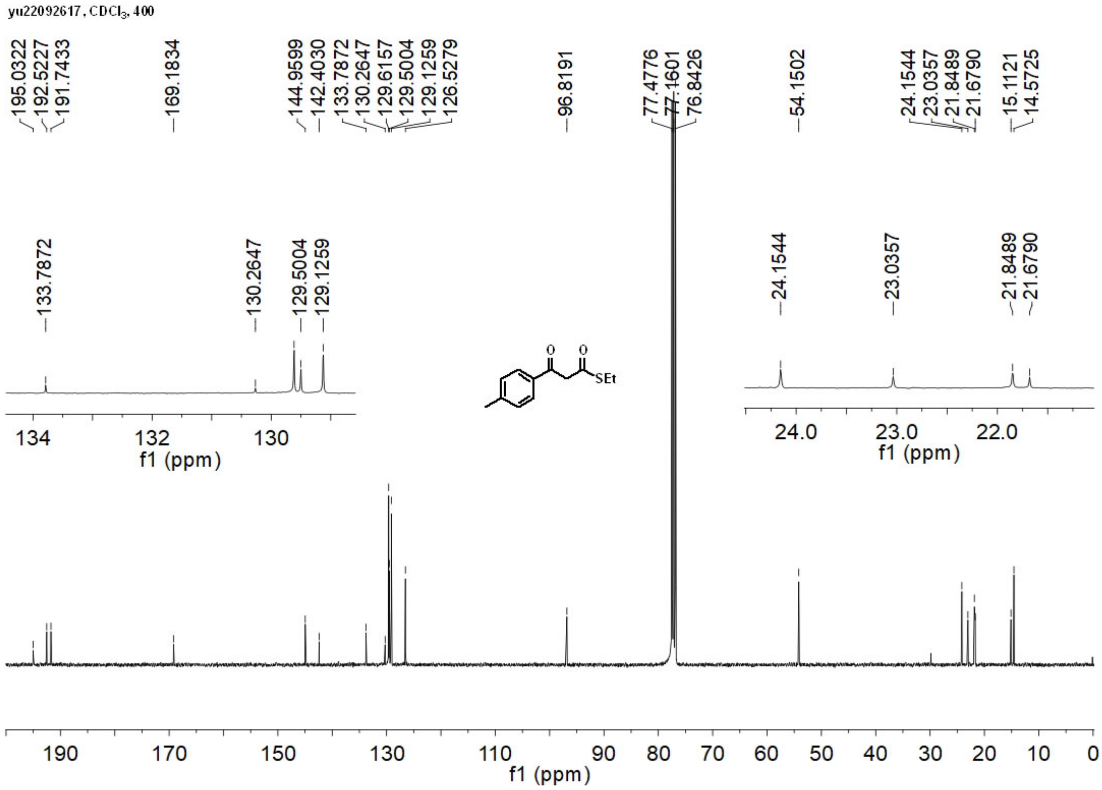

^{13}C NMR of **2d**

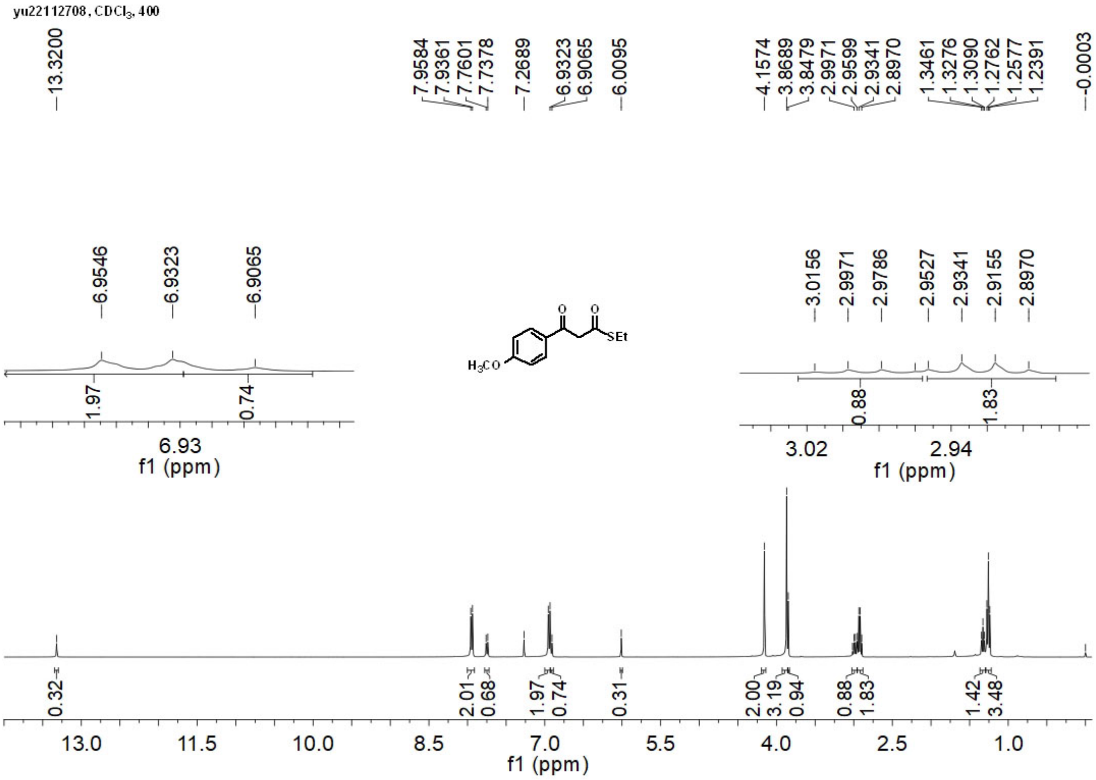

^{1}H NMR of **2e**

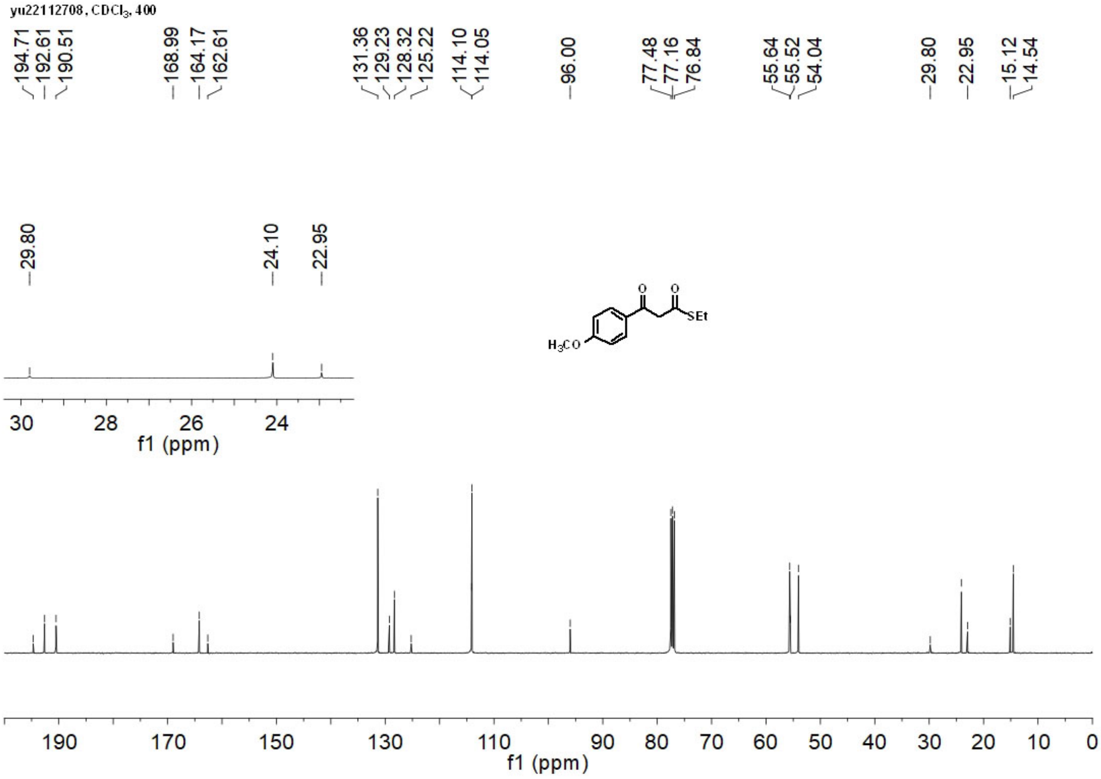

^{13}C NMR of **2e**

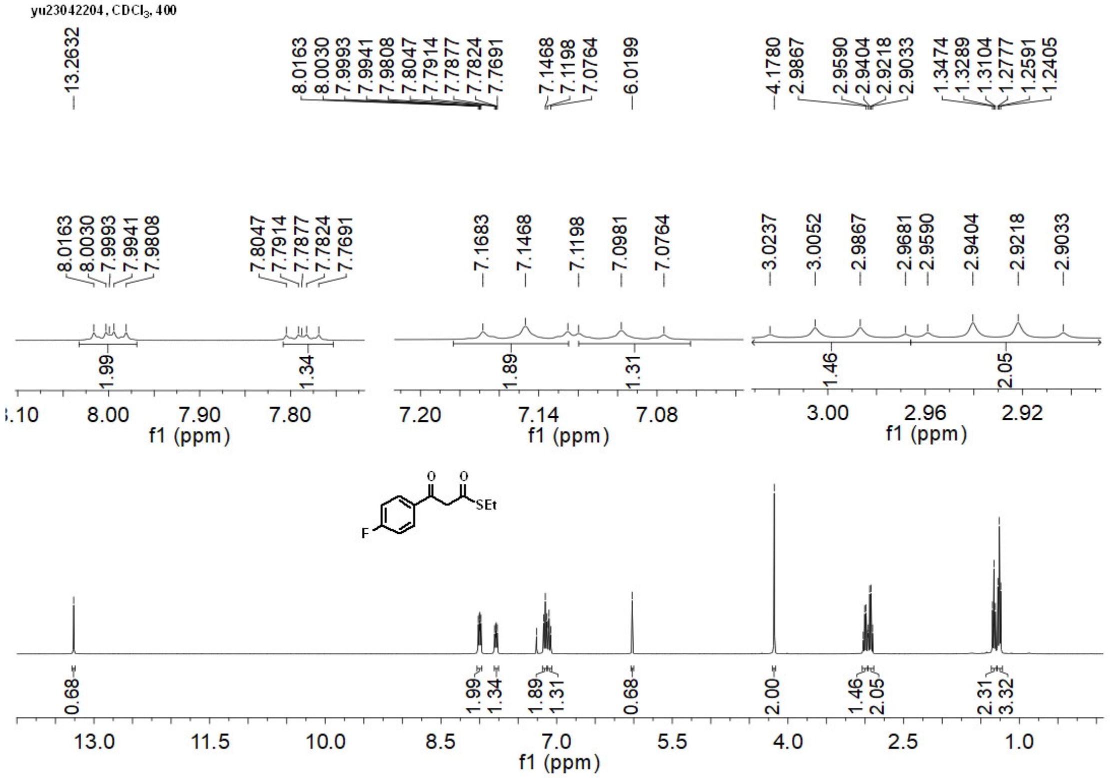

^{1}H NMR of **2f**

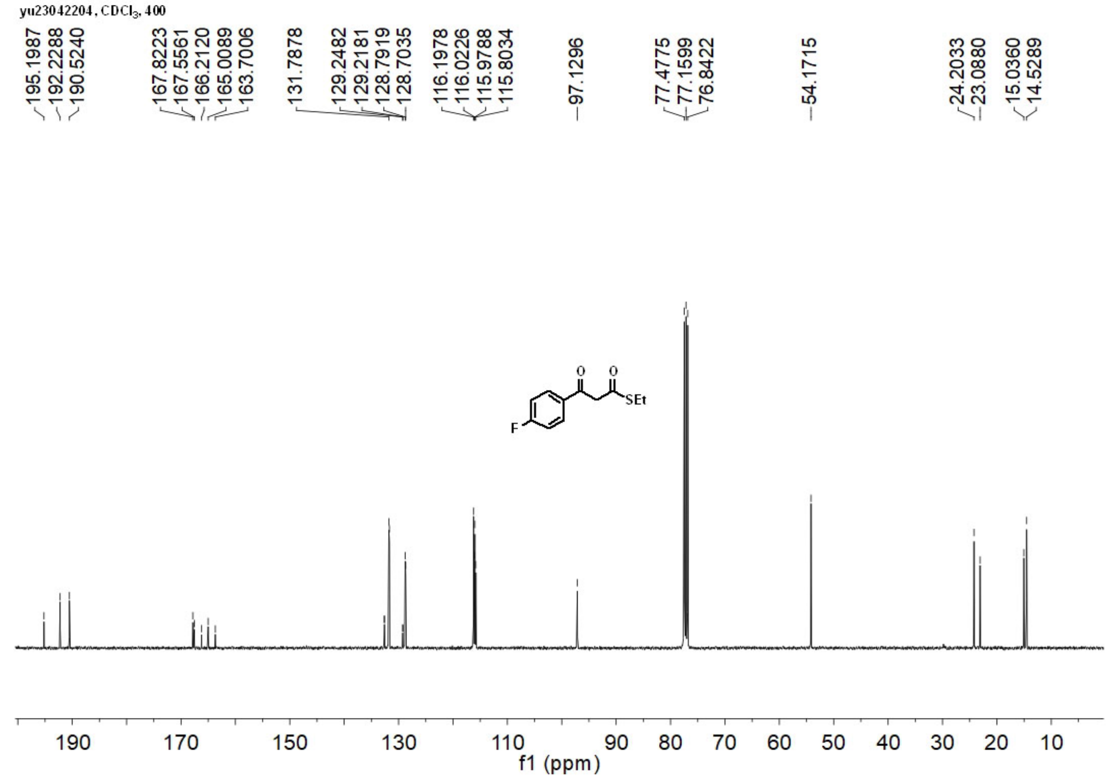

^{13}C NMR of **2f**

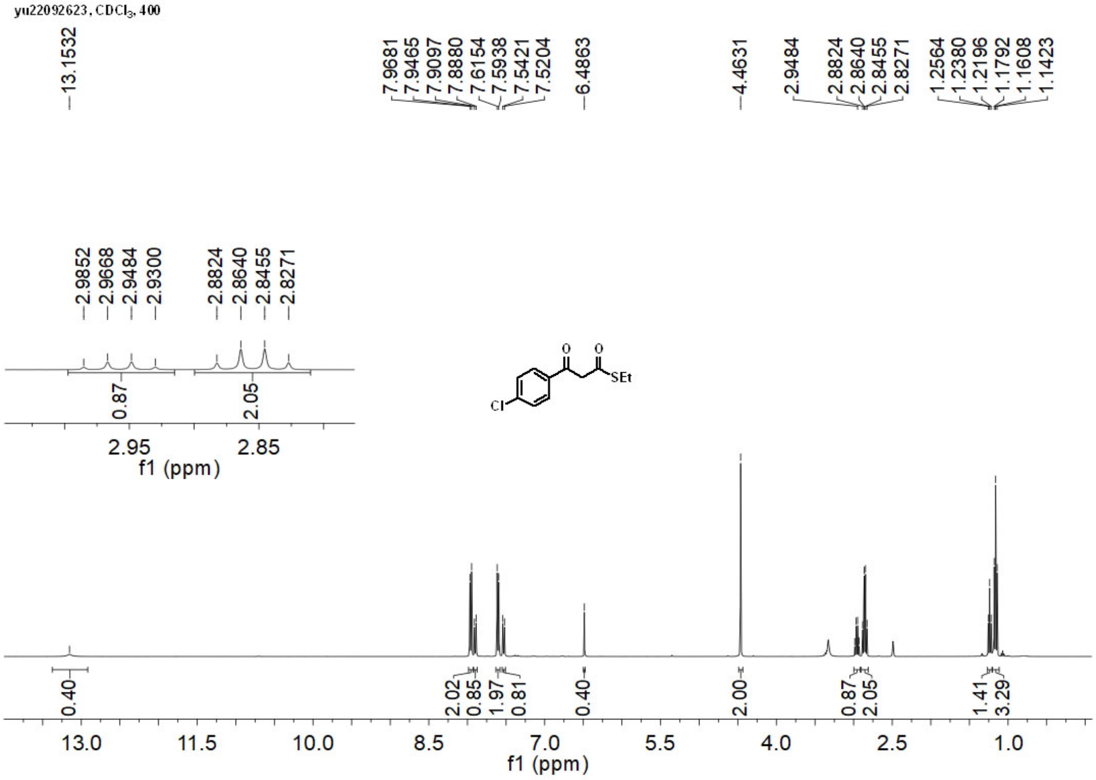

^{1}H NMR of **2g**

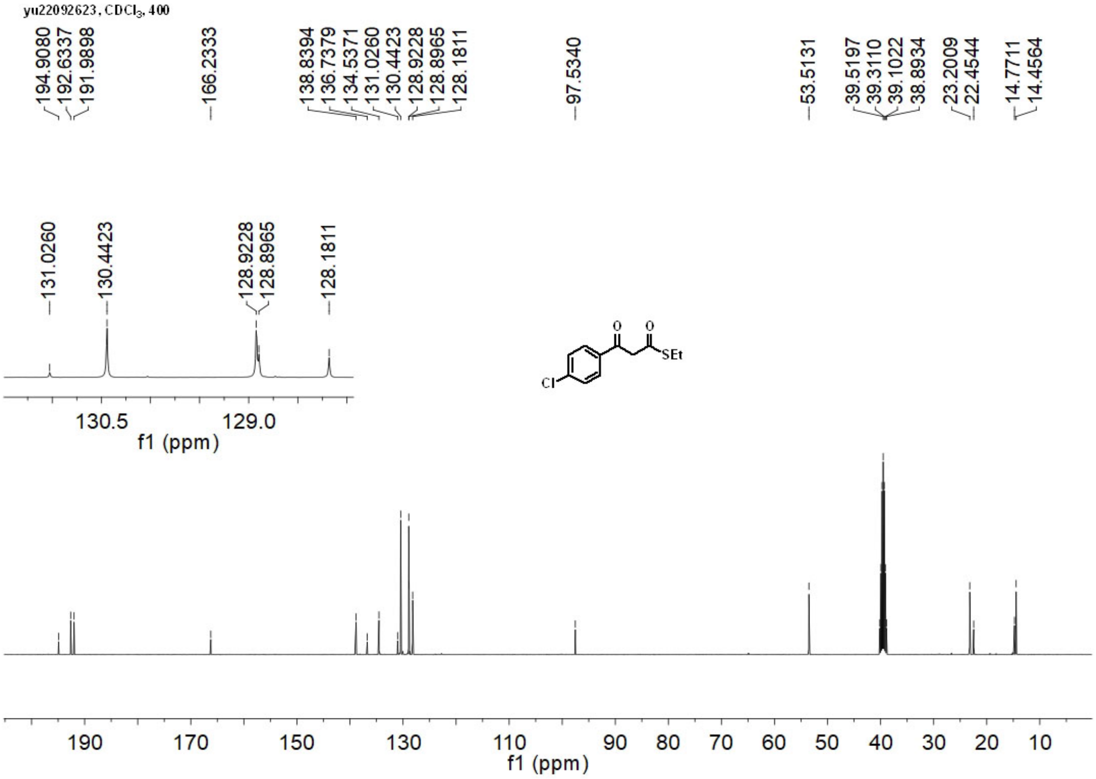

^{13}C NMR of **2g**

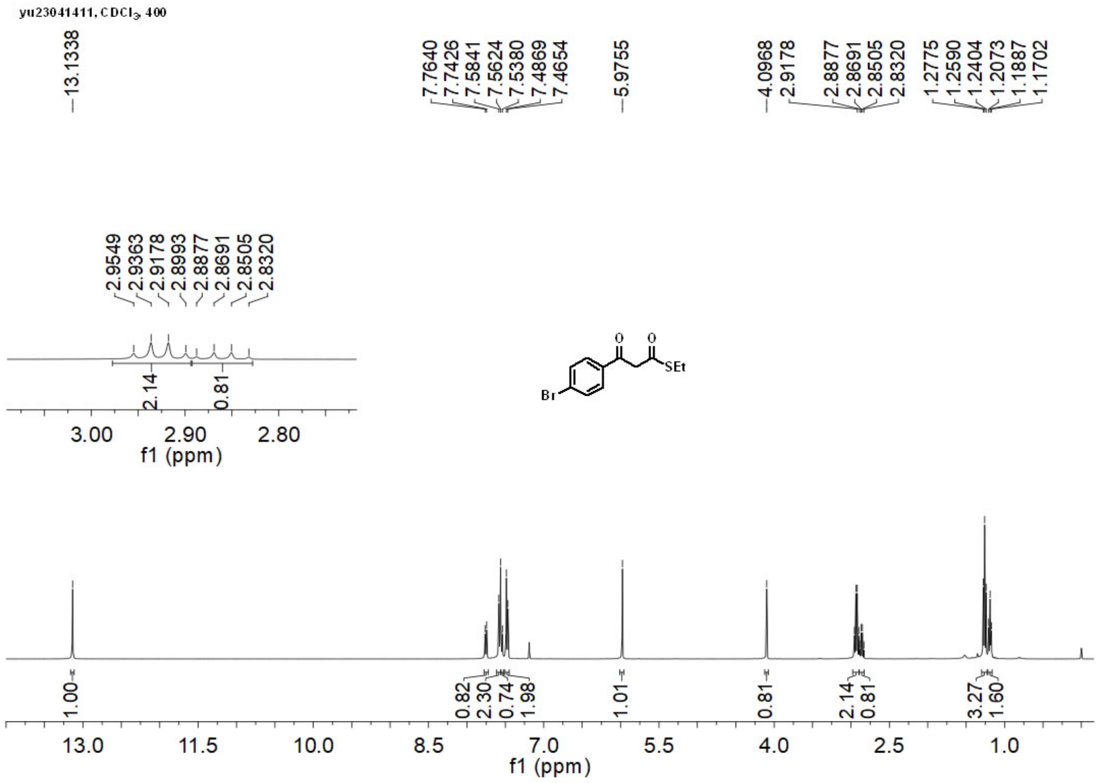

^{1}H NMR of **2h**

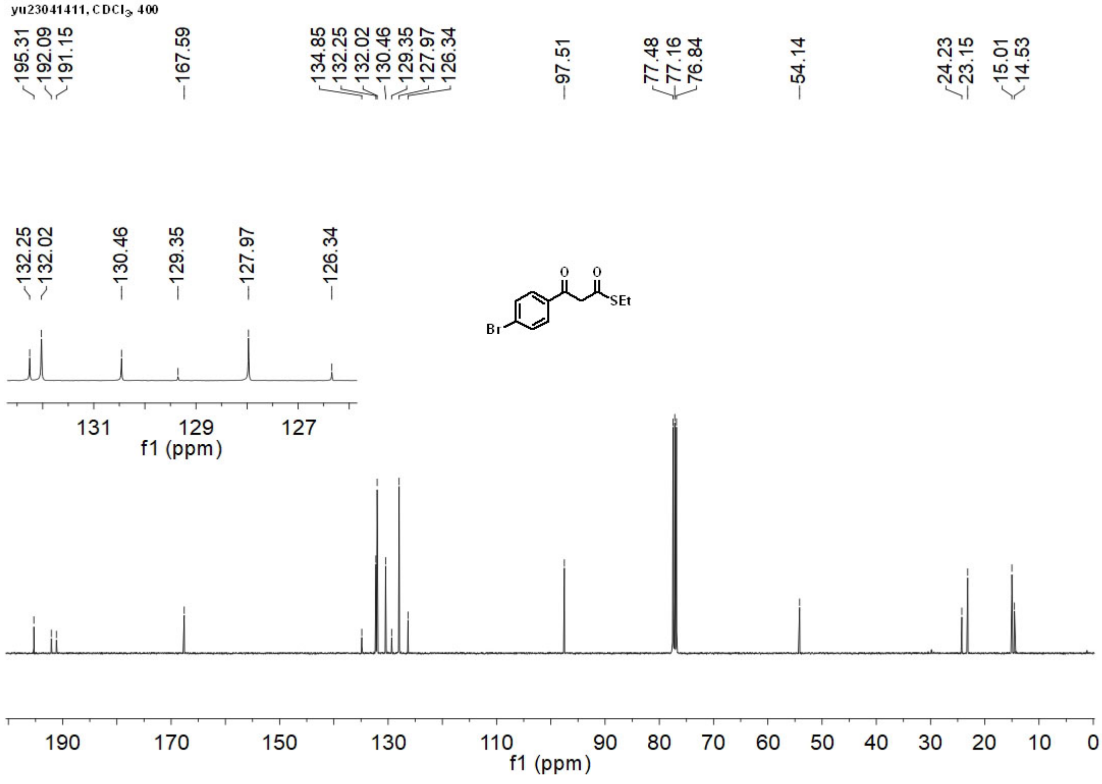

^{13}C NMR of **2h**

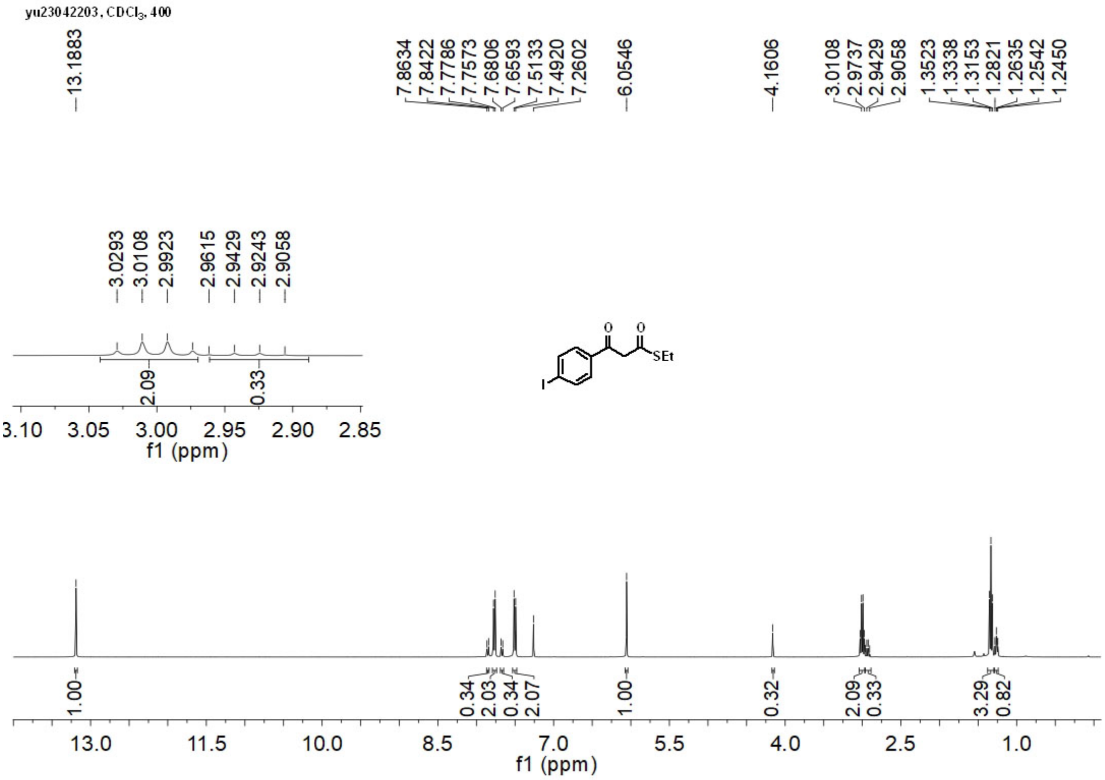

^{1}H NMR of **2i**

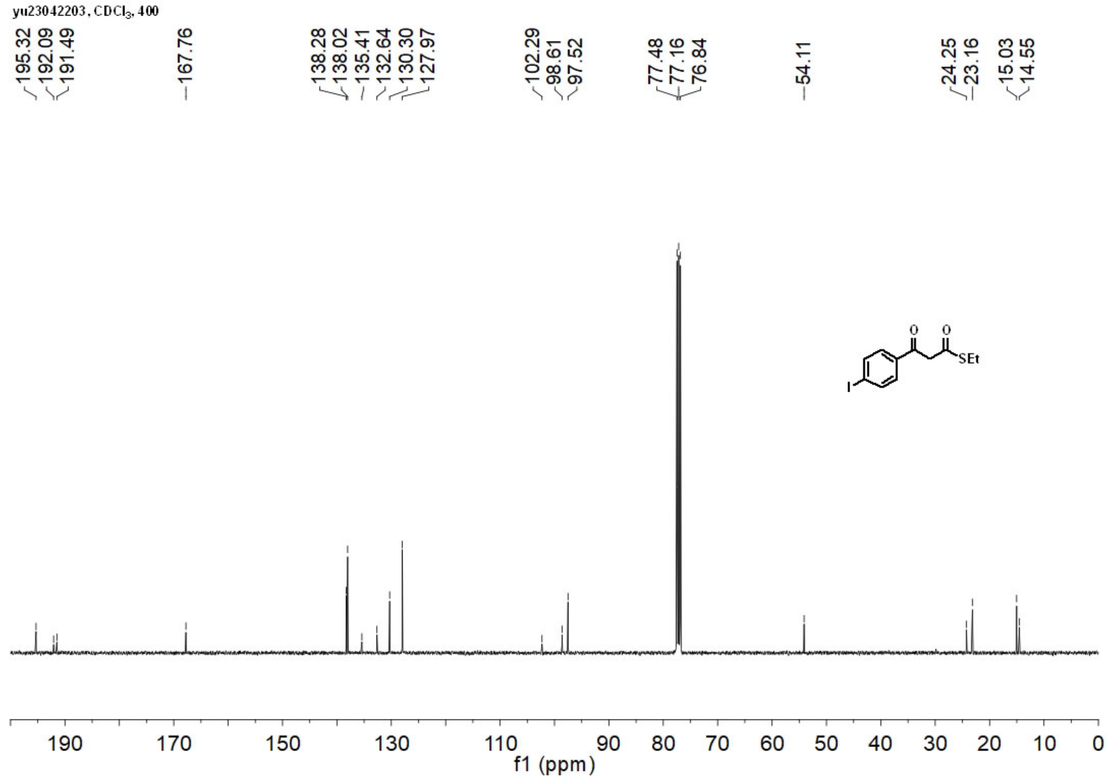

^{13}C NMR of **2i**

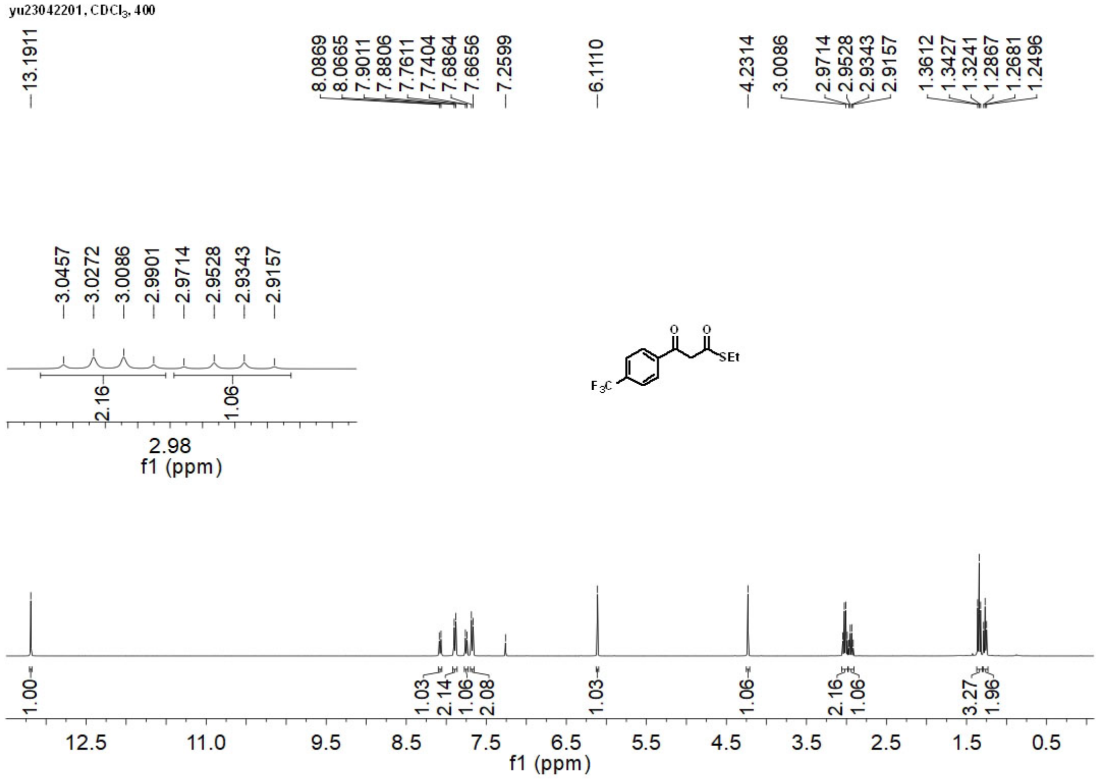

^{1}H NMR of **2j**

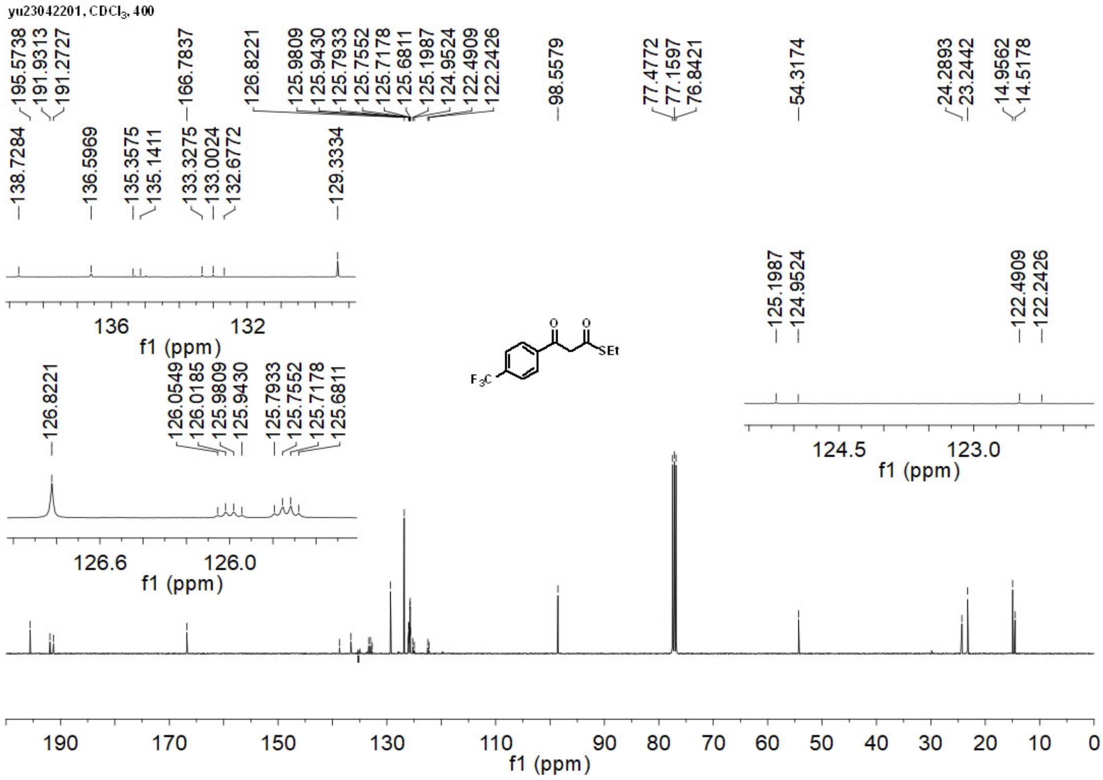

^{13}C NMR of **2j**

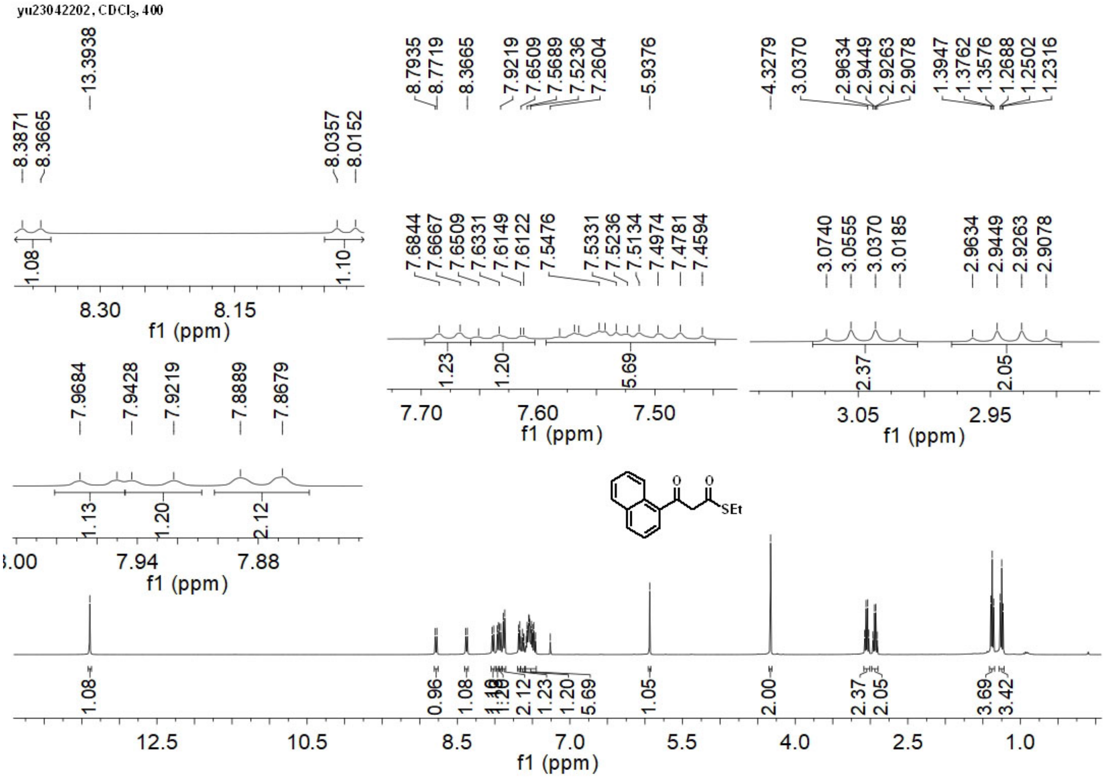

^{1}H NMR of **2k**

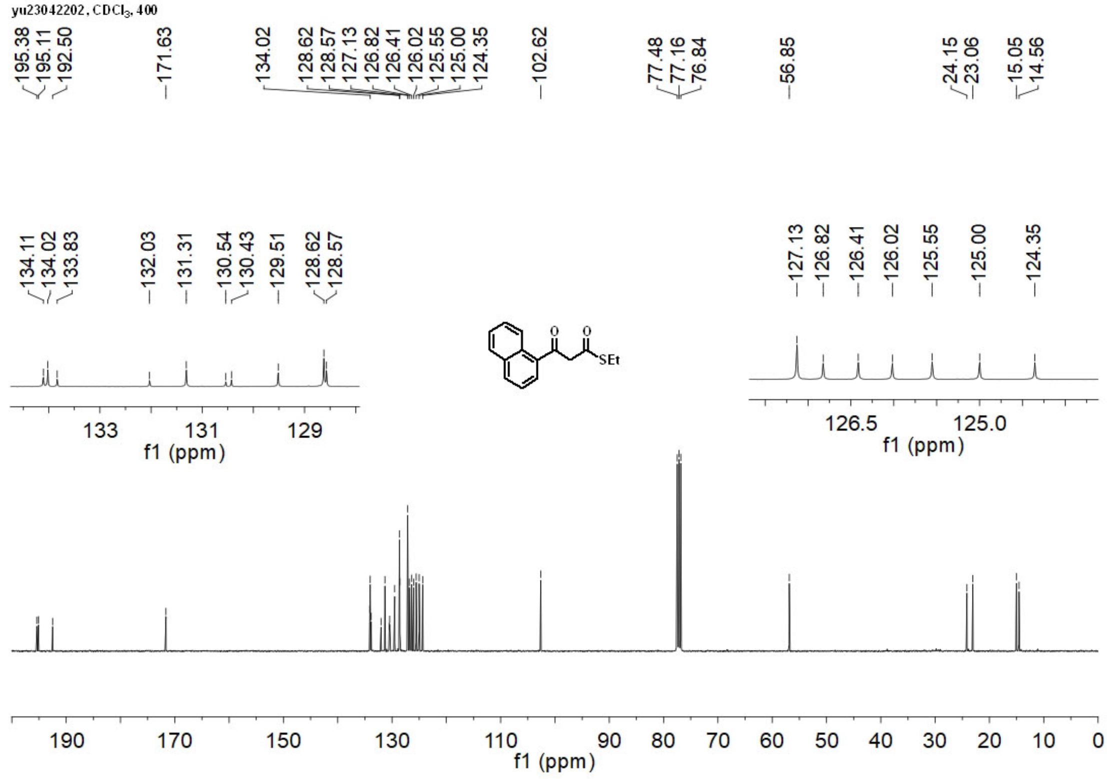

^{13}C NMR of **2k**

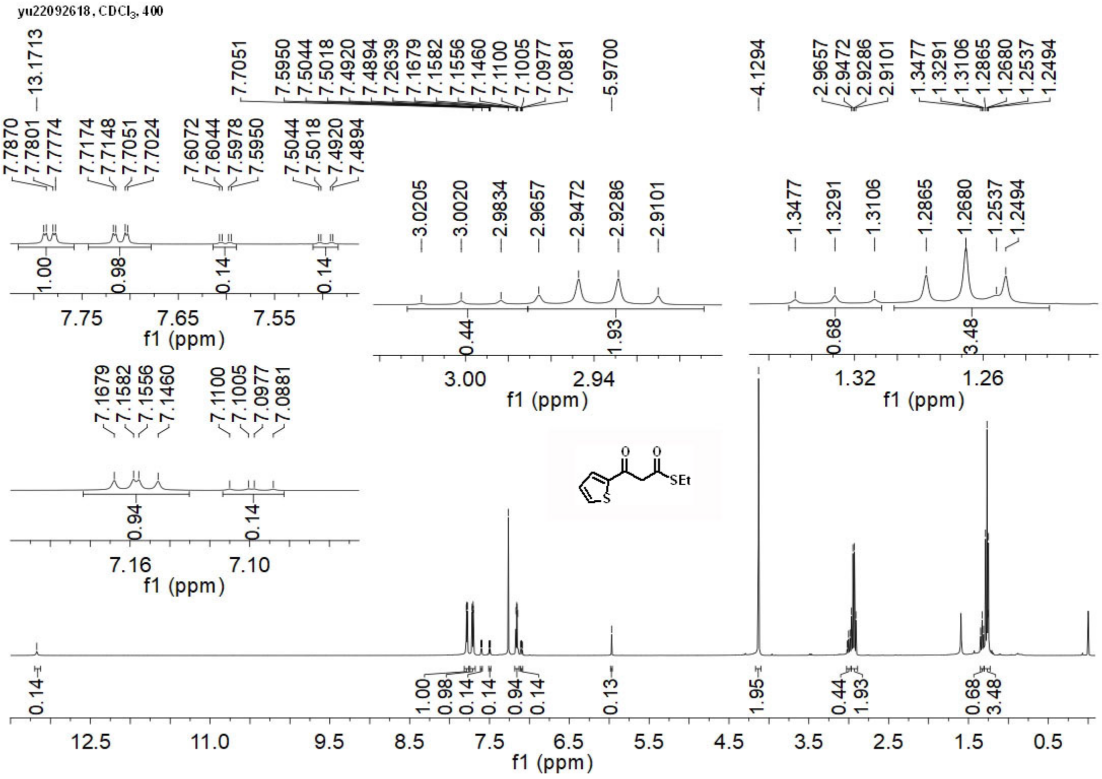

^{1}H NMR of **2l**

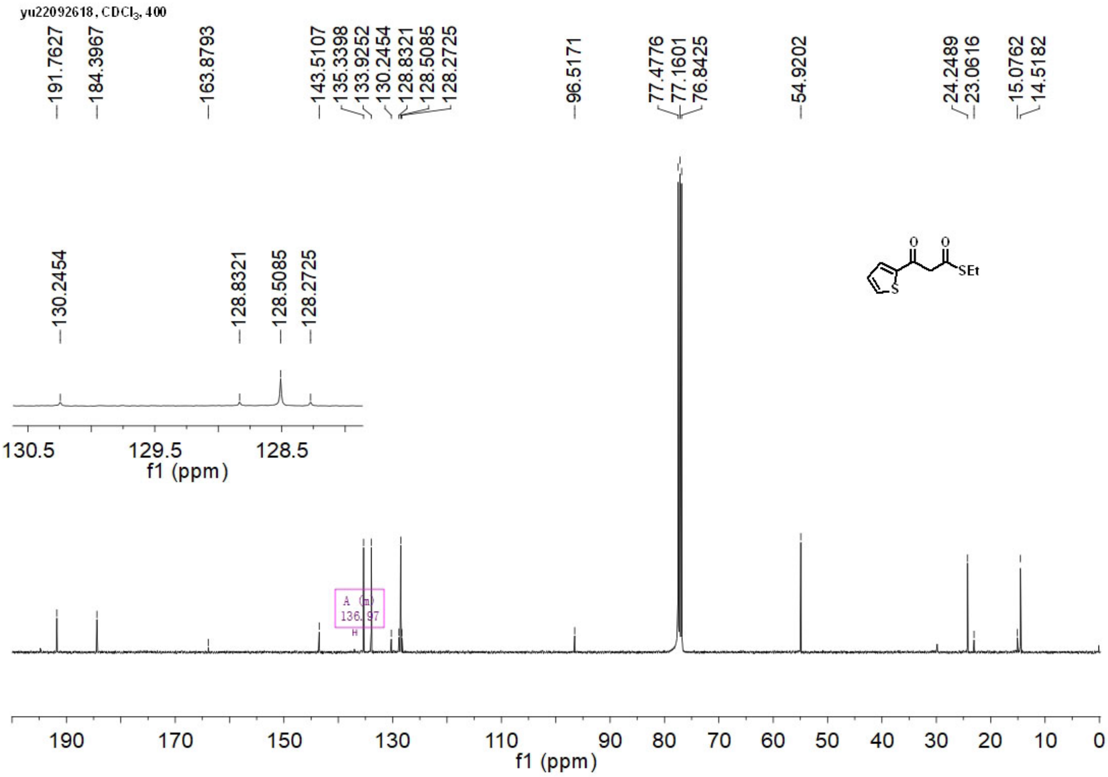

^{13}C NMR of **2l**

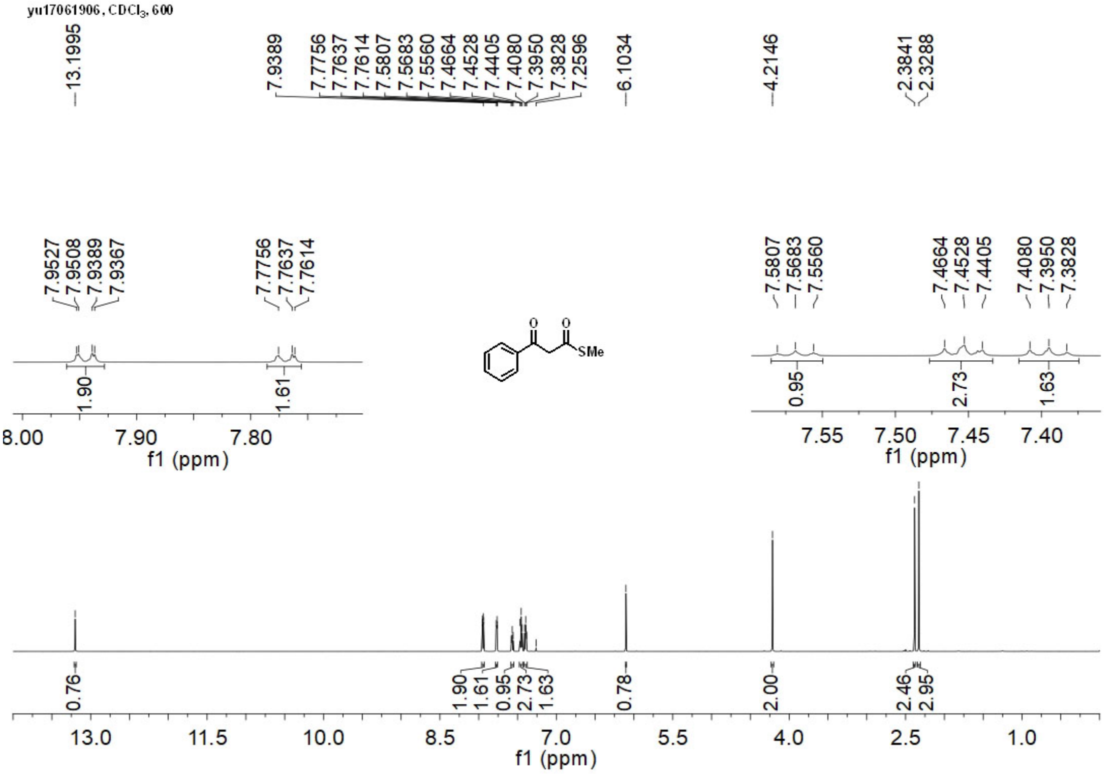

^{1}H NMR of **2m**

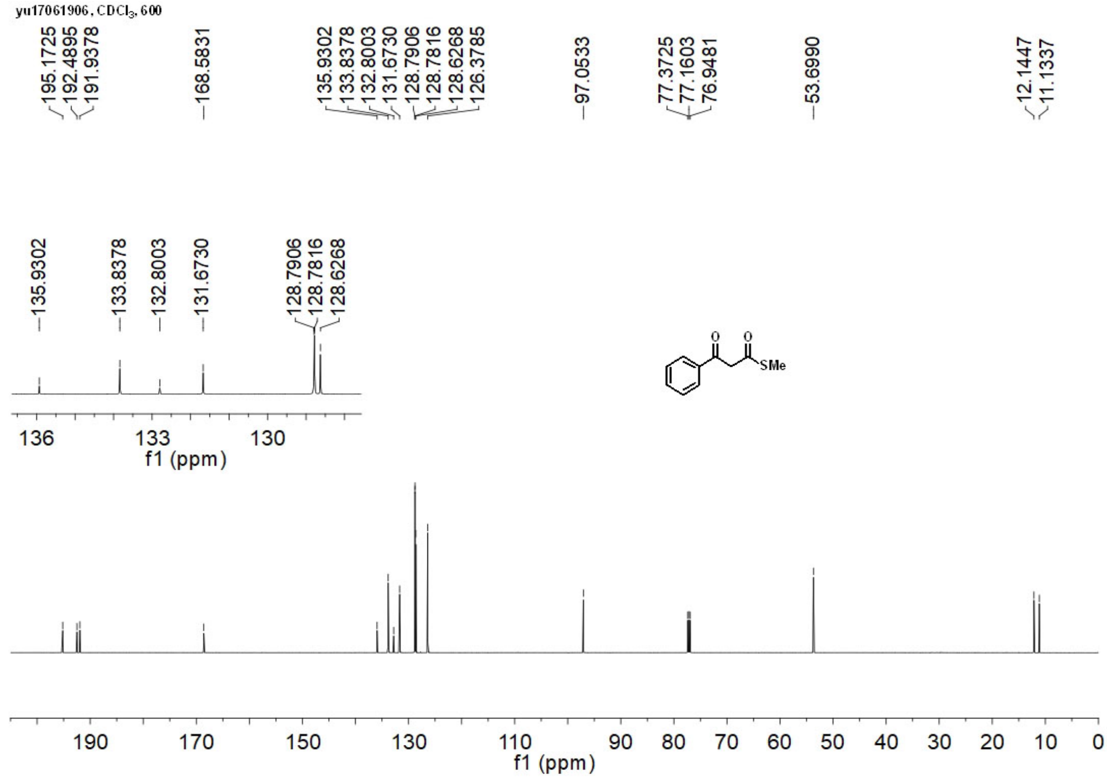

^{13}C NMR of **2m**

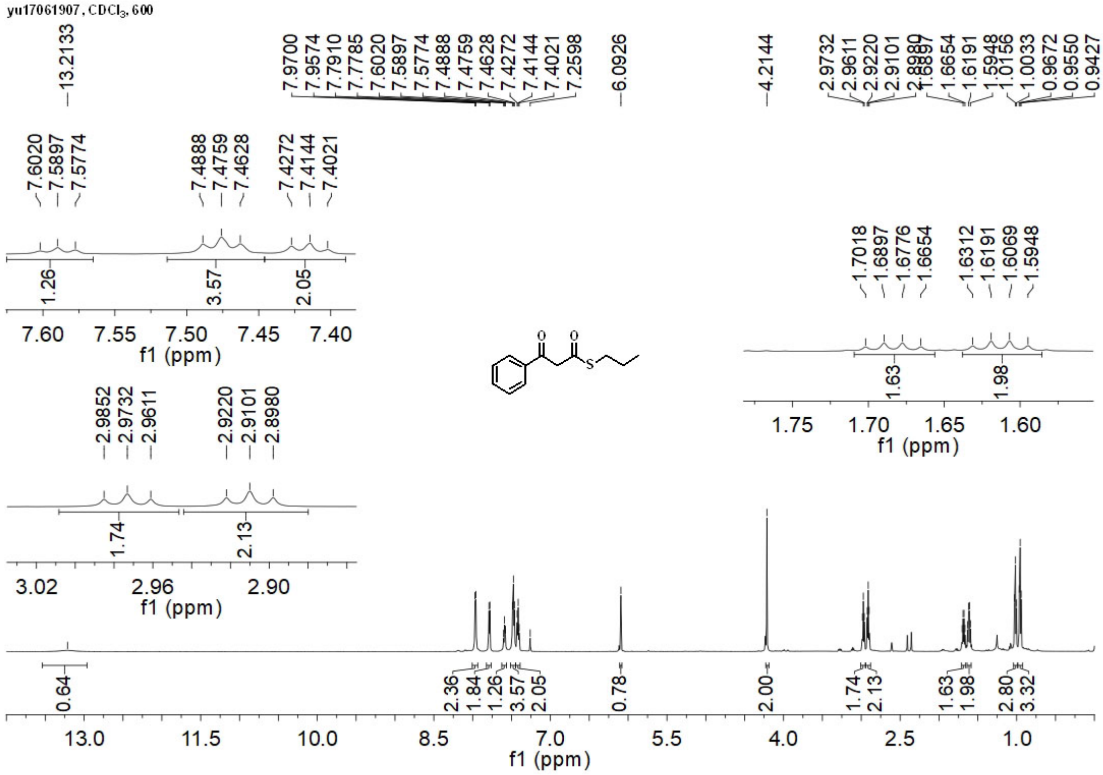

^{1}H NMR of **2n**

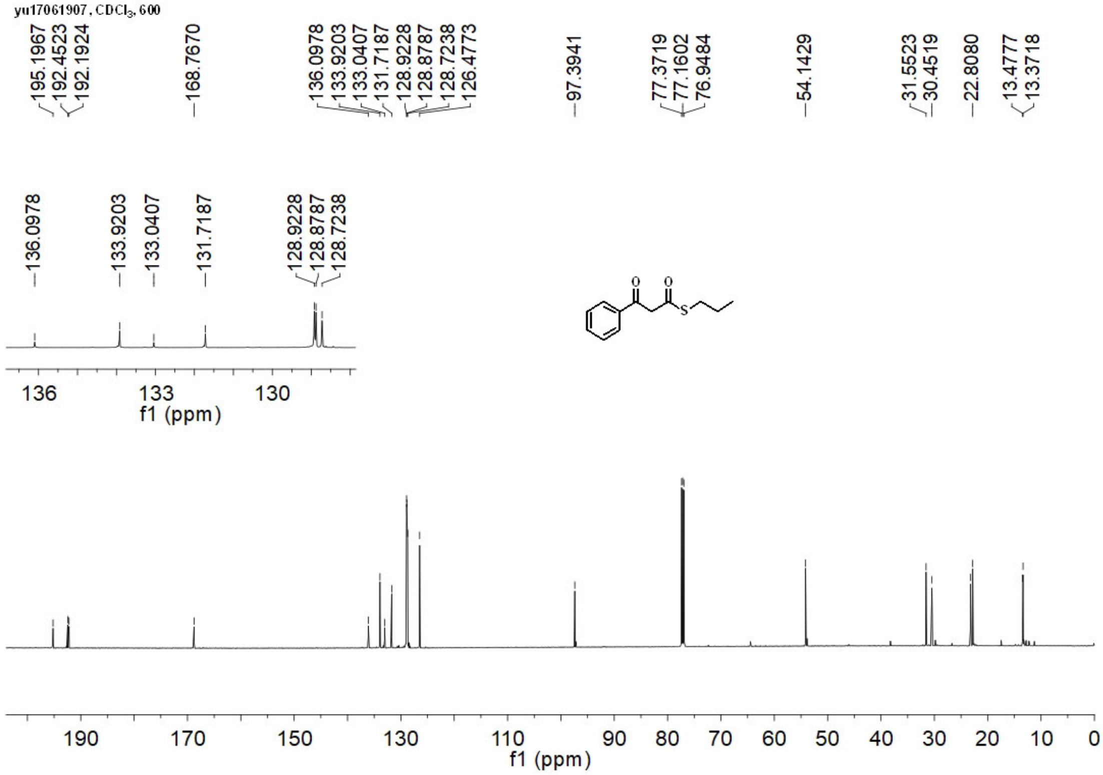

^{13}C NMR of **2n**

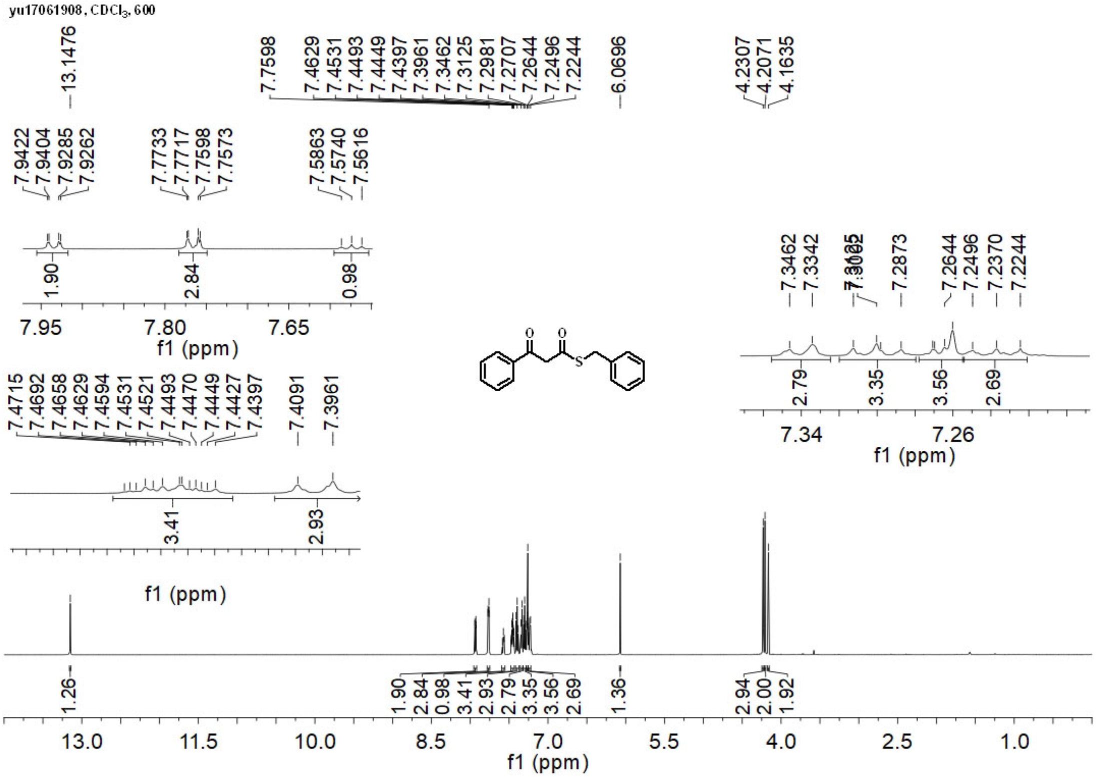

^{1}H NMR of 2**o**

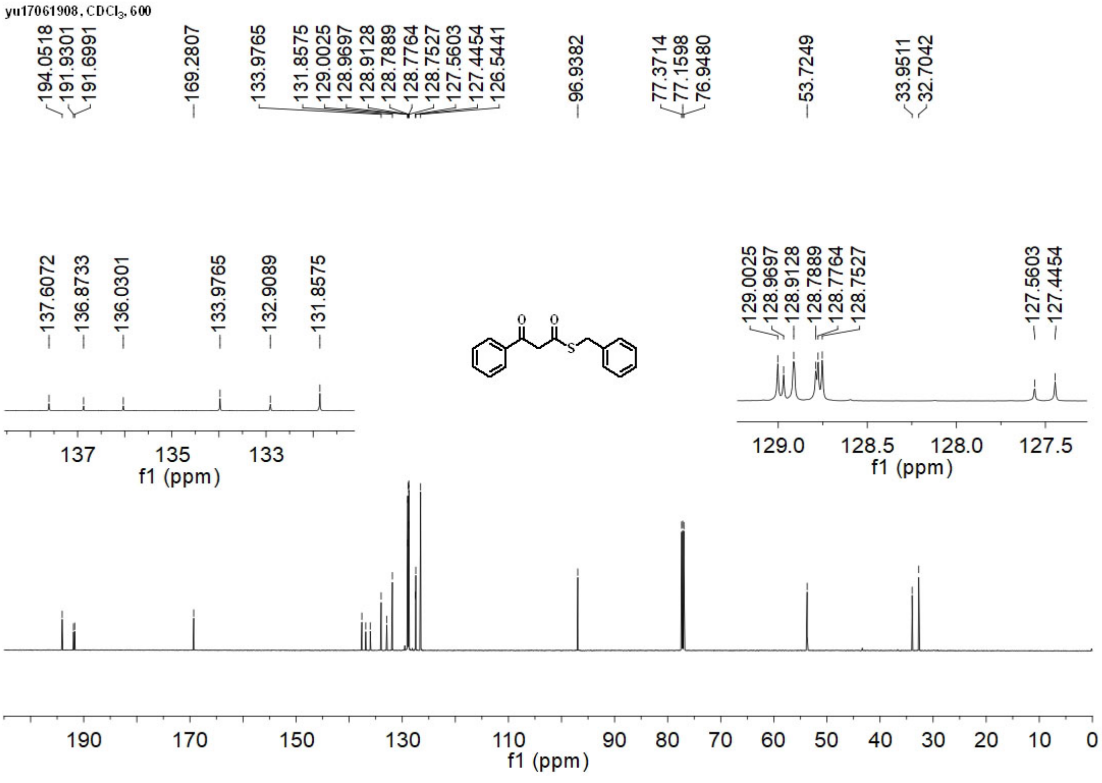

^{13}C NMR of **2o**

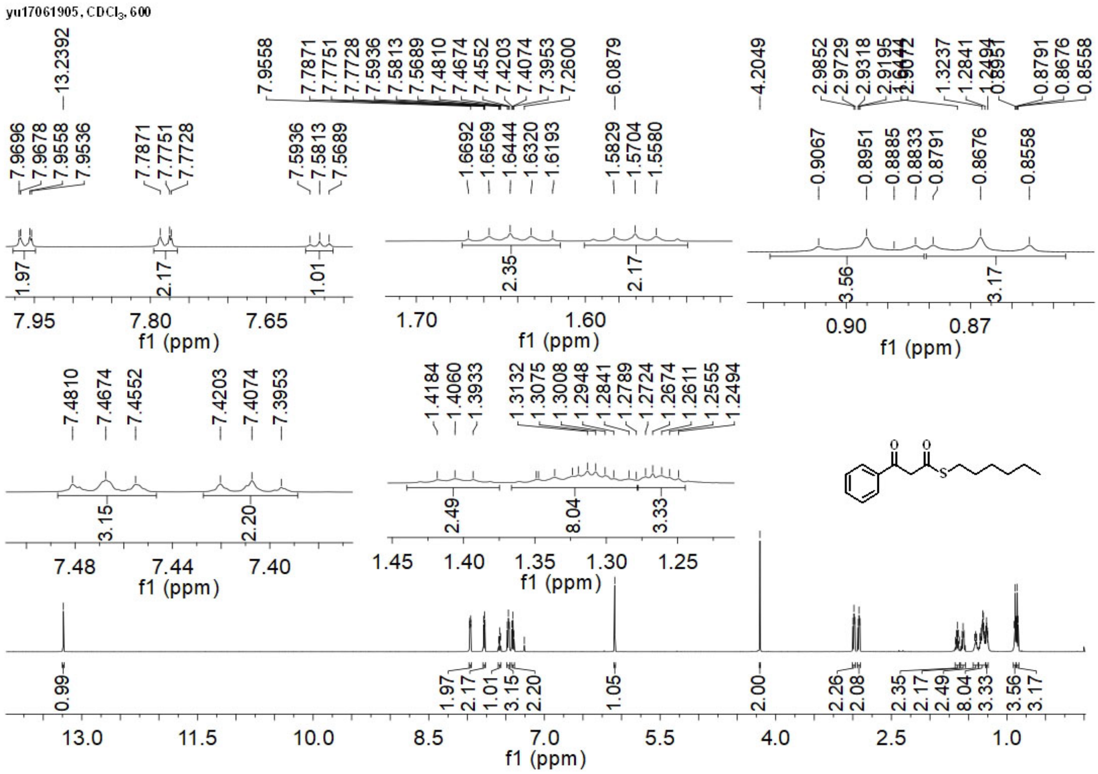

^{1}H NMR of **2p**

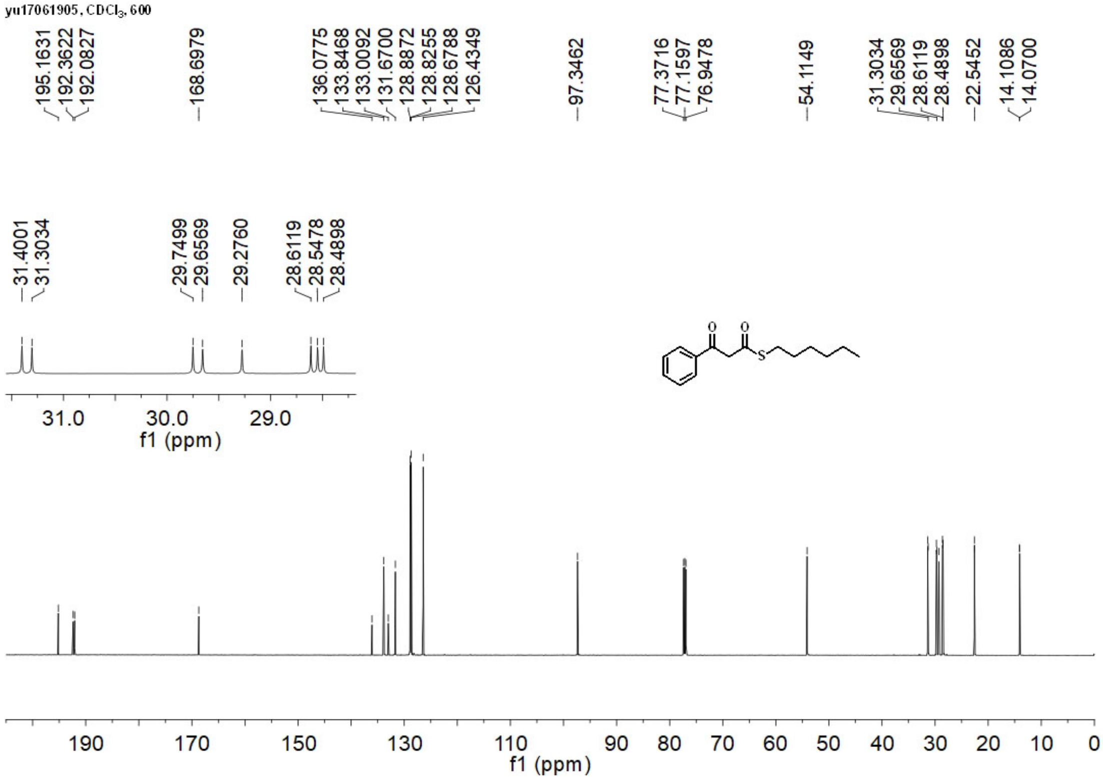

^{13}C NMR of **2p**

5. Copies of ^{1}H NMR and ^{13}C NMR spectra of compound 3

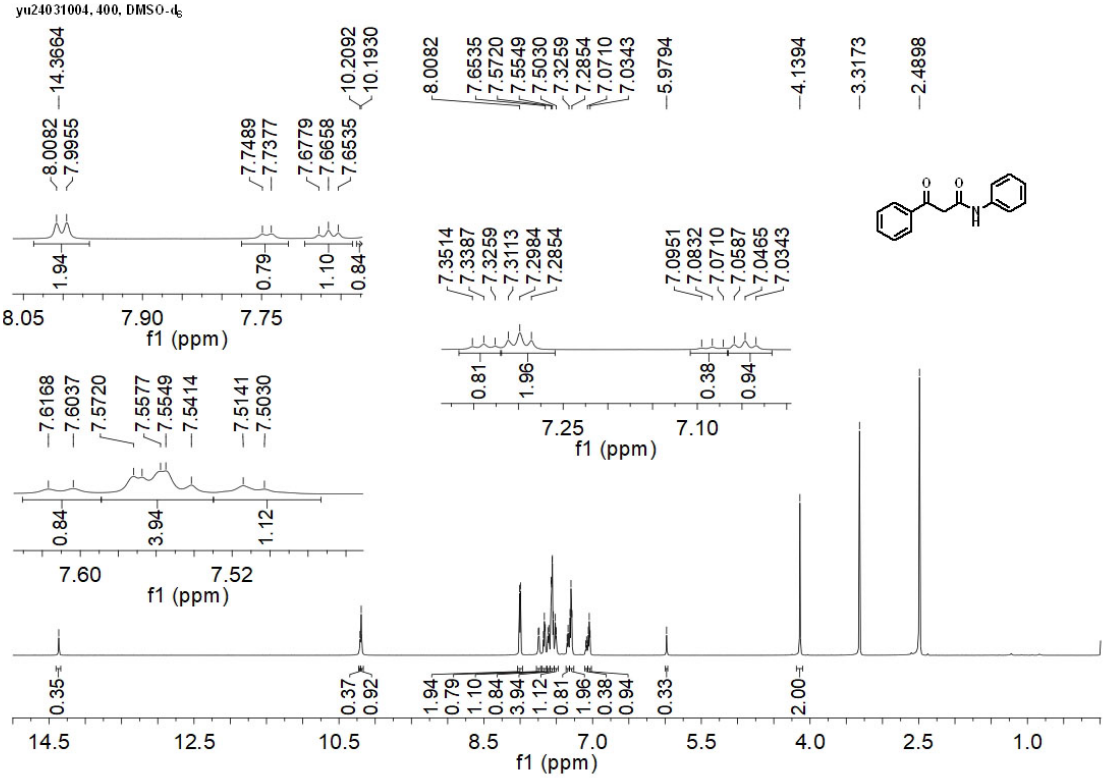

^{1}H NMR of **3a**

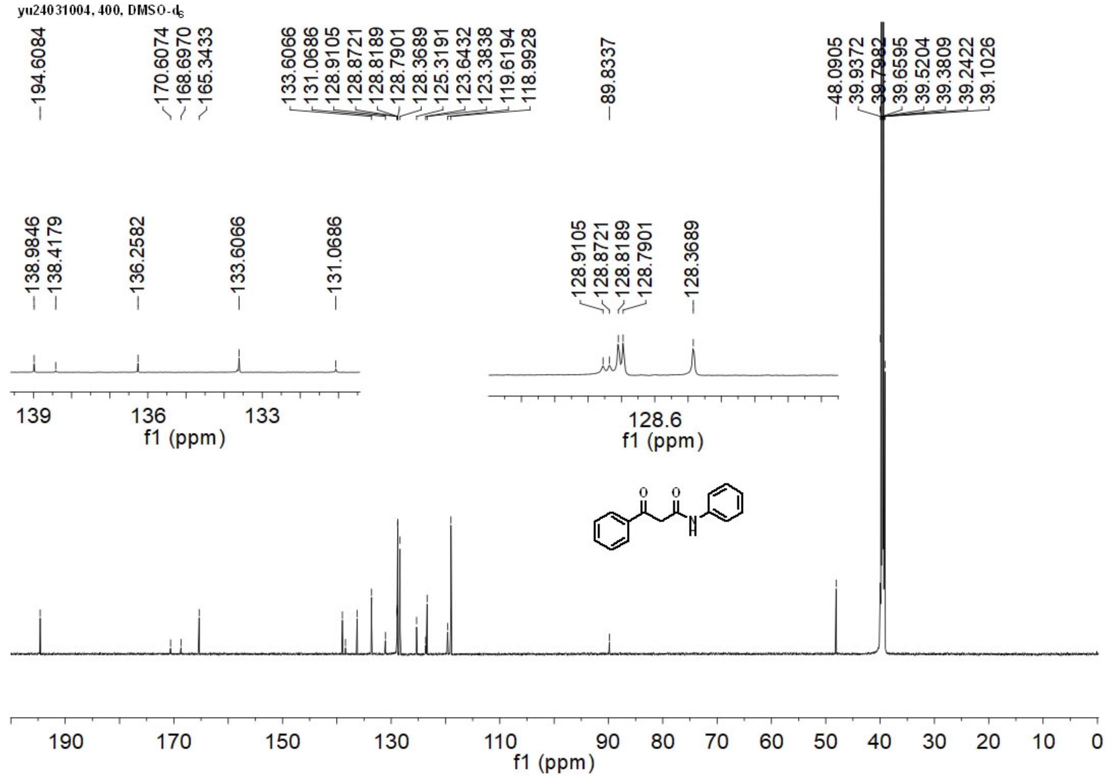

^{13}C NMR of **3a**

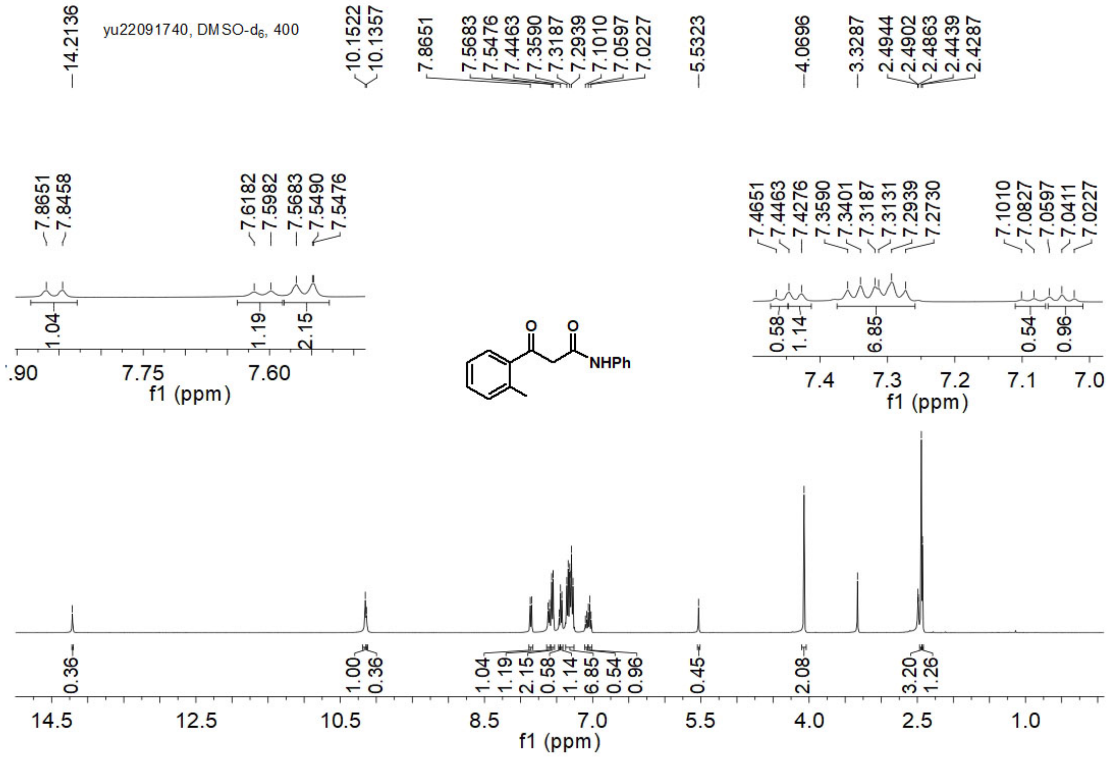

^{1}H NMR of **3b**

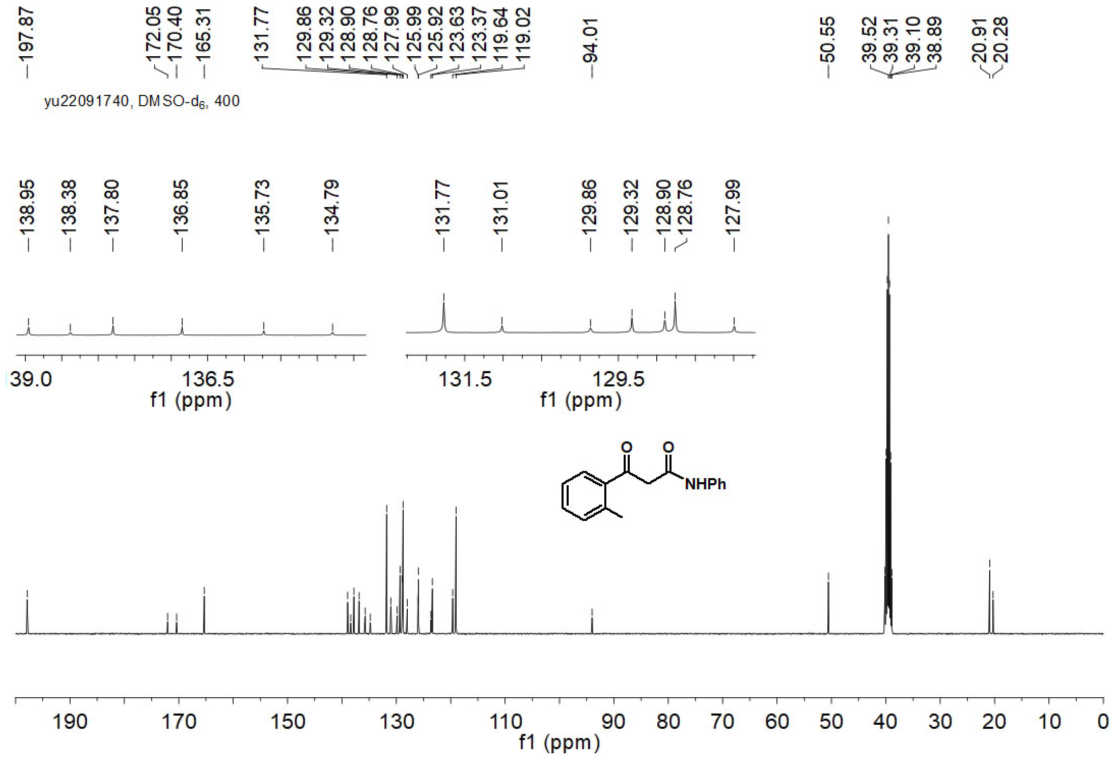

^{13}C NMR of **3b**

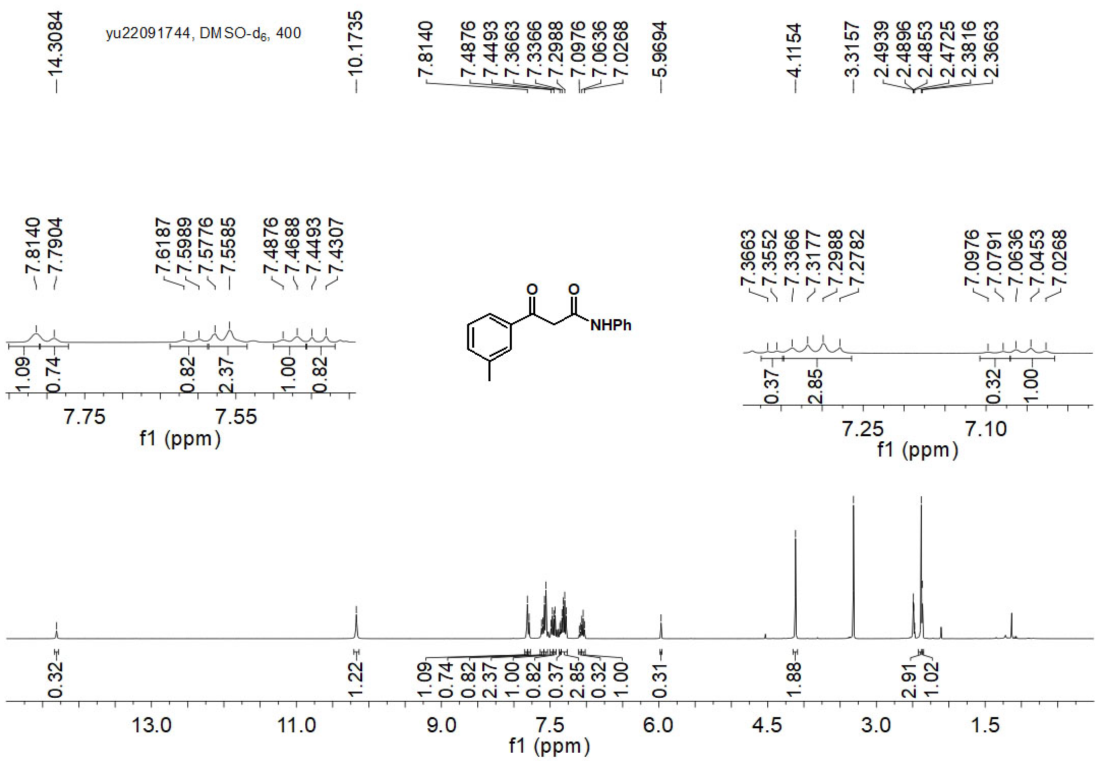

^{1}H NMR of **3c**

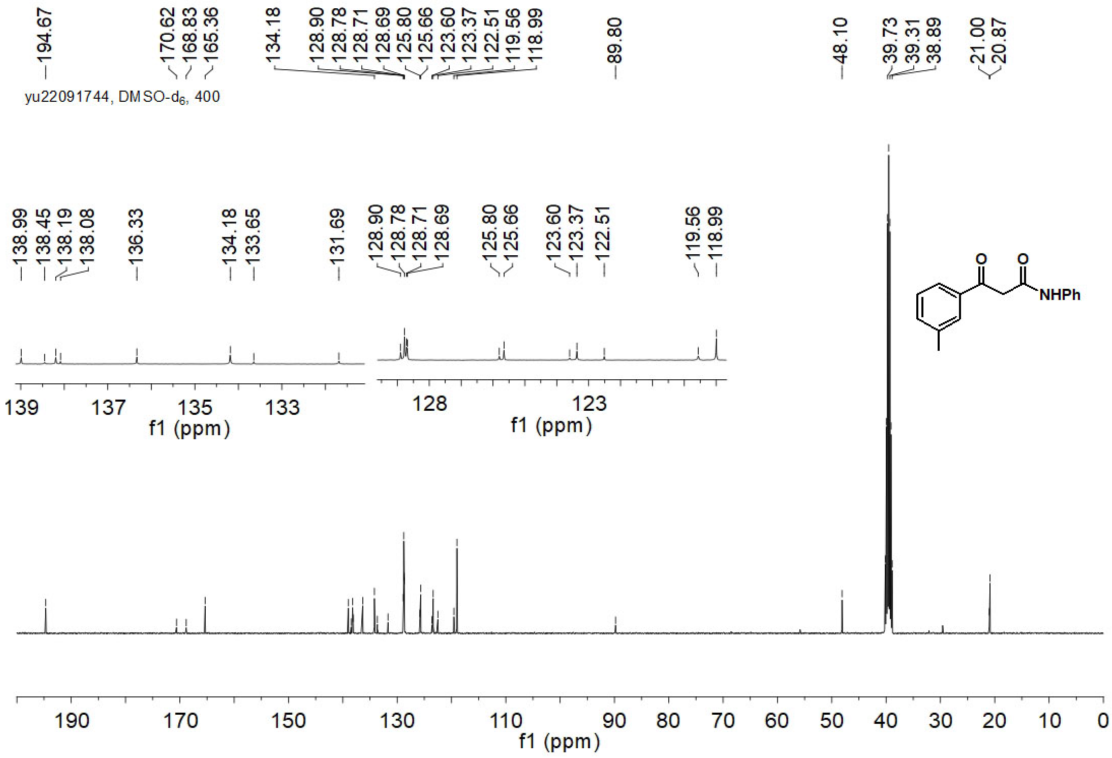

^{13}C NMR of **3c**

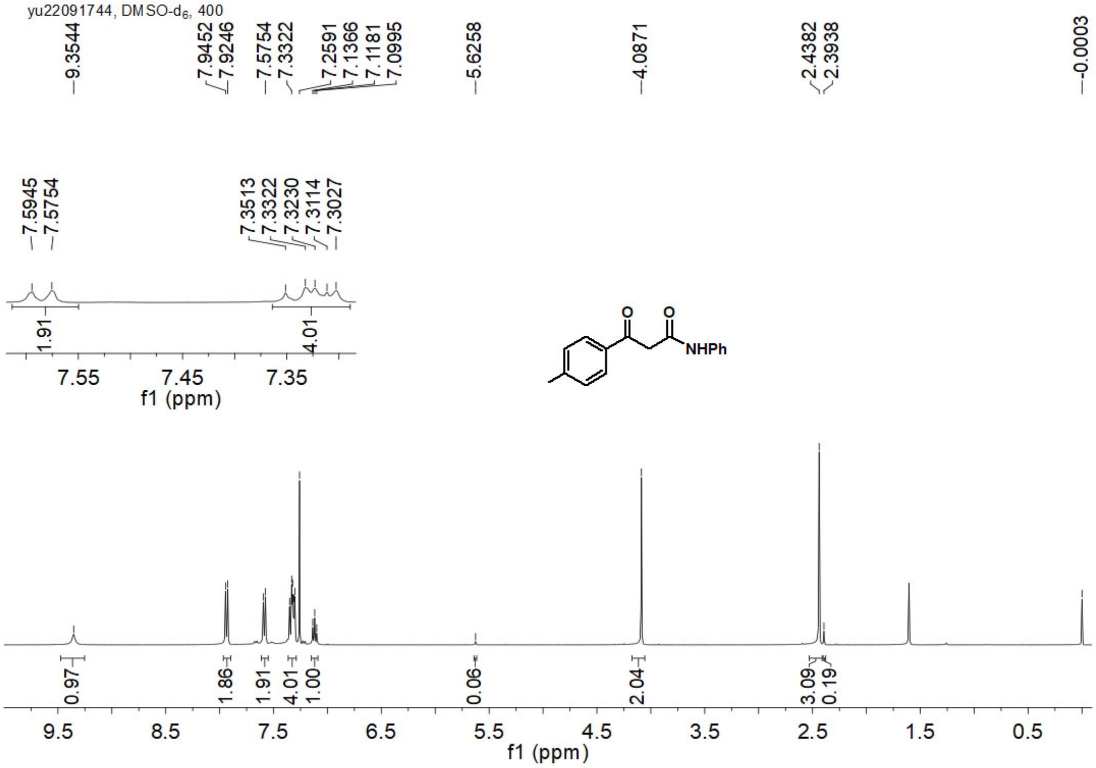

^{1}H NMR of **3d**

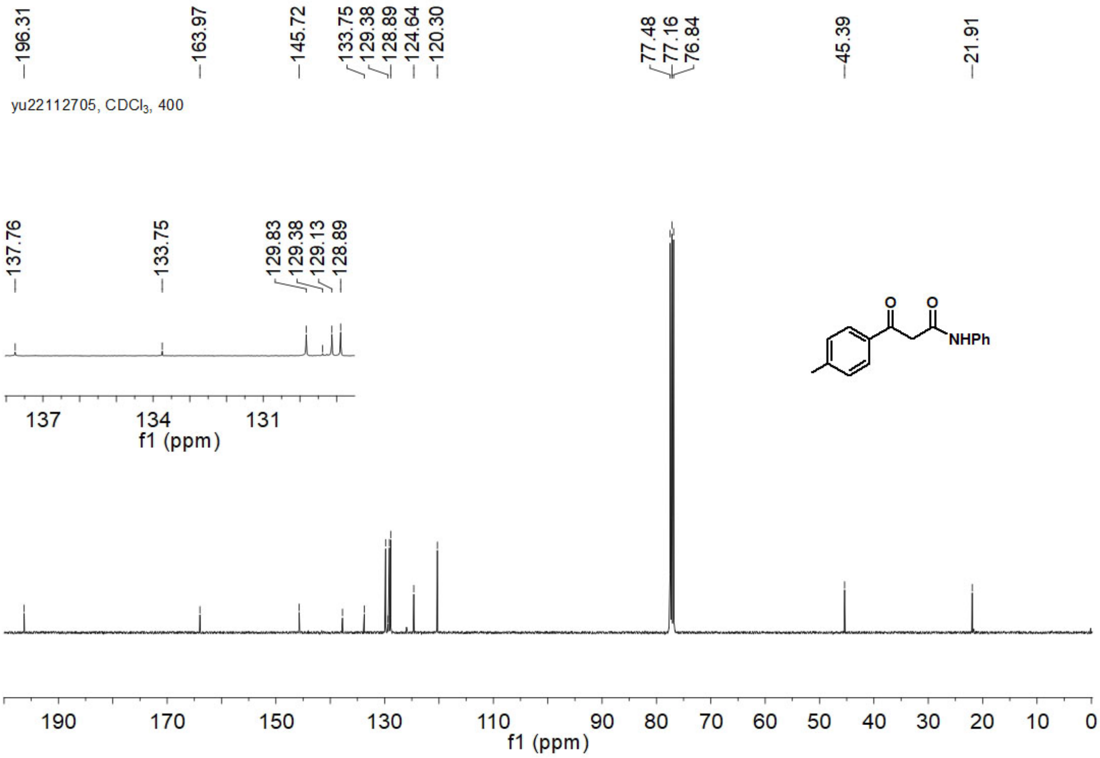

^{13}C NMR of **3d**

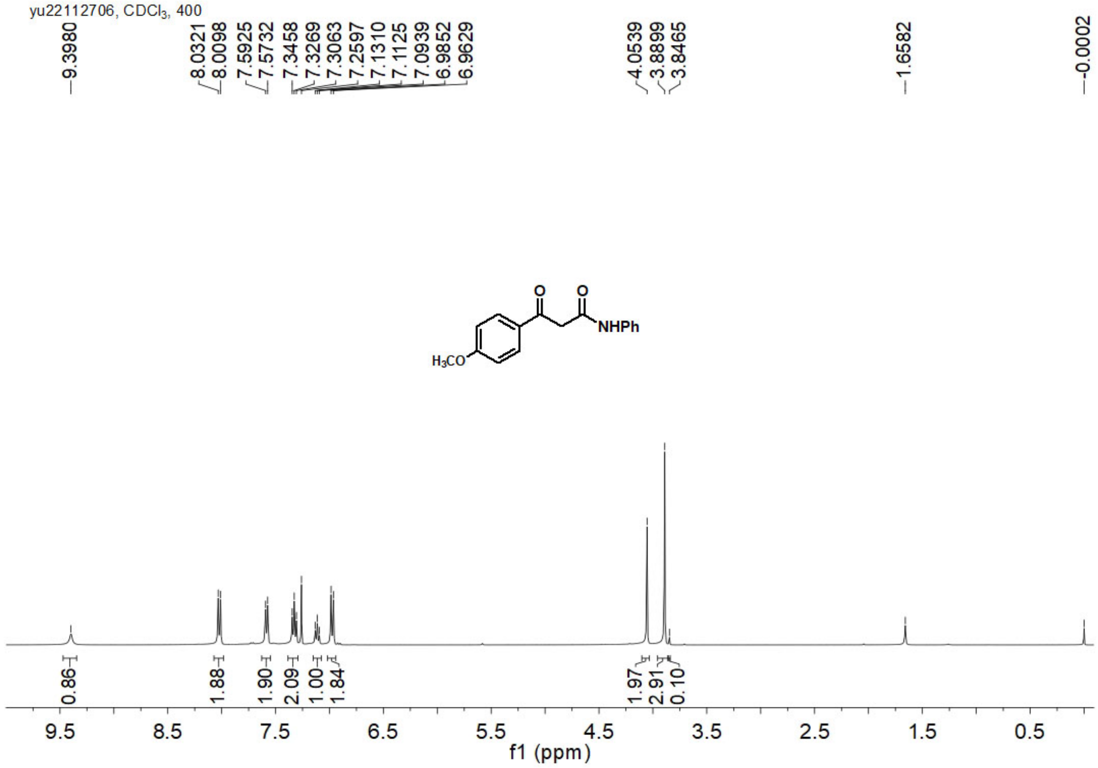

^{1}H NMR of **3e**

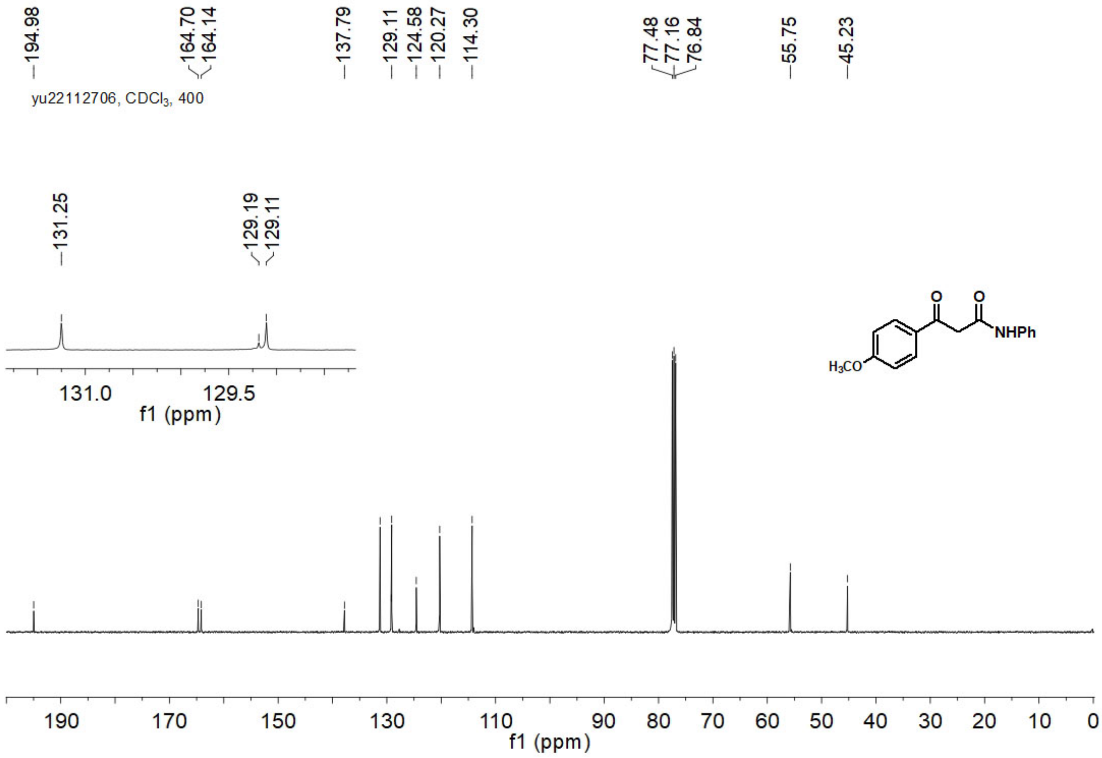

^{13}C NMR of **3e**

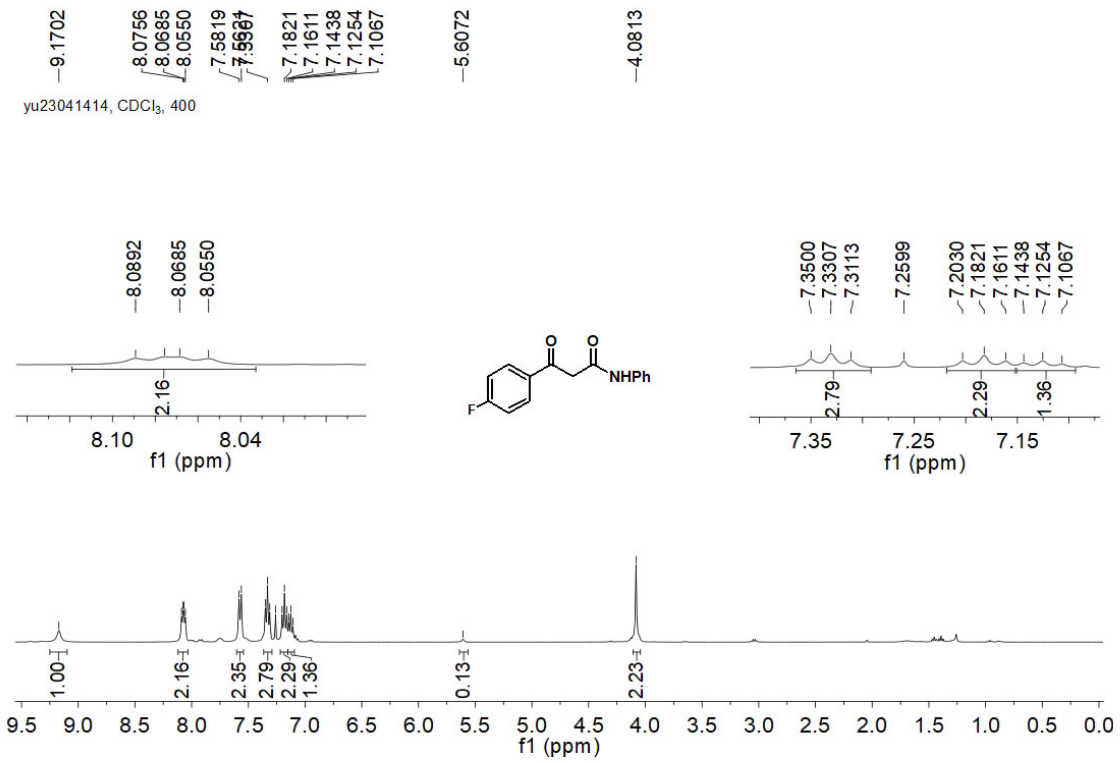

^{1}H NMR of **3f**

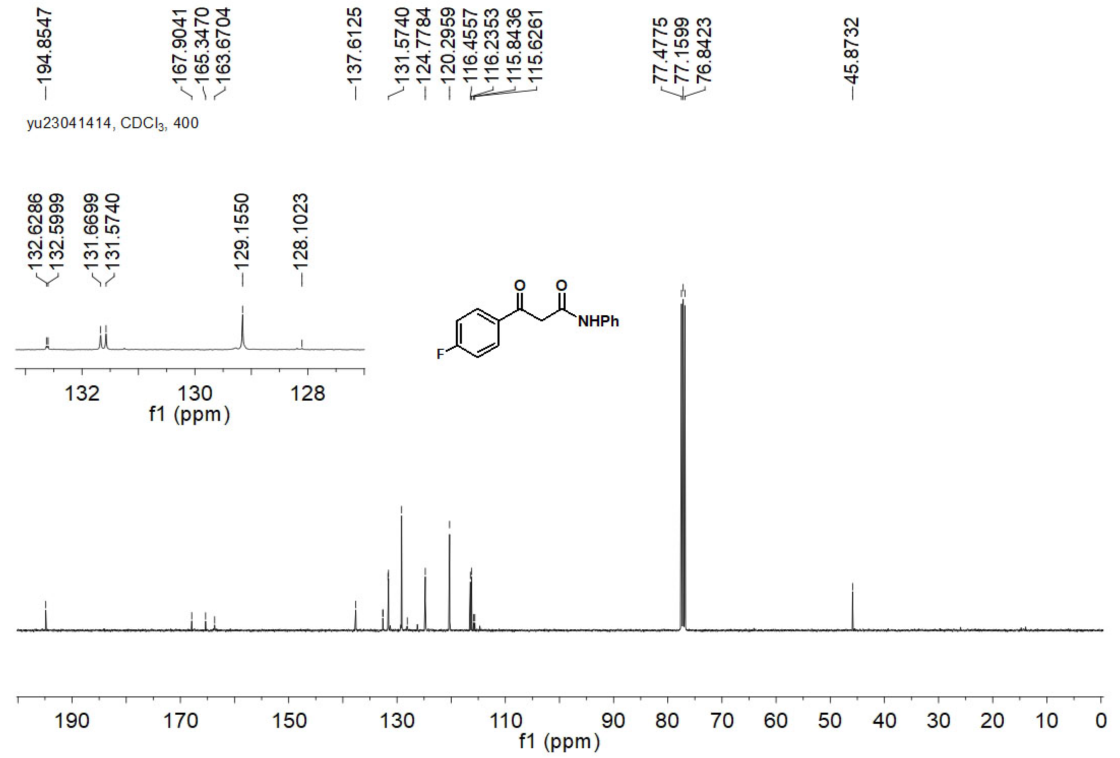

^{13}C NMR of **3f**

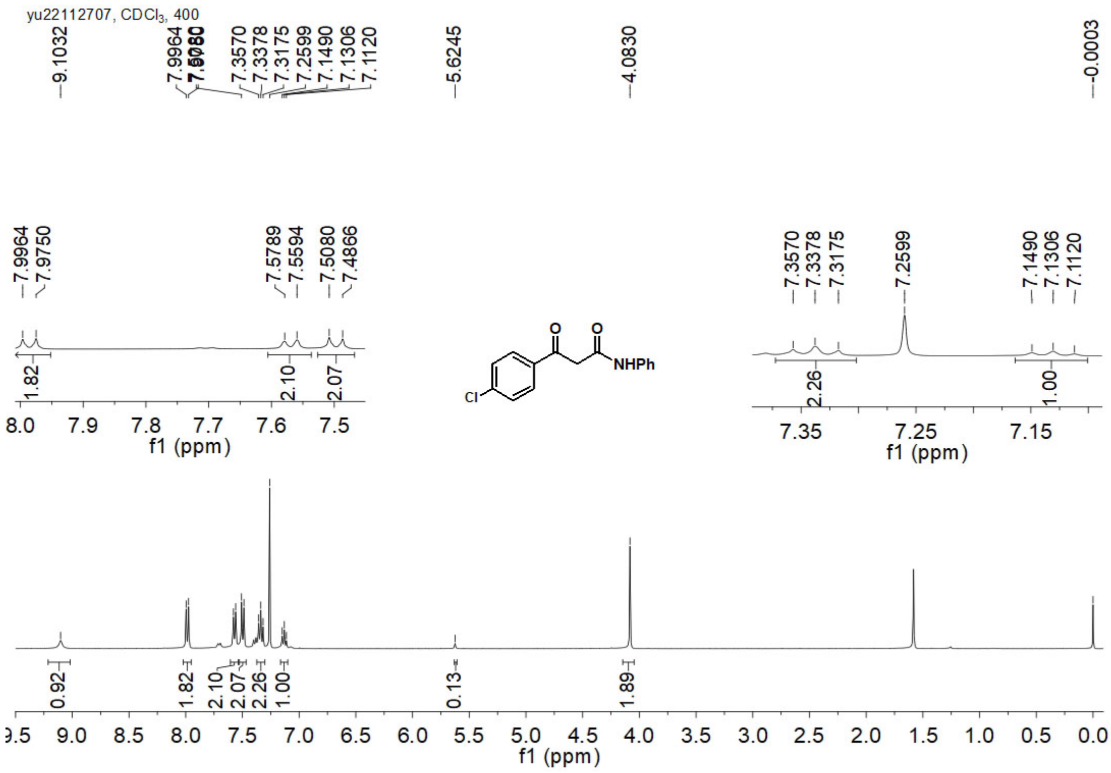

^{1}H NMR of **3g**

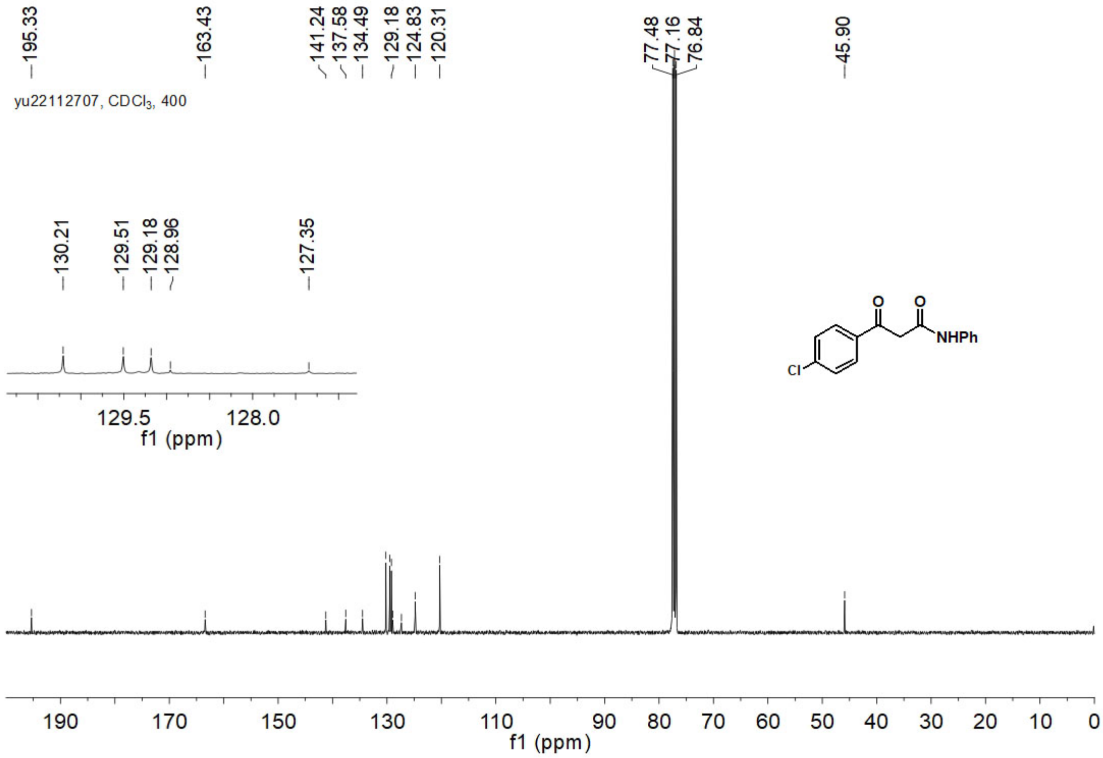

^{13}C NMR of **3g**

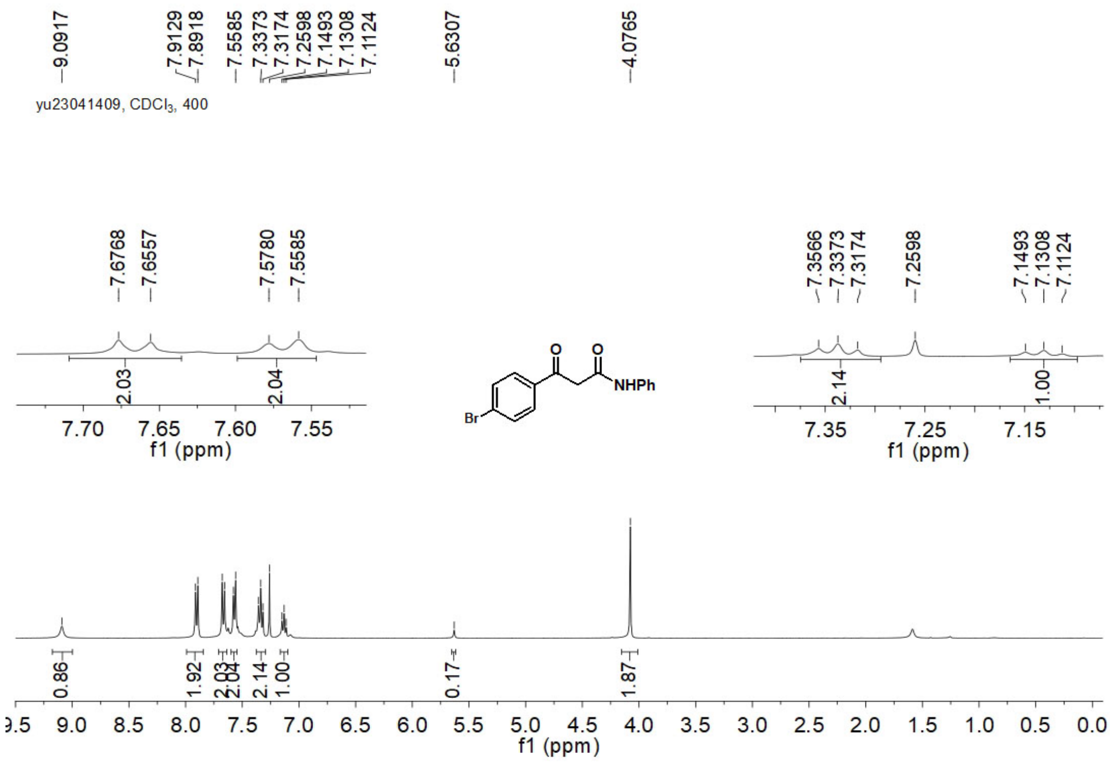

^{1}H NMR of **3h**

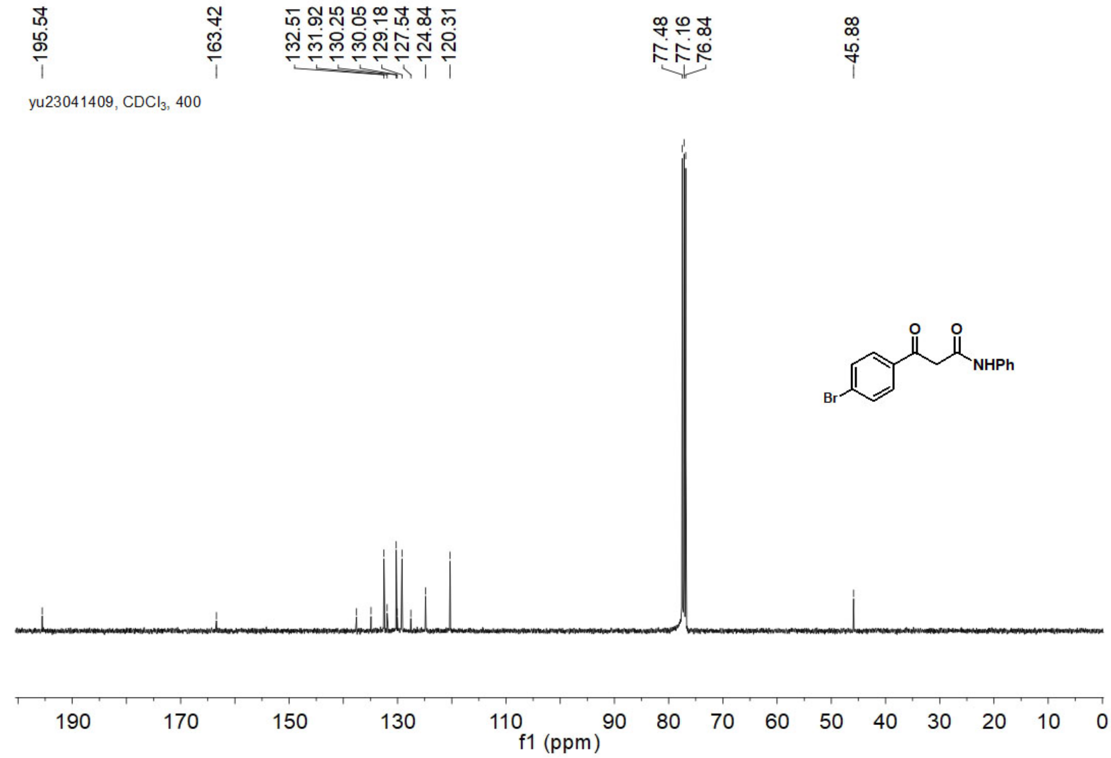

^{13}C NMR of **3h**

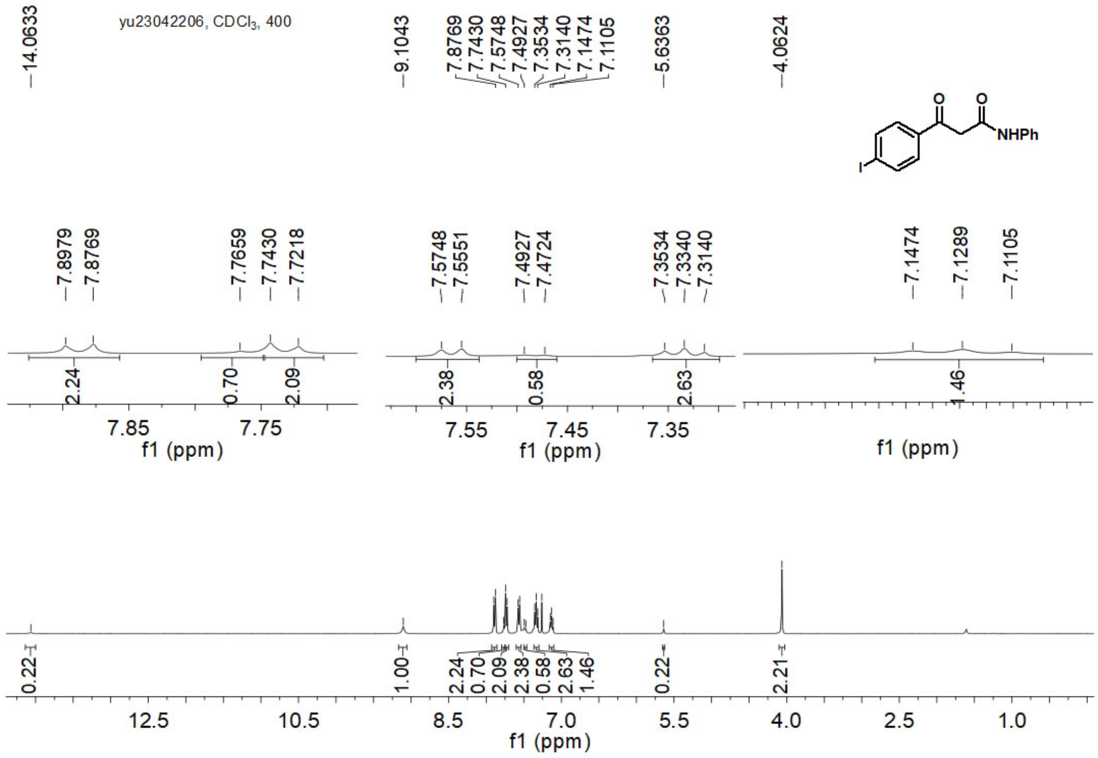

^{1}H NMR of **3i**

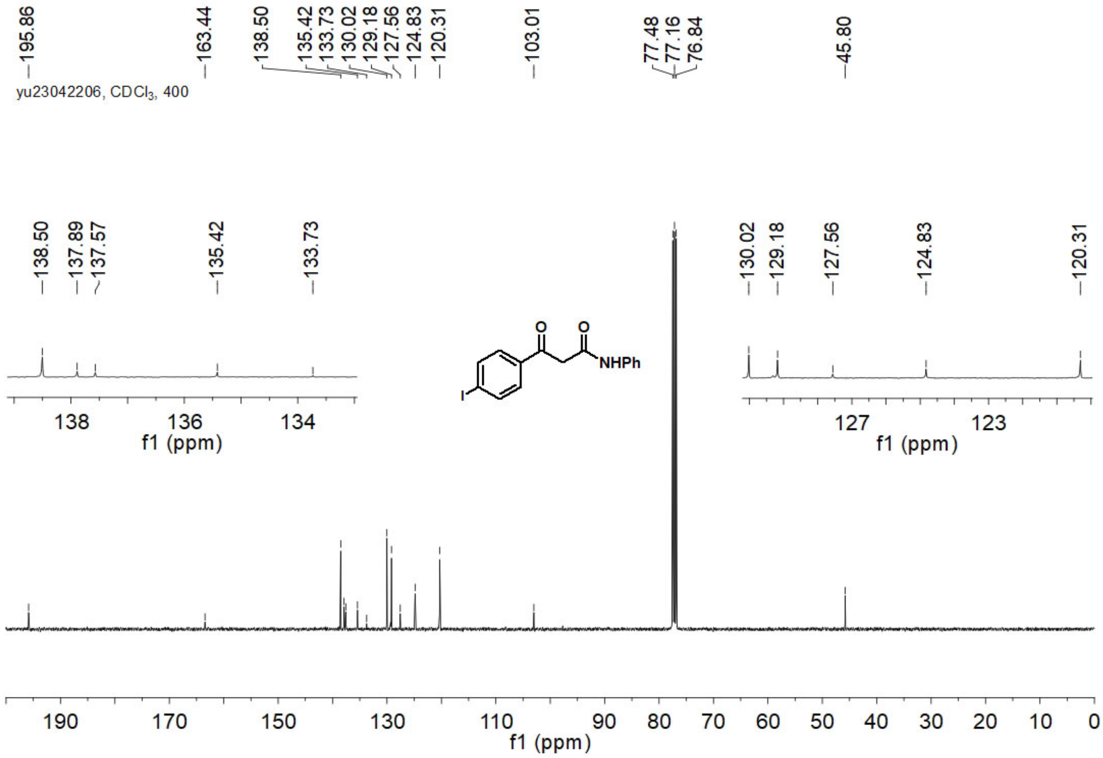

^{13}C NMR of **3i**

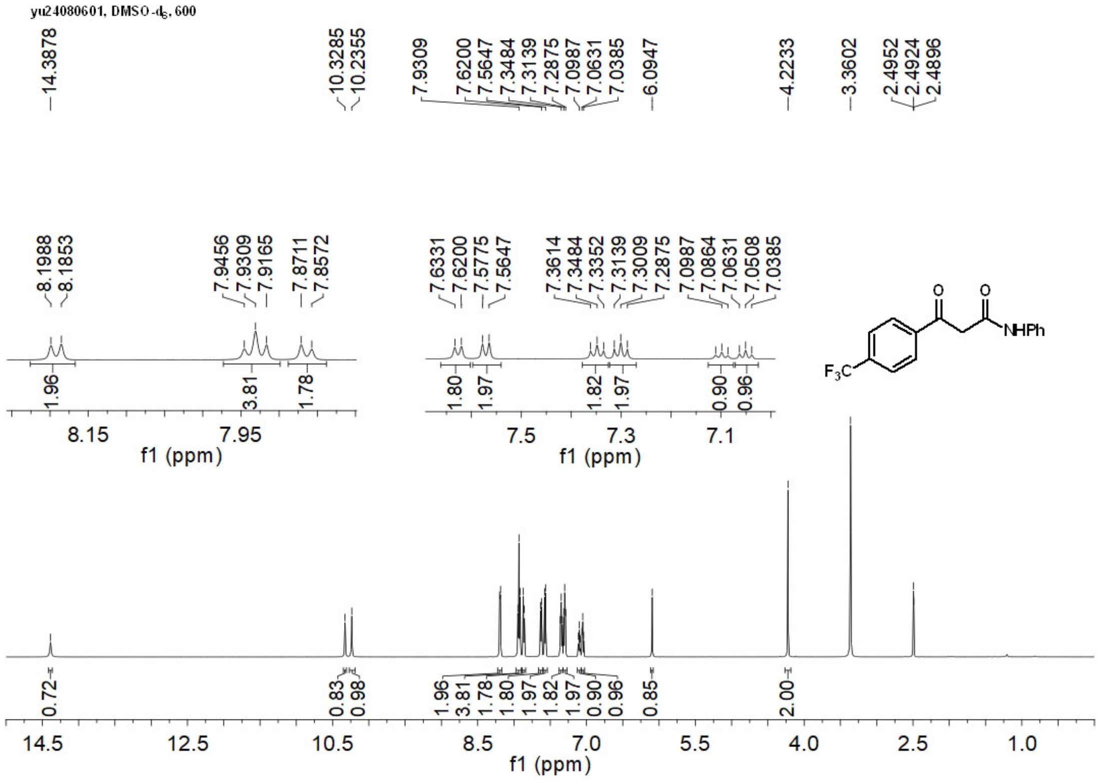

^{1}H NMR of **3j**

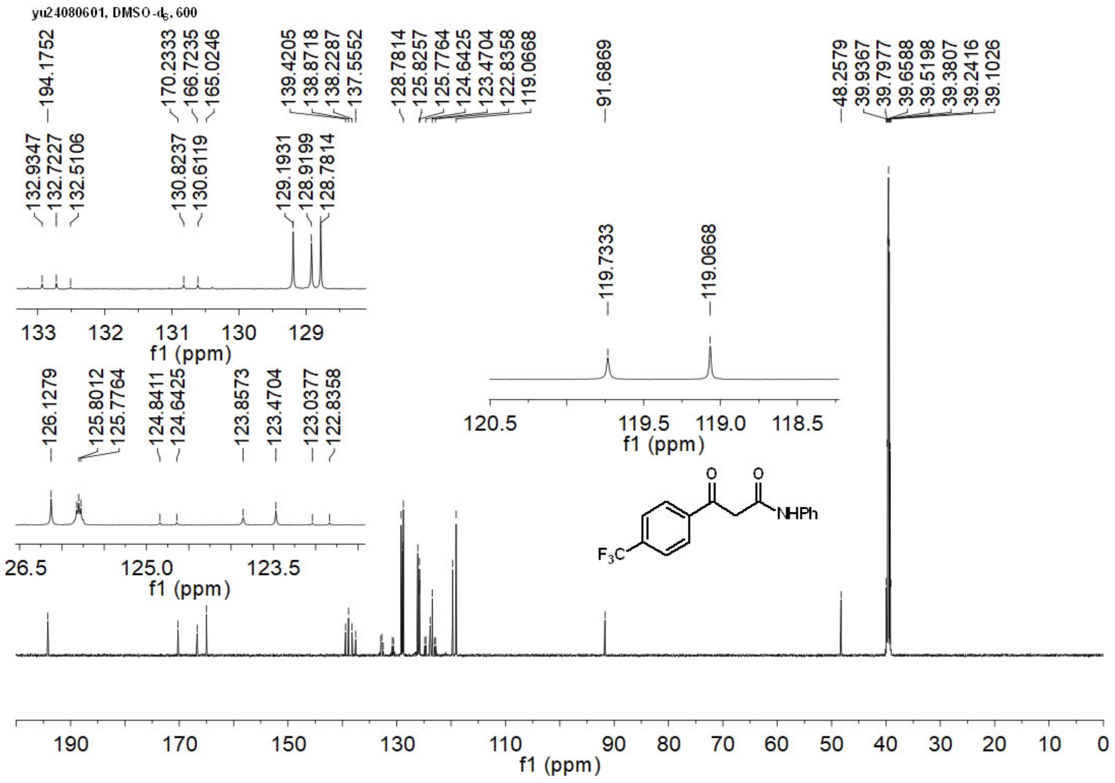

^{13}C NMR of **3j**

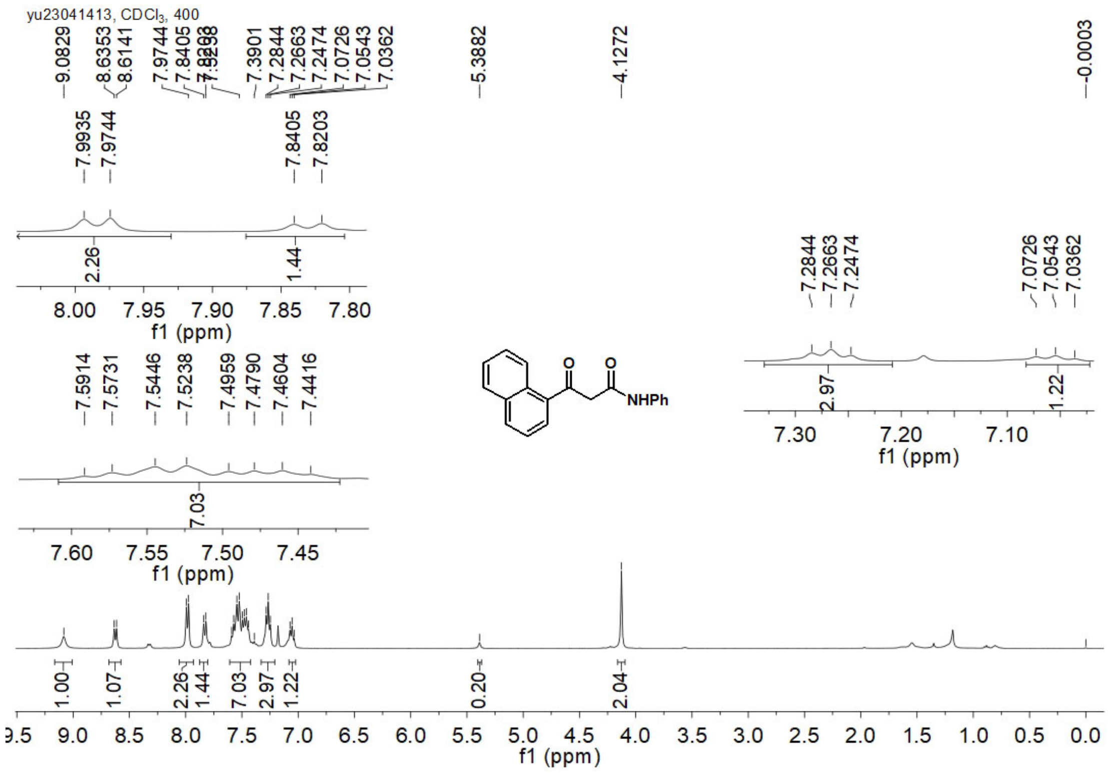

^{1}H NMR of **3k**

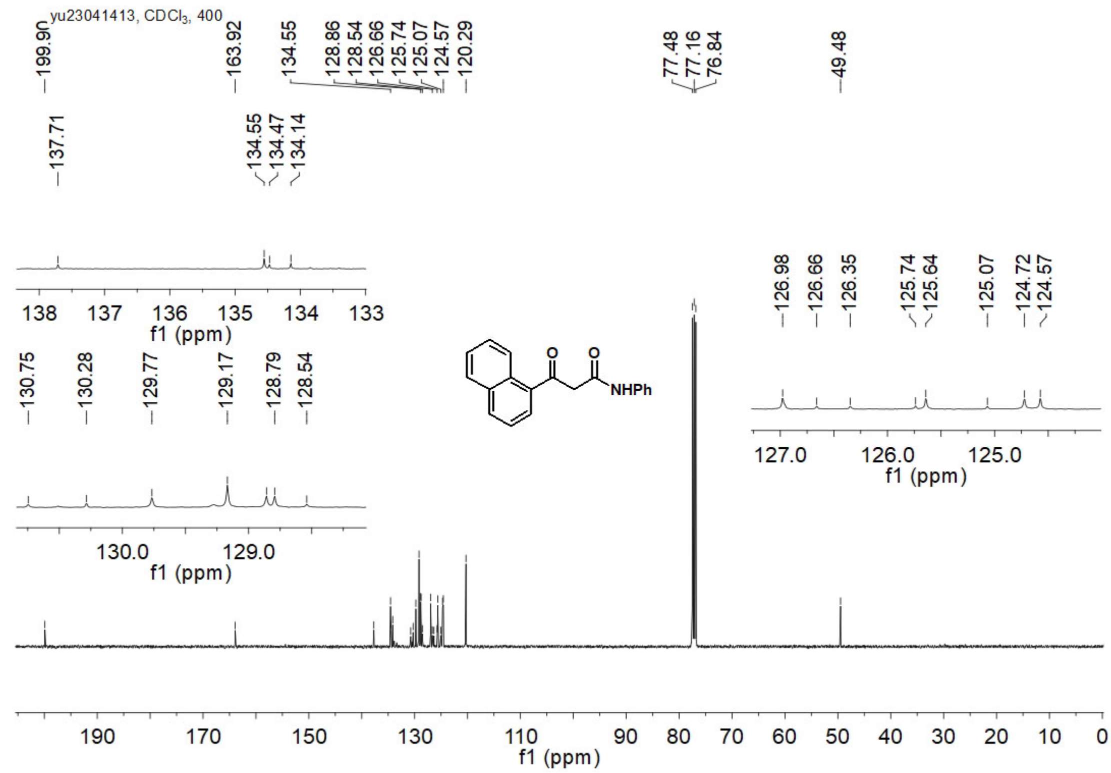

^{13}C NMR of **3k**

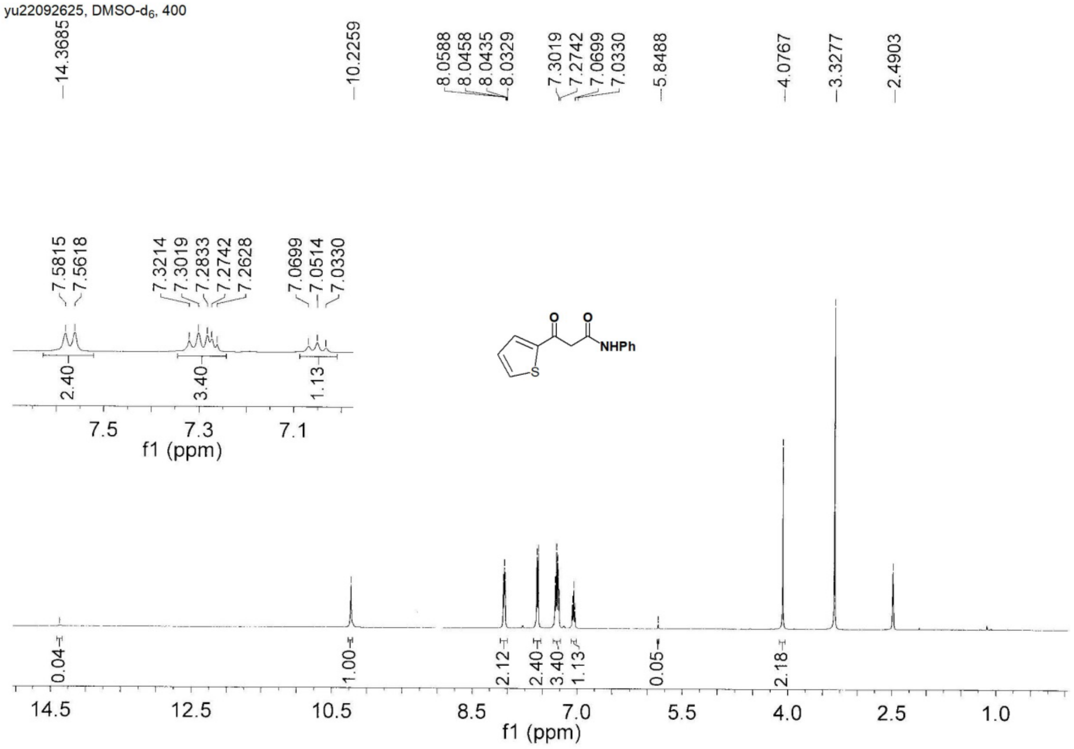

^{1}H NMR of **3l**

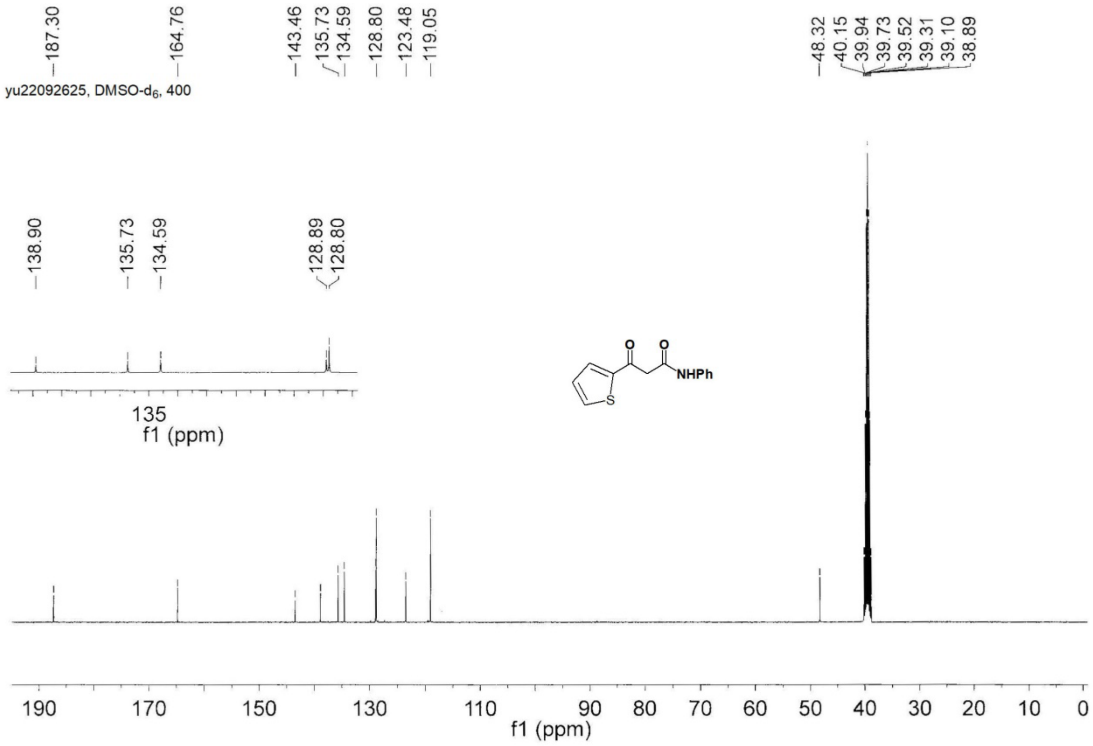

^{13}C NMR of **3l**

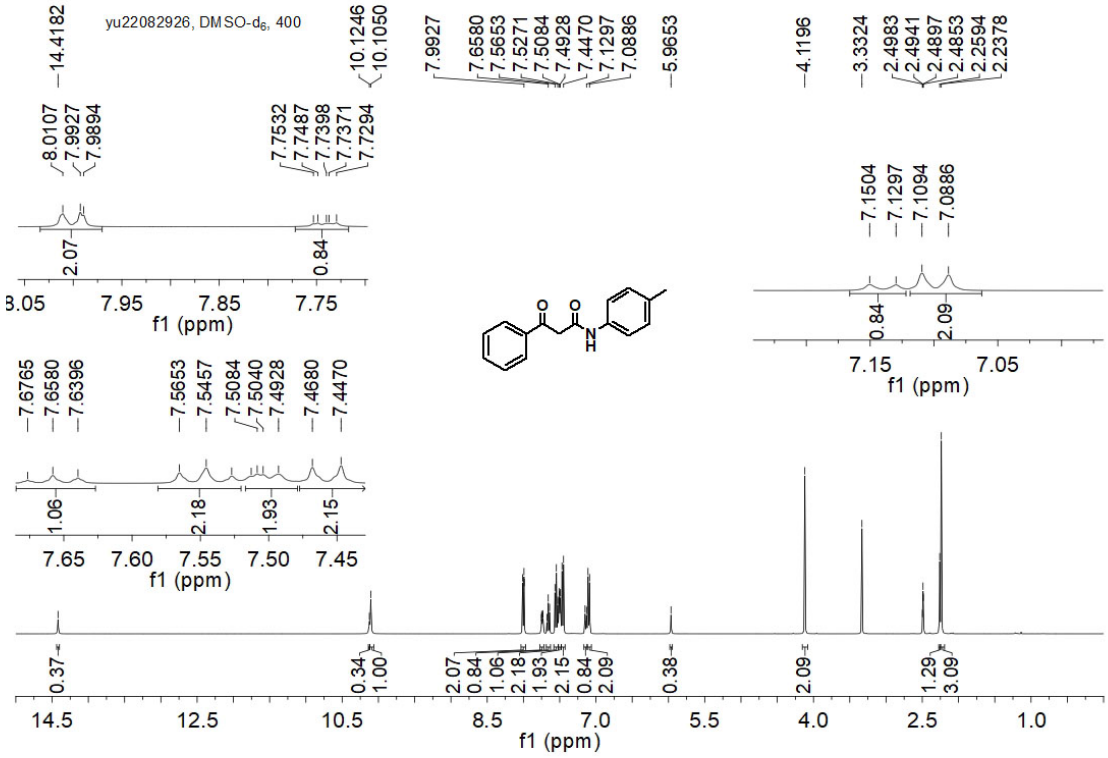

^{1}H NMR of **3m**

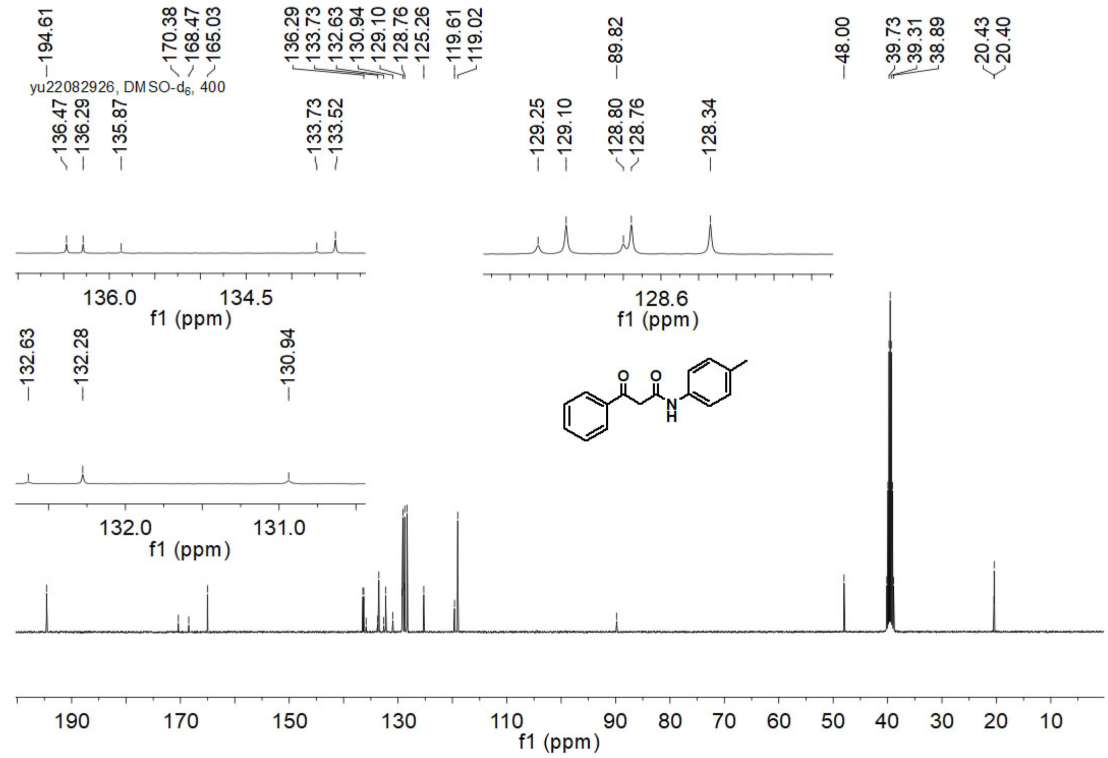

^{13}C NMR of **3m**

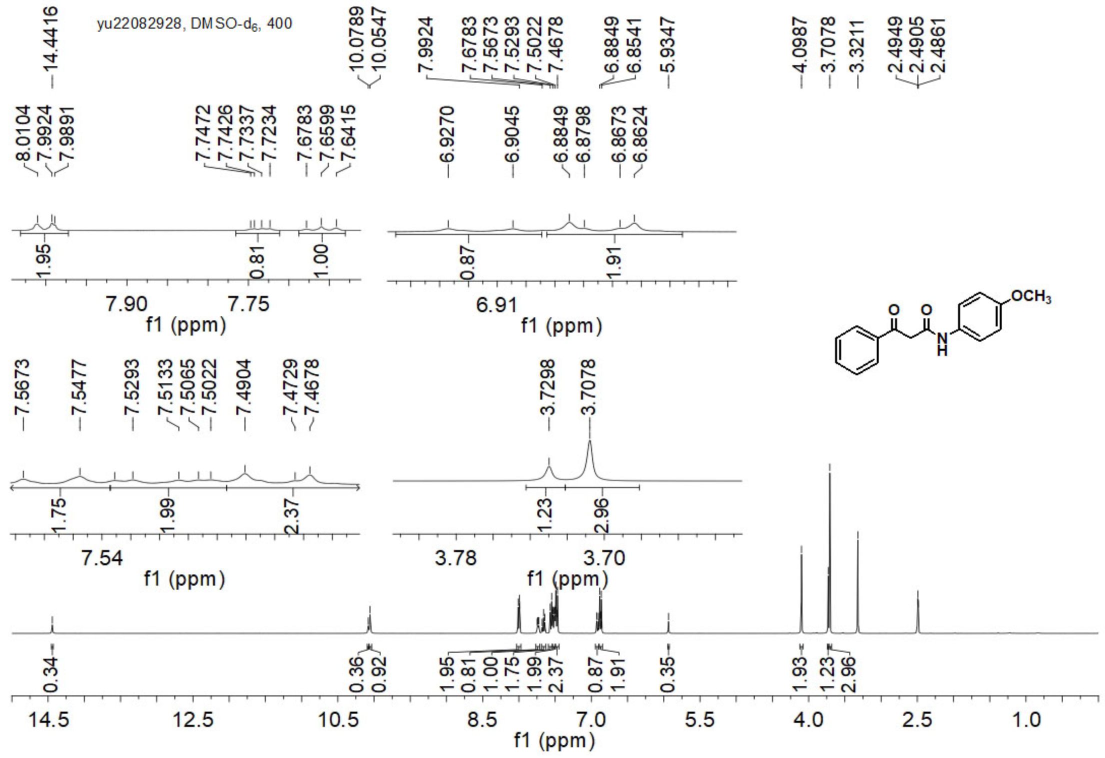

^{1}H NMR of **3n**

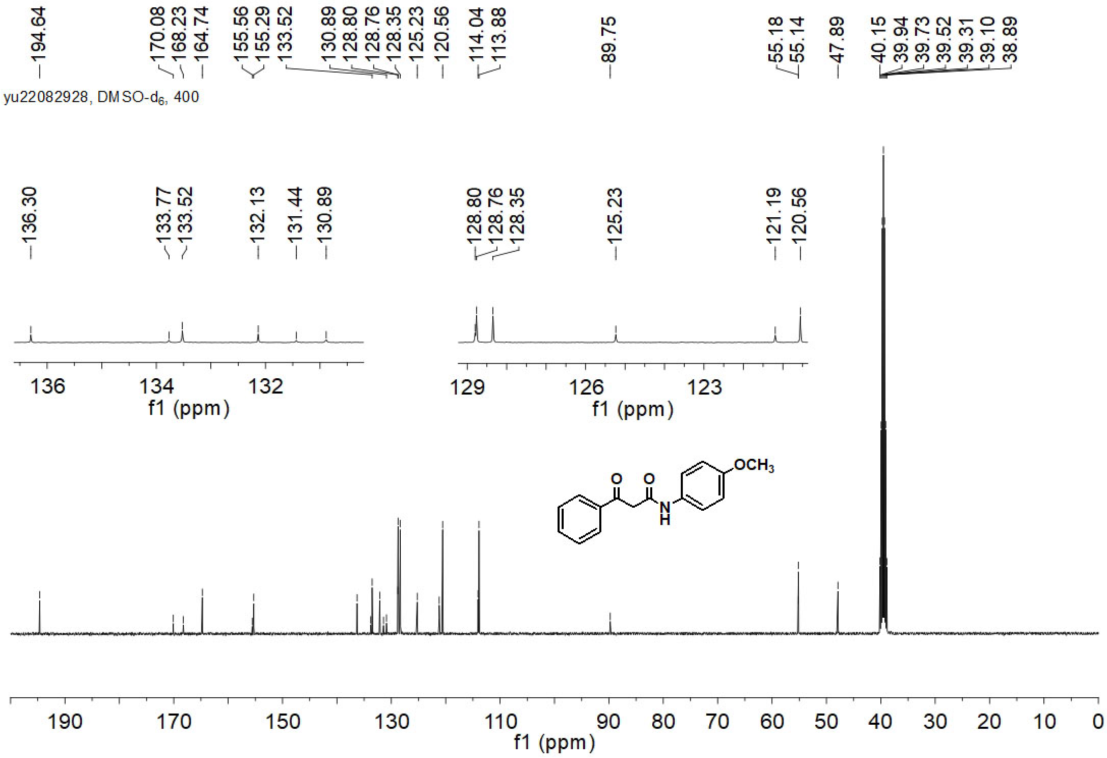

^{13}C NMR of **3n**

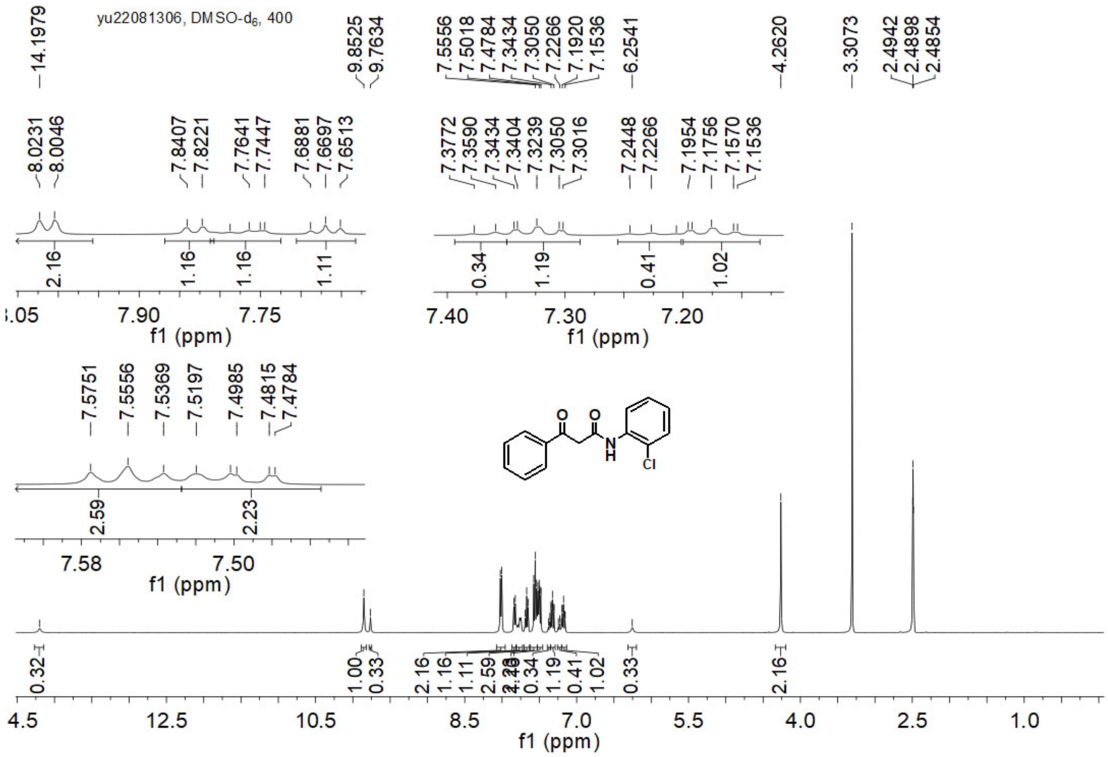

^{1}H NMR of **3o**

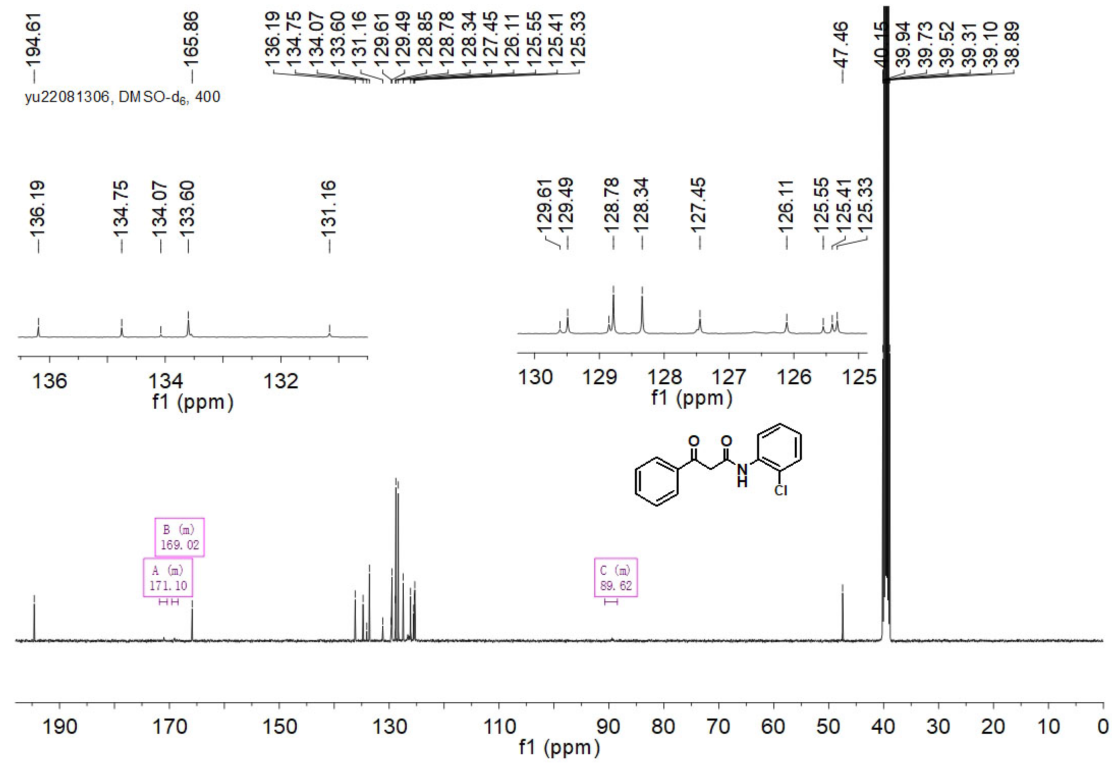

^{13}C NMR of **3o**

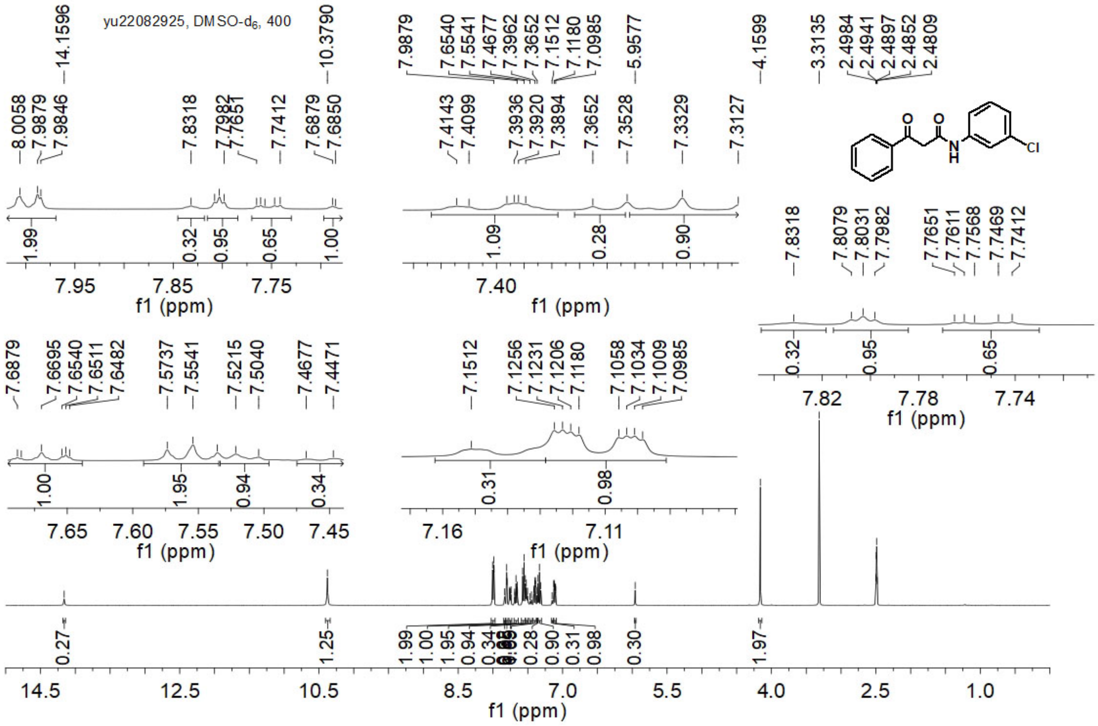

^{1}H NMR of **3p**

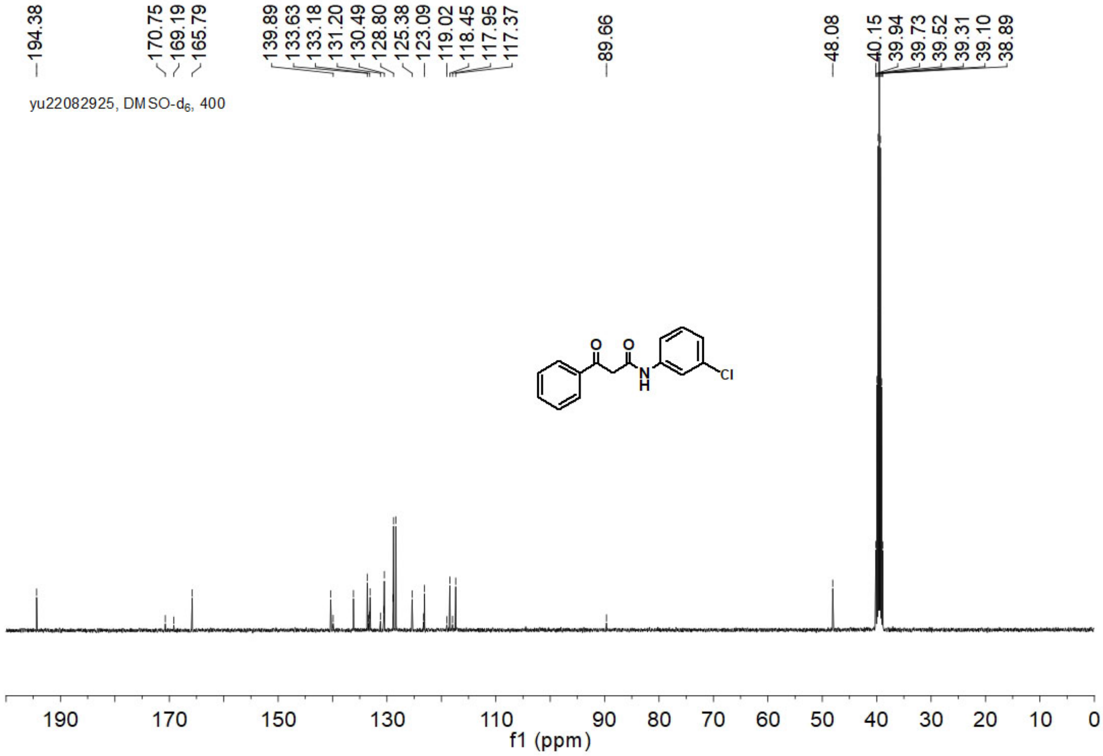

^{13}C NMR of **3p**

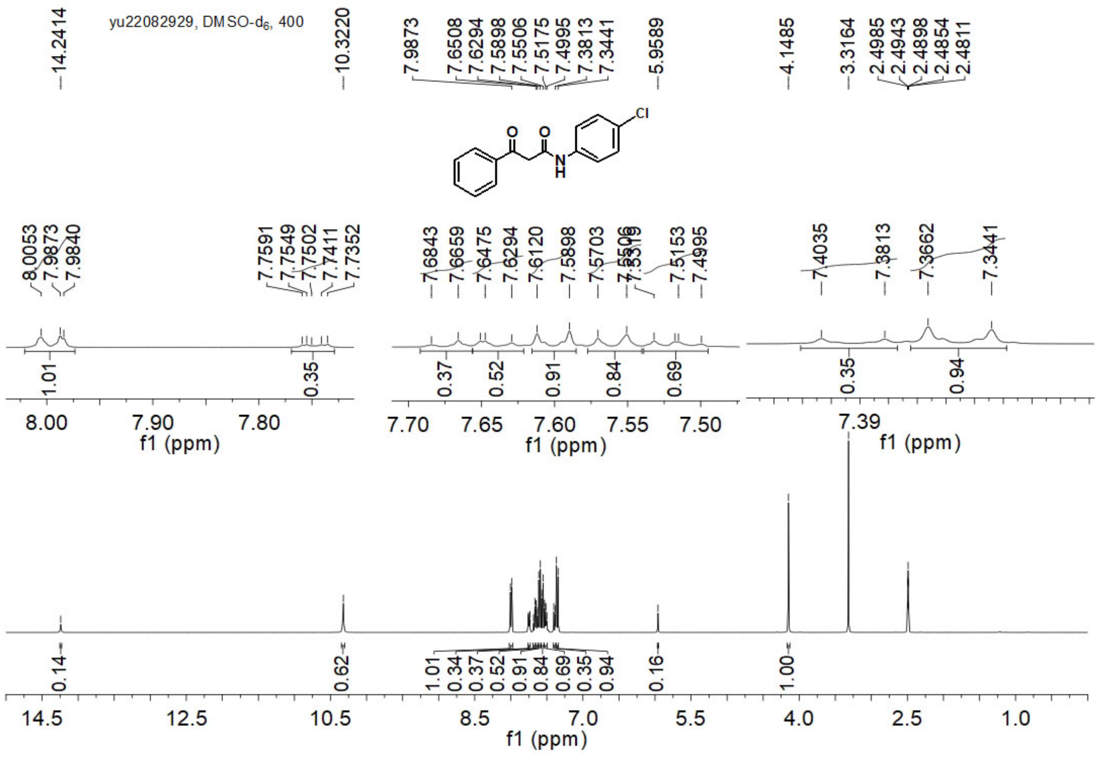

^{1}H NMR of **3q**

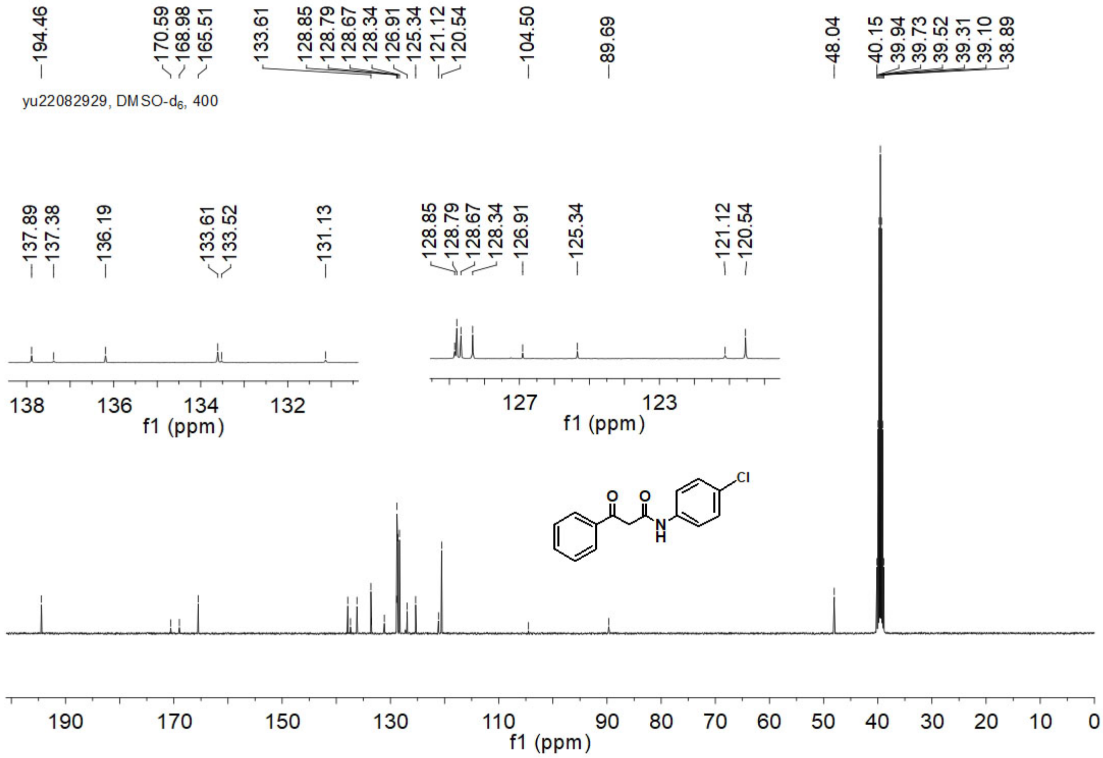

^{13}C NMR of **3q**

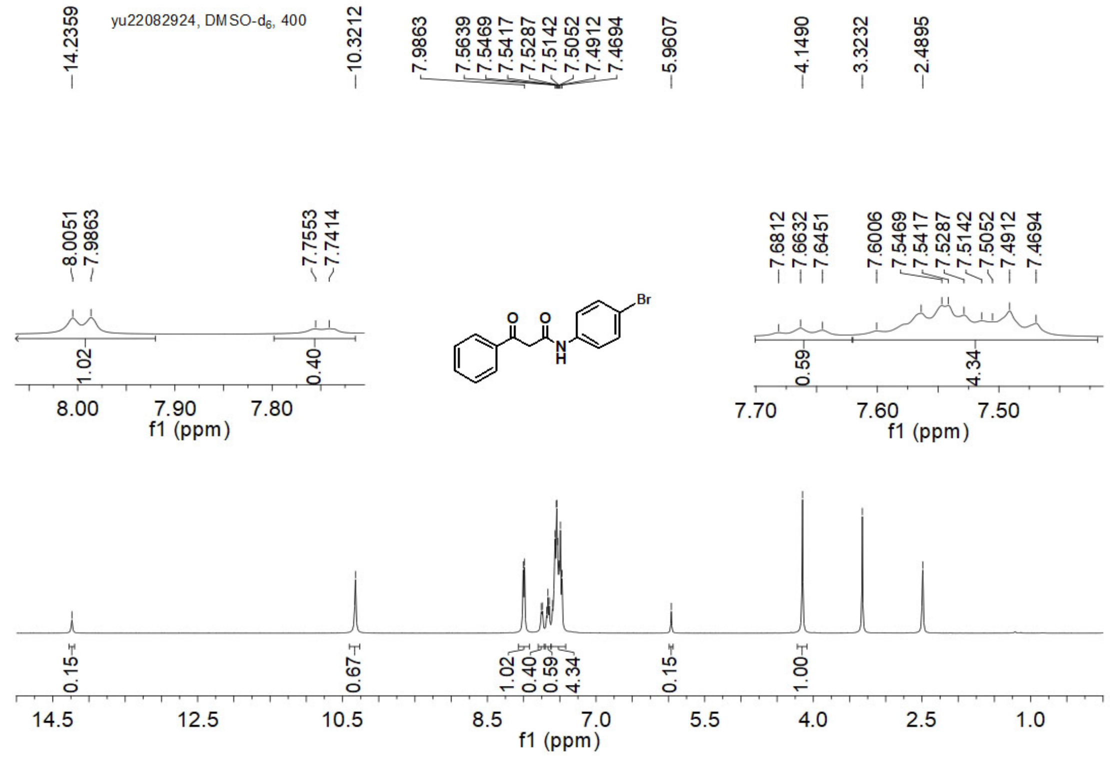

^{1}H NMR of **3r**

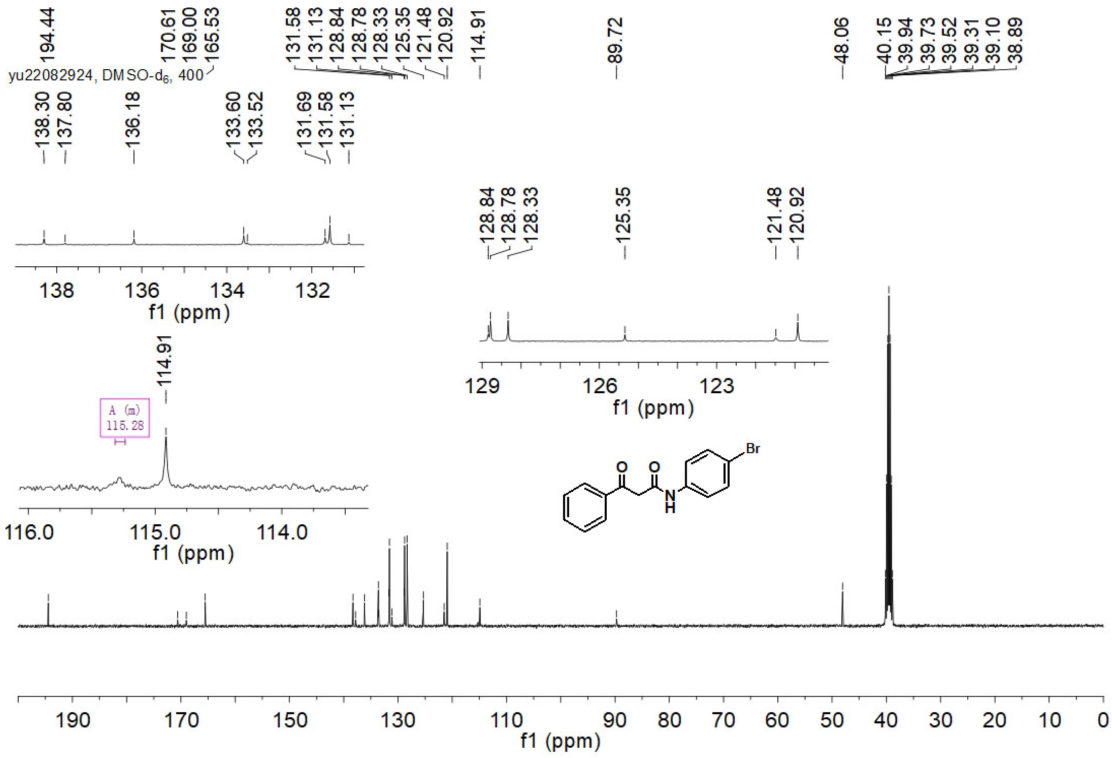

^{13}C NMR of **3r**

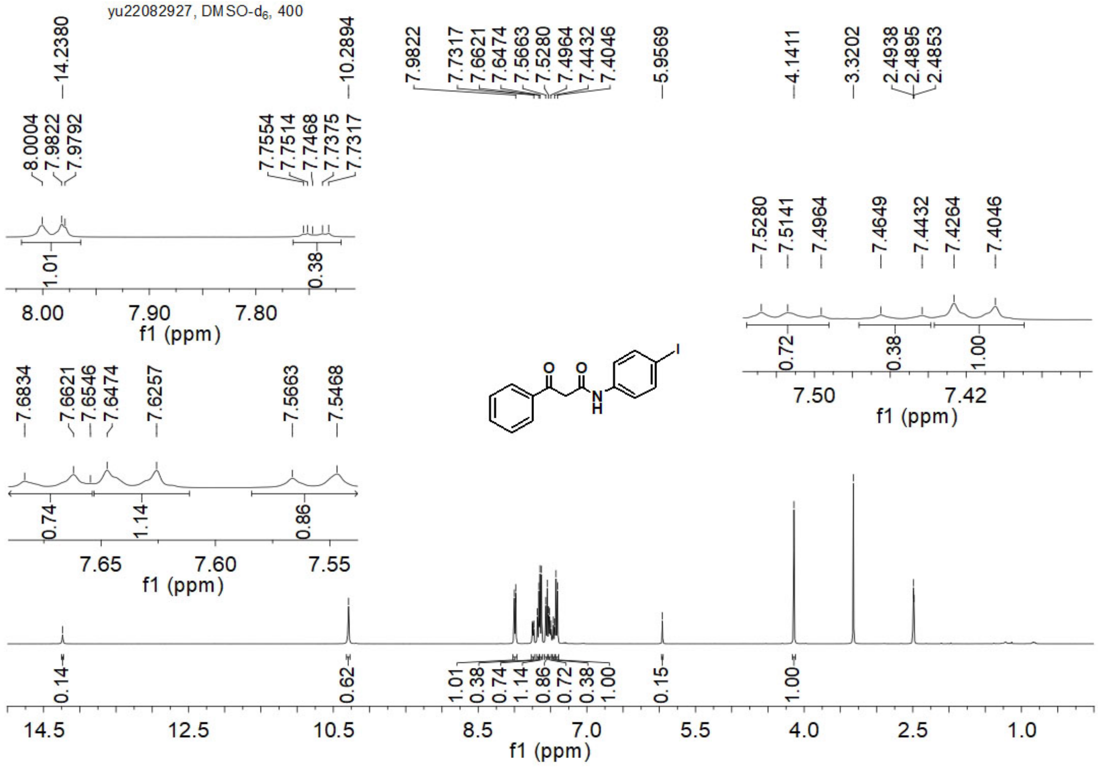

^{1}H NMR of **3s**

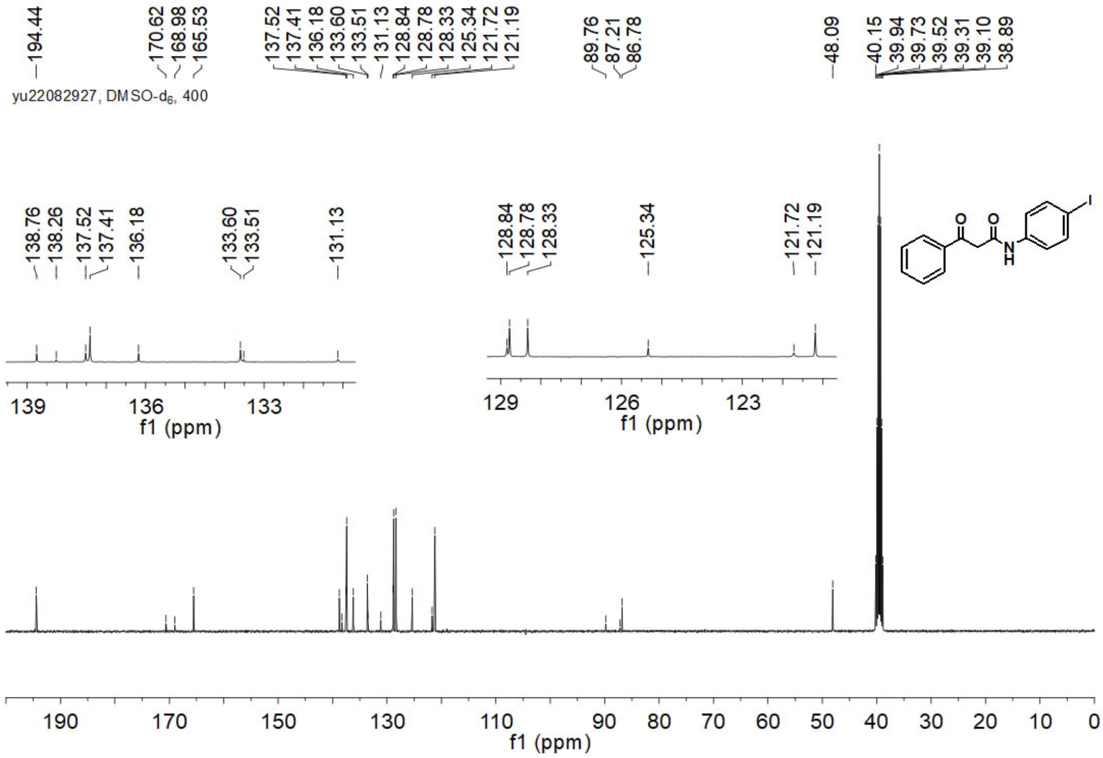

^{13}C NMR of **3s**

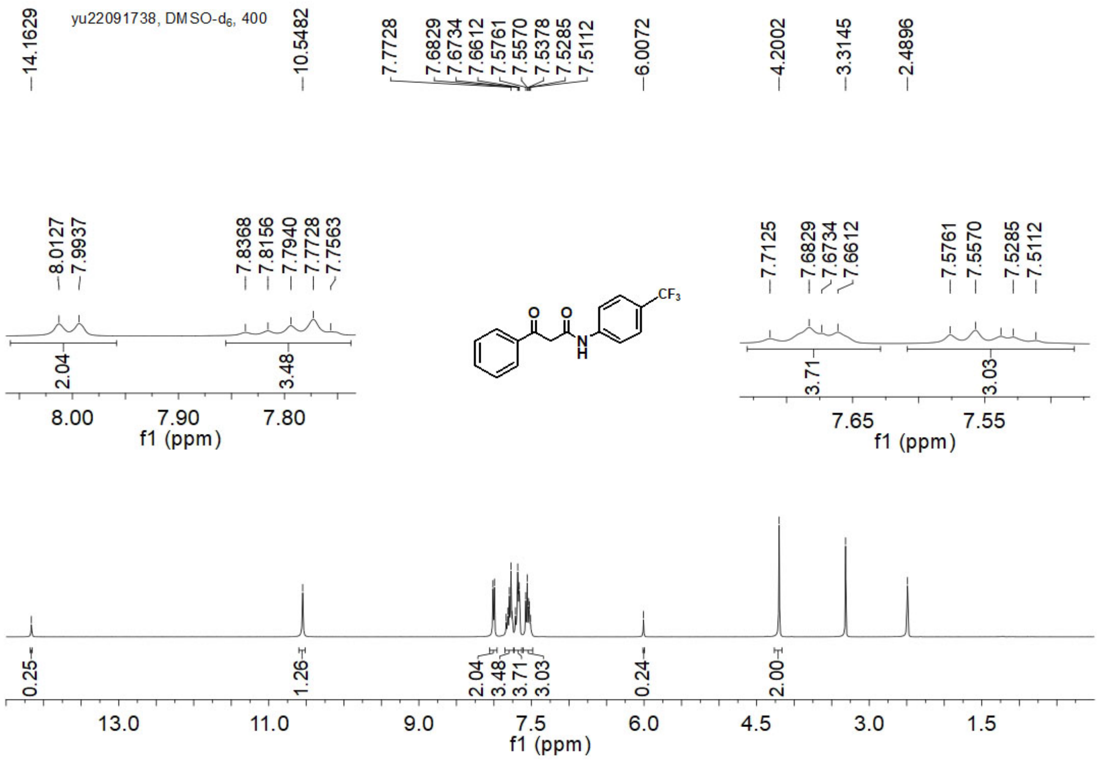

^{1}H NMR of **3t**

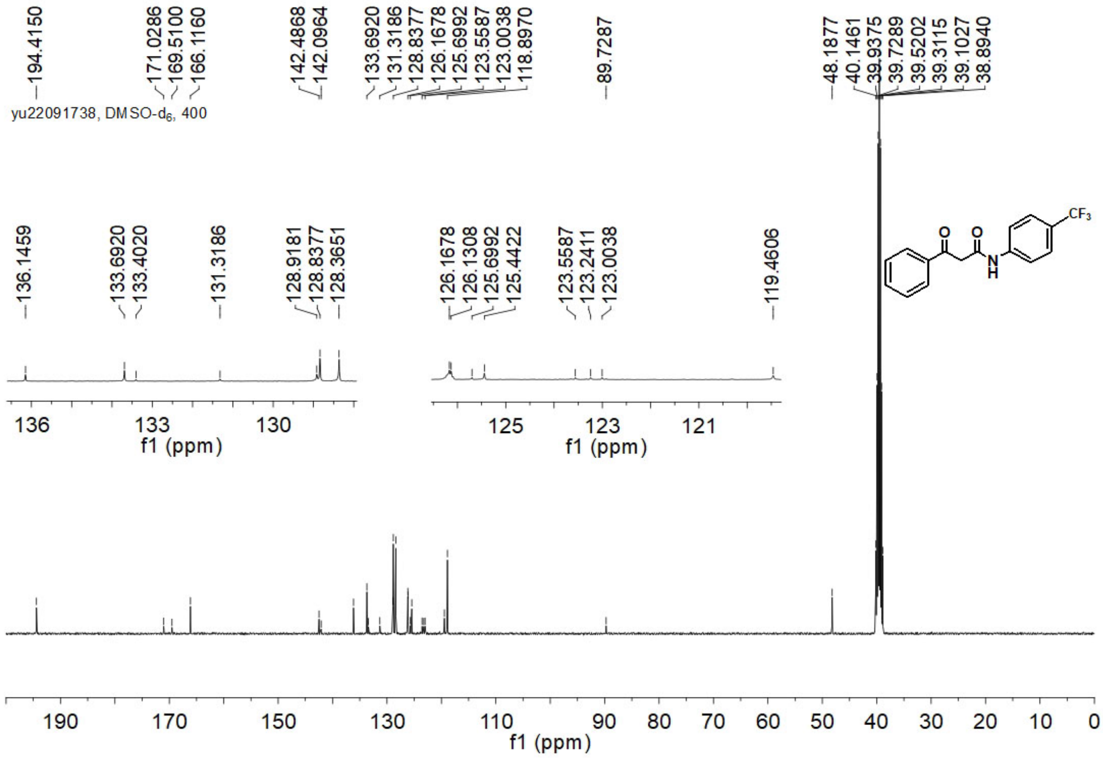

^{13}C NMR of **3t**

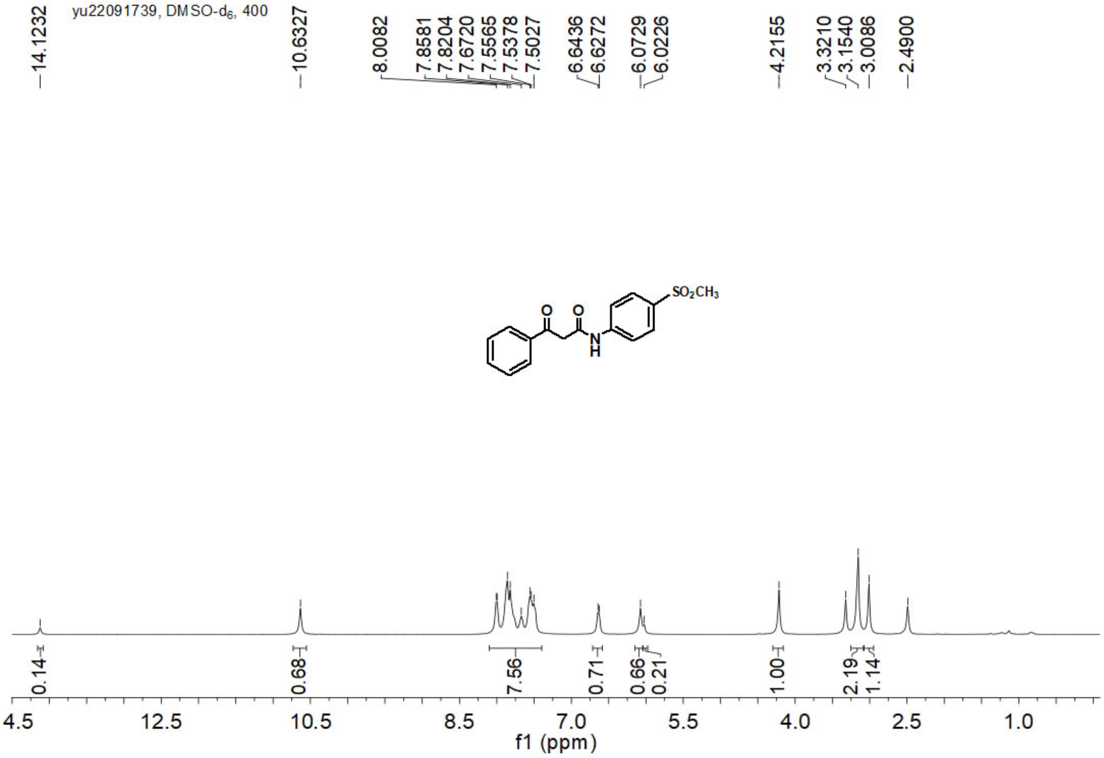

^{1}H NMR of **3u**

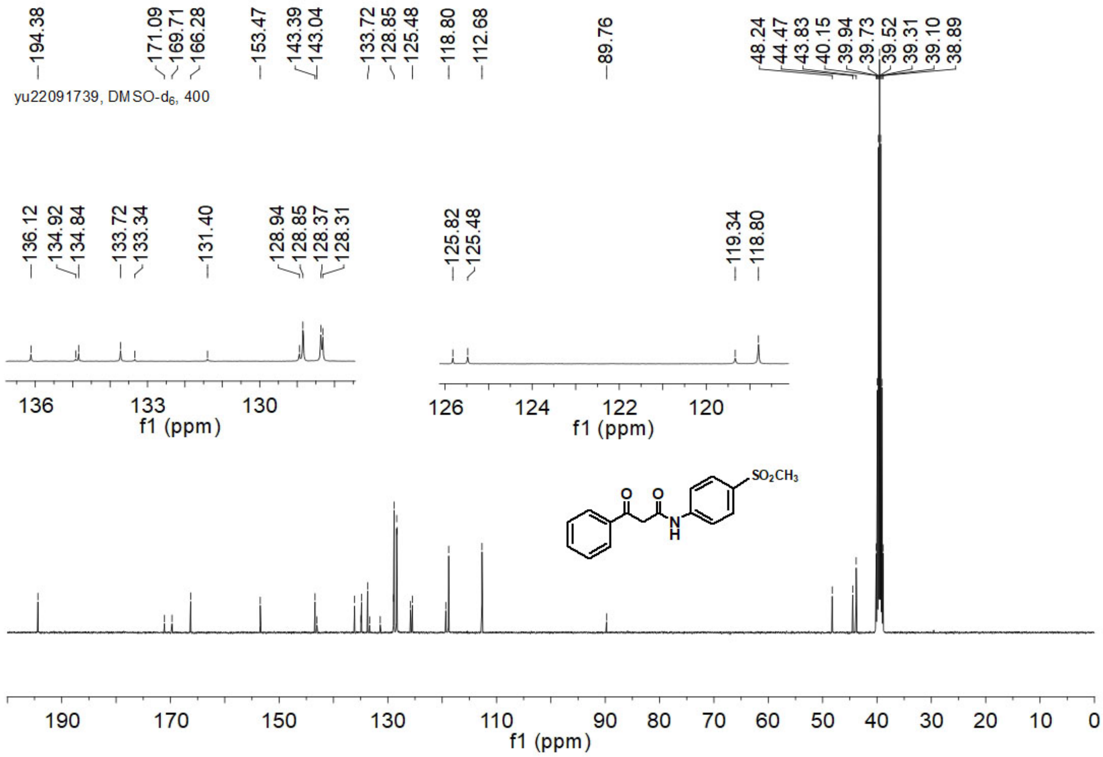

^{13}C NMR of **3u**

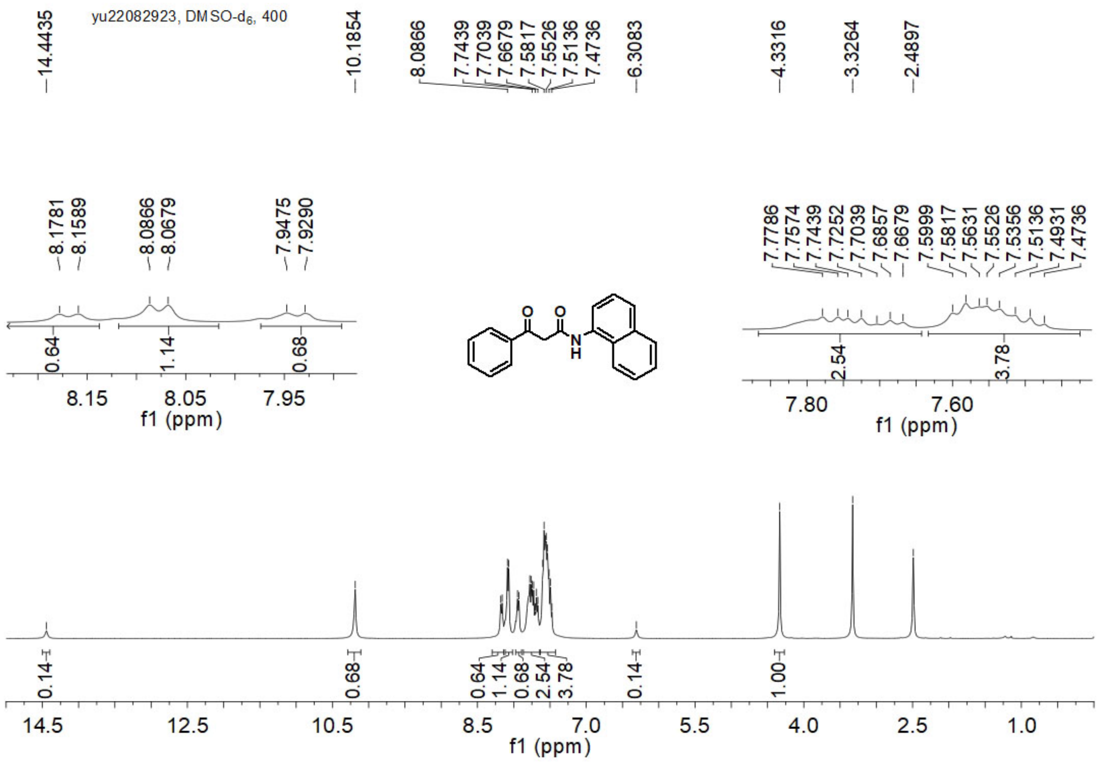

^{1}H NMR of **3v**

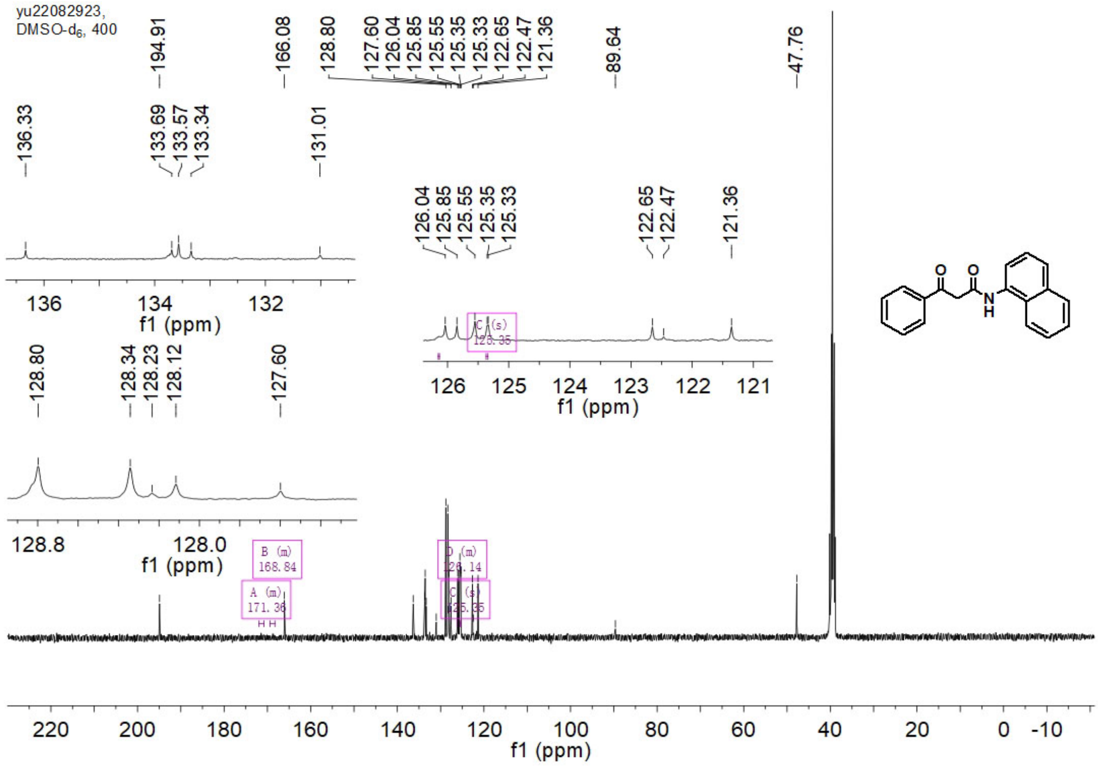

^{13}C NMR of **3v**

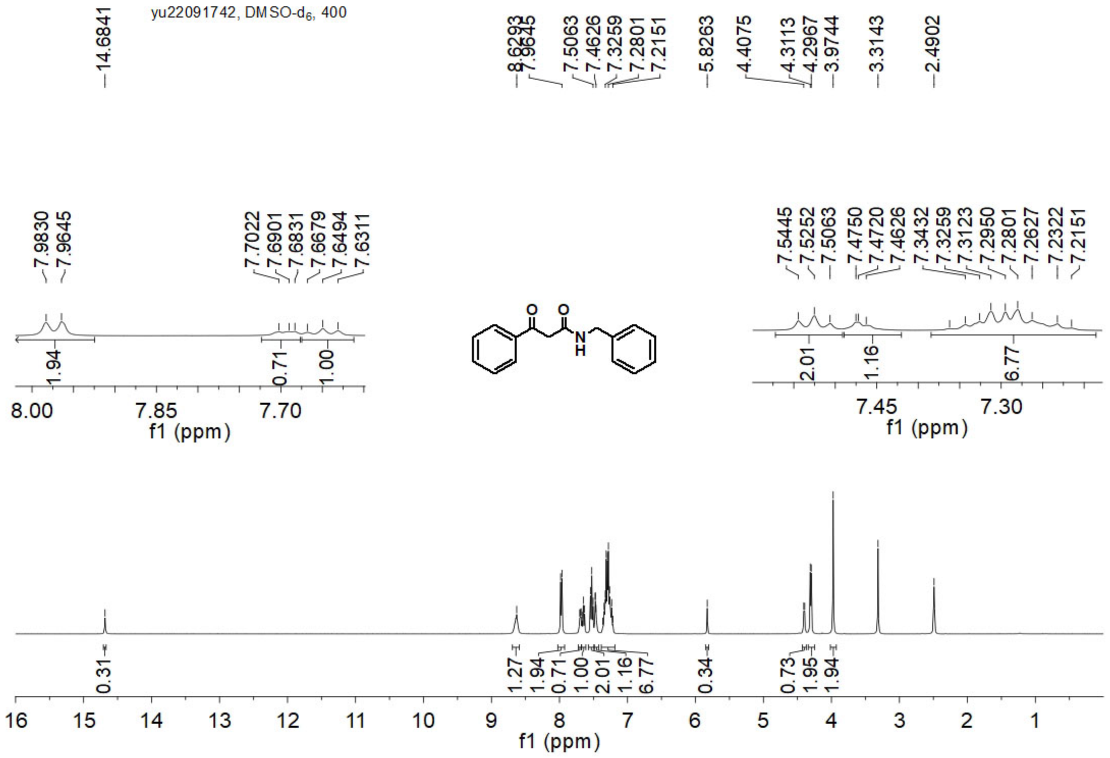

^{1}H NMR of **3w**

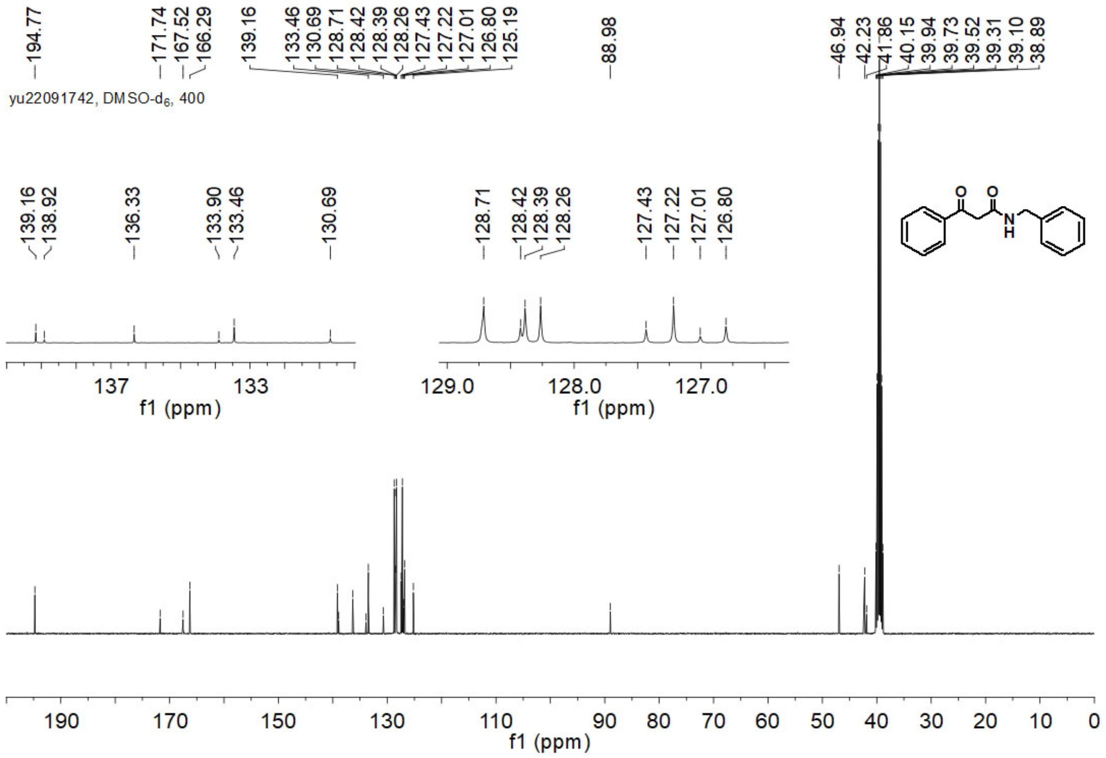

^{13}C NMR of **3w**
